# Computationally Designed Peroxygenases That Exhibit Diverse and Selective Terpene Oxyfunctionalization

**DOI:** 10.1021/acscatal.5c02412

**Published:** 2025-07-14

**Authors:** Judith Münch, Jordi Soler, Ofir Gildor-Cristal, Sarel J. Fleishman, Marc Garcia-Borràs, Martin J. Weissenborn

**Affiliations:** † Institute of Chemistry, 9176 Martin Luther-University Halle-Wittenberg, Weinbergweg 22, Halle (Saale) 06120, Germany; ‡ Institut de Qui’mica Computacional i Catàlisi and Departament de Qui’mica, 16738 Universitat de Girona, Carrer Maria Aurèlia Capmany 69, Girona 17003 Catalonia, Spain; § Department of Biomolecular Sciences, 34976 Weizmann Institute of Science, Rehovot 7600001, Israel

**Keywords:** computational chemistry, enzymes, FuncLib, oxyfunctionalization, *Saccharomyces cerevisiae*, terpenes, unspecific peroxygenase, yeast

## Abstract

The selective oxyfunctionalization of terpenes remains a major challenge in chemical synthesis and is of significant industrial importance. This study presents a computational enzyme design approach based on an AlphaFold2 model of an unspecific peroxygenase (*Mth*UPO). Using the FuncLib algorithm, only 50 variants were required, and they exhibit remarkable advancements. All 50 designs retained 100% measurable activity across the tested substrate panel, with each design showing activity on at least one substrate. Among the terpene substrates, improvements in activity varied considerably: while some substrates had only a single design exhibiting a ≥2-fold increase in activity, the top-performing substrate had 26 such designs. The most active design per terpene substrate showed enhancements ranging from 2.2-fold to 7.1-fold relative to the wild type. In addition to increased activity, many designs also demonstrated useful and dramatic shifts in regio-, chemo-, and stereoselectivity. Regioselectivity for the energetically less favored 3-hydroxy-β-damascone increased from 3 to 46%. Particularly striking is the dramatic improvement in chemoselectivity for the oxidation of geraniol and nerol to citral A (>99%) and citral B (89%), respectively. While wild-type *Mth*UPO exhibited only a moderate selectivity of 40% for citral A and 72% for citral B, our computationally designed variants displayed significantly enhanced product preference and up to a 4.5-fold increase in activity. Additionally, further products not found with the wild-type enzyme, such as isopiperitenol from limonene and epoxides from geraniol and nerol, were synthesized. For the hydroxylation of β-ionone, the enantioselectivity was inverted to a ratio of 1:99 from (*R*)- to (*S*)-4-hydroxy-β-ionone. FuncLib-enabled active-site remodeling allowed us to generate a small yet highly diverse enzyme panel that significantly outperformed the wild type across multiple synthetic challenges. The best-performing variants, such as design 4 and design 11 (both 4 mutations), exhibit improvements that result from epistatic effects. MD simulations demonstrated that these mutations collectively reshape the active site, allowing for regio- and chemoselectivities that are difficult to achieve by single-point mutations. Herein, we demonstrate the potential of in silico-guided approaches to rapidly develop highly selective biocatalysts for synthetic applications.

## Graphical abstract

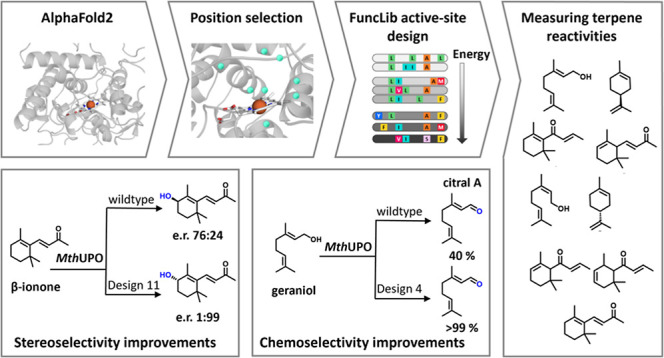

## Introduction

Selective oxyfunctionalization ranks among the most challenging and desirable reactions in synthetic chemistry. These reactions are often critical for selective C–H activation and alkene epoxidation of complex organic molecules.^1–4^ Since their discovery in 2004, fungal unspecific peroxygenases (UPOs) have attracted great interest for their ability to perform versatile oxyfunctionalization reactions on a broad scope of substrates.^5–7^ These enzymes have several advantages relative to other versatile oxidases, such as P450s:^8,9^ they are typically stable,^10^ use prereduced hydrogen peroxide as a cosubstrate instead of molecular oxygen and expensive reductants such as NAD­(P)­H, and exhibit a broad substrate scope^11^ and high turnover numbers (TON) up to 900,000.^12^ Despite their remarkable oxidative capabilities, unspecific peroxygenases (UPOs) also exhibit certain limitations. Many UPOs display only moderate regio- or enantioselectivity, which can restrict their application in fine chemical synthesis.^13,14^ Additionally, their broad substrate scope—while advantageous in some contexts—can lead to undesired side reactions such as overoxidation or low product specificity.^6,15^ These challenges highlight the importance of engineering strategies to fine-tune UPO selectivity and expand its practical utility in biocatalysis.

UPOs can be engineered for overexpression in rapidly proliferating host organisms, such as yeast, through the implementation of protein^16^ and signal-peptide^17^ engineering and promoter-shuffling techniques.^18^ Recent studies yielded enhanced variants characterized by increased activity,^19^ augmented thermo-,^20^ pH-,^21^ and solvent-stabilities,^20^ and large shifts in chemo-, regio-, and stereoselectivities.^22–27^ These studies frequently employed directed evolution,^16,23,26,28^ a prominent protein-engineering approach that emulates the engineering prowess of natural evolution through iterative rounds of random or semirational mutation and selection of variants that exhibit desirable properties.^29^ But despite yielding exceptional results, directed evolution is labor-intensive and time-consuming. It is especially impractical when seeking enzymes that exhibit improvements across multiple substrates, as mutations that are favorable for one substrate rarely benefit others.^30^ Furthermore, measuring oxyfunctionalization products is not amenable to chromogenic or fluorogenic measurement, limiting options for medium to high throughput screening, which is often essential to successful in vitro evolution campaigns. These limitations are currently addressed through small, “smart” libraries, using insights from molecular structures and mechanistic information, reducing the screening effort from thousands of variants to several hundred per round of evolution.^31–33^ As a pertinent example, we recently performed an engineering campaign starting from the UPO from (*Mth*UPO) resulting in two UPOs that selectively produced (*S*)- and (*R*)-4-hydroxy-β-ionone, with enantiomeric ratios of 97:3 (*R*) and 0.3:99.7 (*S*), respectively.^26^ This study establishes a benchmark for rational engineering of a challenging UPO: we screened in total 2,500 variants in three rounds of evolution, 75% of which displayed lower activity than the parental variant.

Here, we ask whether recent developments in computational protein design and engineering^34^ can address the limitations of rational engineering of enzyme active sites through a limited experimental effort.^35^ Such protein-design methods can be applied to crystallographic structures and can be generalized to AI-based model structures.^26,36,37^ For example, FuncLib is an automated method for designing diverse combinations of multipoint mutations within the active site of an enzyme.^37^ This method uses phylogenetic analysis and Rosetta atomistic design calculations to generate variants that exhibit large diversity in active-site geometry and electrostatics without impairing the stability, foldability, and primary activity of the enzyme. The resulting designs may exhibit strong epistatic dependencies among the mutations^37–39^ that are rarely observed in natural and lab-evolved variants; yet these designs exhibit diverse activities suggesting that FuncLib may uncover sequences and activities that are difficult for evolutionary processes to reach.

We focus on oxyfunctionalization of small terpenes which constitute the largest class of secondary plant metabolites.^40^ Terpenes and their oxyfunctionalized derivatives, terpenoids, frequently showcase pharmacological activity^41^ and find applications in the flavor and fragrance industries.^42,43^ Chemo-, regio-, and stereoselective oxygenation presents a challenge for chemical catalysis, given that many terpenes feature multiple sites where C–H hydroxylation and C=C epoxidation are chemically and energetically almost indistinguishable. Enzymes are naturally stereoselective and can position substrates to promote reactions at desired positions that are not kinetically favored or chemically activated; thus, accurate control of the UPO active-site pocket may enable oxyfunctionalization reactions that overcome the dictates of intrinsic chemical reactivity of the substrate.

Starting from an AlphaFold2 model of *Mth*UPO, we generated 50 *Mth*UPO FuncLib designs. All proved to be functionally secreted from yeast, allowing us to compare their influence on various substrates and to find large activity improvements and substantial shifts in chemo-, regio-, and stereoselectivity through a limited experimental screening effort (Figure 1). AI-based modeling and molecular dynamics simulations of substrate–enzyme pairs provided insights into some of the dramatic specificity changes that may extend to other UPOs.

**Figure 1:**
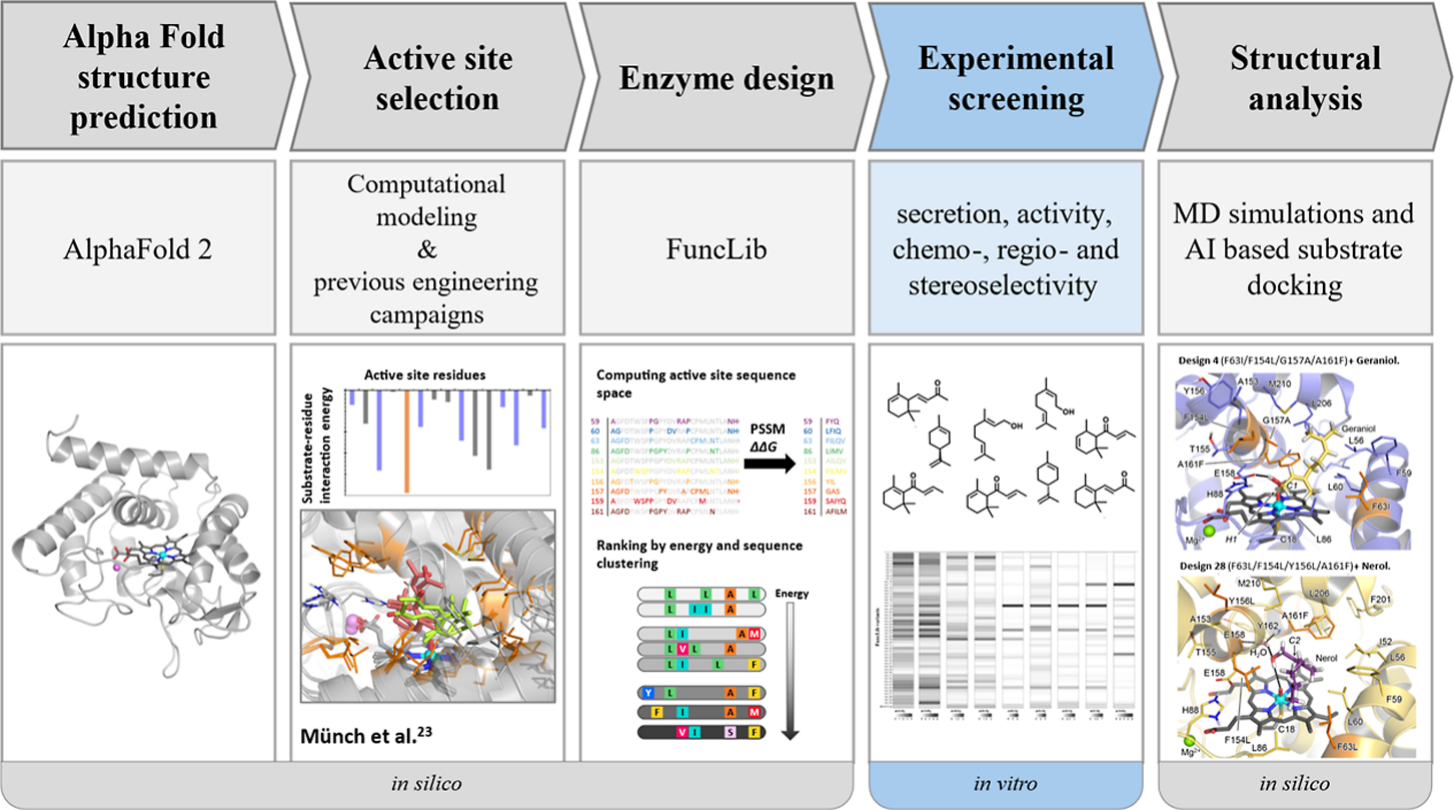
Overview of the different steps of the work protocol.

## Materials and Methods

### Chemicals

See the Supporting Information.

### Bacterial and Yeast Strains

For all cloning purposes and plasmid propagation, DH10B cells (ThermoFisher Scientific, Waltham, US) were utilized. All work regarding was performed utilizing the INV*Sc*1 strain (ThermoFisher Scientific, Waltham, MA, US).

### Oligonucleotides and Gene Parts

All oligonucleotides were purchased in the lowest purification grade “desalted” and minimal quantity at Eurofins Genomics (Ebersberg, DE). The genes of the *Mth*UPO FuncLib library were purchased as gene parts from Twist Bioscience (San Francisco, US).

### AlphaFold2 Model

The AlphaFold2 model of *Mth*UPO was extracted from the AlphaFold Protein Structure Database (UniProt entry G2QID2). Source: https://alphafold.ebi.ac.uk/entry/G2QID2 (16.02.2023).

### FuncLib Design

We used a C-terminally truncated (−18 residues) AlphaFold2 model of *Mth*UPO with its natural signal peptide automatically refined by the FuncLib algorithm (using Rosetta) as the protein structure. The loop at the original C-terminus entered the substrate channel and active side during the simulations, impeding residue variation wherefore we used the truncated model for further calculations. We subsequently defined positions C18 (axial ligand) as well as E158 and H88 (catalytic cascade) as essential amino acids which must not be subject to any variation. The number of mutations per design was set to two to four with at least two variations between each design. We did not apply an algorithm like PROSS^44^ for introducing stabilizing mutations into the enzyme before running the FuncLib campaign, even though introducing several mutations in the active side may compromise enzyme stability. In our previous work with *Mth*UPO, we did not encounter stability issues after introducing mutations. Further prior work on *Aae*UPO variant PADA-I showed that an aggressive FuncLib campaign on unspecific peroxygenase can be tolerated without introducing further stabilizing mutations beforehand.^38^ For diversification, positions were selected that enhanced activity, chemo-, regio-, and stereoselectivity of *Mth*UPO for different substrates in prior directed evolution campaigns.^22,25,26^ Altering positions F59, L60, F63, A153, F154, Y156, G157, S159, A161, L206, and M210 showed substantial influence in previous studies, and we additionally selected position L86 due to its proximity to the active site. In total, we selected positions F59, L60, F63, L86, A153, F154, Y156, G157, S159, A161, L206, and M210 to be diversified. Charged amino acids (Arg, His, Lys, Glu, and Asp) were excluded from the allowed sequence space to keep the hydrophobic character of the active site. Thr was also excluded as including all mutations to the initial sequence space file, that were found in previous projects (e.g., F59Q), exceeded the maximum permitted number of variants. The highly polar amino acid Thr was not considered to be of most interest for the project compared to other more hydrophobic amino acids. Initial FuncLib runs showed steric overlap of mutations containing aromatic identities at position L86, mutations larger than Ala and Ser at position G157 might obstruct access to the heme, and the mutation A161F appeared to clash with the presumed heme site; therefore, such mutations were prohibited. In the future, the usage of AlphaFill or AlphaFold3 to include heme into the active site could prevent the introduction of mutations such as the aforementioned. Finally, the following sequence space was explored: Table S1.

### Expression Plasmids

Expression plasmids for *Mth*UPO expression in were constructed as previously described—all parts are available at AddGene.^17^ The gene parts of the FuncLib library were first cloned as individual level 0 standard modules into the universal level 0 acceptor plasmid (pAGM9121) and afterward released upon BsaI restriction digest. Golden Gate reactions were performed to combine the *Mth*UPO FuncLib genes with the *Sce*-α.galactosidase signal peptide and a TwinStrep-GFP11-Tag for purification and secretion detection in a level 1 expression plasmid (pAGT572).

Microtiter plate cultivation of , shake flask cultivation of , supernatant ultrafiltration and protein purification, and heme–CO complex measurements were performed as described previously.^17^

### Colorimetric Screening Assays

Four colorimetric assays were performed: ABTS, DMP, NBD, and splitGFP assay. All assays were performed as described before.^45^ For NBD, ABTS, and DMP, the absorbance difference between five minutes (*t*1) and 0 minutes (*t*0) was evaluated. The DMP assay was performed with enzyme secreted in a medium without additional heme to avoid a background reaction.

### Bioconversion in a Microtiter Plate

All reactions with noncolorimetric substrates were initially performed in a microtiter plate. 100 μL of supernatant derived from enzyme expression and secretion in in a microtiter plate was transferred to a 96-deep-well plate (CR1496, EnzyScreen, Heemstede, NL). 400 μL of a reaction Mastermix was added to achieve final concentrations of 1 mM substrate, 1 mM H_2_O_2_, 5% acetone, and 100 mM Kpi (pH 7.0). Reactions were performed for 1 h at 30 °C under continuous shaking at 300 rpm. The extraction was accomplished through the addition of 500 μL of EtOAc (GC Ultra grade) containing 0.25 mM of an internal standard (Table S3) and further shaking for 30 min at 25 °C and 300 rpm. Microtiter plates were centrifuged to separate the phases (3000 rpm, 10 min) and 300 μL of the organic phase was transferred to a glass-coated microtiter plate utilizing the Platemaster (Gilson, Middelton, US) for subsequent GC–MS analysis.

### Bioconversion in Single Vials

All bioconversions for regioselectivity determination were performed in triplicates in single vials with direct addition of H_2_O_2_. 250 nM enzyme supernatant, derived from enzyme expression and secretion in in a shake flask after ultrafiltration and concentration determination was transferred to a glass vial. A reaction Mastermix was added to achieve final concentrations of 1 mM substrate, 5% acetone, 1 mM H_2_O_2_, and 100 mM Kpi (pH 7.0) to give a total volume of 500 μL. Reactions were performed for 1 h at 30 °C under continuous shaking. The extraction was accomplished through the addition of 500 μL of EtOAc (GC Ultra grade) containing 0.25 mM of an internal standard (Table S3), and the organic phase was transferred to a new glass vial for subsequent GC–MS analysis.

### Bioconversion in Single Vials with a Syringe Pump

All bioconversions for TON determination were performed in triplicates in single vials utilizing a syringe pump system. 250 nM enzyme supernatant, derived from enzyme expression and secretion in in a shake flask after ultrafiltration and concentration determination, was transferred to a glass vial. A reaction Mastermix was added to achieve final concentrations of 1 mM substrate, 5% acetone, and 100 mM Kpi (pH 7.0) to give a total volume of 400 μL. 100 μL of H_2_O_2_ (stock solution 5 mM, final concentration 1 mM) was added over the period of the reaction via a syringe pump. Reactions were performed for 1 h at 30 °C under continuous shaking. The extraction was accomplished through the addition of 500 μL of EtOAc (GC Ultra grade) containing 0.25 mM of an internal standard (Table S3), and the organic phase was transferred to a new glass vial for subsequent GC–MS analysis.

### GC–MS Analysis

All GC–MS measurements were performed on a Shimadzu GCMS-QP2010 Ultra (Shimadzu, Kyoto, JP) with helium as carrier gas. The detector voltage of the secondary electron multiplier was adjusted in relation to the tuning results with perfluorotributylamine. The GC–MS parameters were controlled with GCMS Real Time Analysis, and for data evaluation, GCMS Postrun Analysis (GCMSsolution Version 4.45, Shimadzu, Kyoto, JP) was used. The temperature programs utilized are listed in Table S3. Ionization was obtained by electron impact with a voltage of 70 V. Calibration and quantification were implemented in scan mode.

### Nonchiral Gas Chromatography–Mass Spectrometry (GC–MS)

Measurements were performed on an SH-Rxi-5Sil MS column (30 m × 0.25 mm, 0.25 μm film, Shimadzu, Kyoto, JP). One microliter of each sample was injected with a split ratio of 1:20 (inlet temperature 200 °C). The temperature of the ion source was 280 °C. All initial screening measurements were performed in single replicates.

### Chiral Gas Chromatography–Mass Spectrometry (GC–MS)

Measurements were performed on a Lipodex E column (25 × 0.25 mm, Macherey-Nagel, Düren, DE). One microliter of each sample was injected with a split ratio of 1:10 (inlet temperature 200 °C). The temperature of the ion source was 200 °C.

### Quantum Mechanics (QM) Calculations

A truncated computational model was used to model the C–H activation and epoxidation of geraniol (1) and nerol (2) substrates. The truncated model [Fe=O­(Por)­(SCH_3_)­(substrate)] includes the active Fe-oxo species (Fe=O), the porphyrin pyrrole core (Por), a methyl thiolate group (-SCH_3_) to mimic cysteine axial ligand, and 1 or 2 as substrate. Density Functional Theory (DFT) calculations were carried out using the Gaussian16 software package.^46^ Geometry optimizations and frequency calculations were performed using the unrestricted hybrid (U)­B3LYP^47–49^ functional with an ultrafine integration grid^50^ and including the CPCM polarizable conductor model (dichloromethane, ε = 8.9)^52,53^ to have an estimation of the dielectric permittivity in the enzyme active site.^53^ The 6-31G­(d) basis set was used for all atoms but Fe, where the SDD basis set and related SDD pseudopotential were employed. The optimized geometries were verified as minima by vibrational frequency analysis, and transition state geometries have a single imaginary frequency consistent with the reaction coordinate. Enthalpies and entropies were calculated for 1 atm and 298.15 K. A correction to the harmonic oscillator approximation, as discussed by Truhlar and co-workers,^54,55^ was also applied to the enthalpy calculations by raising all frequencies below 100 cm^–1^ to 100 cm^–1^ using the Goodvibes v.1.0.1 Python script.^56^ Single-point energy calculations were performed using the functional (U)­B3LYP with the Def2TZVP basis set on all atoms and within the CPCM polarizable conductor model (dichloromethane, ε = 8.9)^51,52^ and an ultrafine integration grid.^50^ Empirical Grimme D3 dispersion corrections with Becke–Johnson (GD3BJ) damping are also included in single-point calculations.^57^ All structures have a total neutral charge and calculations were performed with doublet (d) or quartet (q) multiplicities consistent with the expected electronic states of the Fe. Figures of DFT structures were rendered using CYLview^58^ and MolUP VMD extension^59^ was used for output visualization.

### Homology Model and Molecular Dynamics (MD) Simulations

The homology model for the *Mth*UPO structure (283 residues) obtained from our previous work has been used as the starting point.^22^ Mutations were introduced using the Mutagenesis tool in PyMOL.^60^ Molecular Dynamics (MD) simulations in explicit water were performed using the AMBER18 package.^61,62^ Parameters for the geraniol (**1**) and nerol (**2**) substrates were generated within the antechamber^63^ module in the AMBER18 package using the general AMBER force field (gaff2),^64^ with partial charges set to fit the electrostatic potential generated at the B3LYP/6-31G­(d) level by the RESP model.^65^ The charges were calculated according to the Merz–Singh–Kollman scheme^66,67^ using the Gaussian16 package. Parameters for the heme compound I (Cpd I) and the axial Cys were taken from ref 68. The protein was solvated in a pre-equilibrated cubic box with a 12 Å buffer of TIP3P^69^ water molecules using the AMBER18 leap module, resulting in the addition of ∼12,500 solvent molecules. The systems were neutralized by the addition of explicit counterions (Na^+^ and Cl^–^). All subsequent calculations were done using the AMBER force field 14 Stony Brook (ff14SB).^70^ A two-stage geometry optimization approach was performed. The first stage minimizes the positions of solvent molecules and ions imposing positional restraints on the solute by a harmonic potential with a force constant of 500 kcal·mol^–1^ Å^–2^, and the second stage is an unrestrained minimization of all of the atoms in the simulation cell. The systems were gently heated using six 50 ps steps, incrementing the temperature by 50 K for each step (0–300 K) under constant-volume and periodic-boundary conditions. Water molecules were treated with the SHAKE algorithm such that the angle between the hydrogen atoms was kept fixed. Long-range electrostatic effects were modeled using the particle-mesh-Ewald method.^71^ An 8 Å cutoff was applied to Lennard-Jones and electrostatic interactions. Harmonic restraints of 30 kcal·mol^–1^ were applied to the solute, and the Langevin equilibration scheme was used to control and equalize the temperature. The time step was kept at 1 fs during the heating stages, allowing potential inhomogeneities to self-adjust. Each system was then equilibrated for 2 ns with a 2 fs time step at a constant pressure of 1 atm and a temperature of 300 K without restraints. Once the systems were equilibrated in the *NPT* ensemble, production trajectories were then run under the *NVT* ensemble and periodic-boundary conditions. In particular, a total of 1500 ns from 3 independent replicas (500 ns each) were accumulated for each of the following systems: design 2 (F63L/A153I/F154I/G157A), design 4 (F63I/F154L/G157A/A161F), design 26 (F63I/F154I/G157A/A161L), and design 28 (F63L/F154L/Y156L/A161F). Trajectories were processed and analyzed using the CPPtraj^72^ module from AmberTools utilities. VMD visualization software was used to visualize MD simulations.^73^ Protein structures were rendered using PyMOL.^60^

### Docking and Protocol Used for Substrate-Bound MD Simulations

Docking calculations were performed using AutoDock Vina.^74^ The most populated clusters (based on backbone clustering analysis) obtained from MD simulations carried out in the absence of a substrate were used, and docking predictions were then utilized as starting points for substrate-bound MD simulations. The following systems were prepared: design 4 + geraniol, design 2 + geraniol, design 26 + nerol, and design 28 + nerol. The same protocol for MD simulations described above has been employed. Three replicas of 500 ns were carried out on each system without any external restraints on the substrate, thus accumulating a total of 1500 ns for each system. Trajectories were processed and analyzed using the CPPtraj28 module from the AmberTools utilities. VMD visualization software was used to visualize MD simulations.^73^ Protein structures were rendered using PyMOL.^60^

### AI-Based Docking for Design/Substrate Modeling

Models for each design/substrate pair were generated using Chai-1 (downloaded from https://github.com/chaidiscovery/chai-lab), an AlphaFold3-based and open-access predictor of biomolecular interactions. Each docking trajectory produces five docked models, and we launched several runs with different seeds to generate up to 50 models per design pair (Table S11).

We used the Simplified Molecular Input Line Entry System (SMILES) notation for heme-oxo species (compound I) for modeling:

C=CC1=C(C)C2=CC(C(C)=C3CCC([O‐])=O)=[N]4C3=CC5=[N]6C(C(C)=C5CCC([O‐])=O)=CC7=C(C=C)C(C)=C(C=C1[N‐]28)[N‐]7[Fe]468=O

In addition, we used SMILES notations extracted from PubChem for each of the terpenes (Table S11).

Models that exhibited ≤4.5 Å between the oxidized carbon and compound I oxygen were defined as forming a near-attack conformation (NAC). Models that exhibited a ≤3.4 Å distance between any atoms on the ligand and the enzyme were considered as forming steric overlaps.

### Preparative Work

#### 2,3-Epoxy Nerol

Nerol (590 mg, 3.8 mmol) was dissolved in acetone (7.5 mL) and poured into a solution of potassium phosphate buffer (100 mM, 111 mL, pH 7.0) and 250 nM *Mth*UPO Var 28 (stock solution of 20.5 μM, 1.8 mL). Thirty millimolar hydrogen peroxide (stock solution 150 mM, 30 mL) was added via a syringe pump over the period of the reaction time. The solution (total: 150 mL) was stirred at 30 °C, overnight. Afterward, the mixture was extracted three times with ethyl acetate. The organic phase was washed with brine, dried with sodium sulfate, filtered, and concentrated under reduced pressure. The crude product was purified by column chromatography on silica gel using *n*-hexane/ethyl acetate (9/1 → 2/1) obtaining 156 mg (70%) of 2,3-epoxy nerol as a pale-yellow oil. GC–MS analysis showed impurities of silicone grease leading to a total purity of 85% which corresponds to a yield of 132 mg (55%) of 2,3-epoxy nerol.

^1^H NMR (400 MHz, CDCl_3_) δ: 5.07 (t, *J* 8 Hz, 1H), 3.37 (m, 1H), 3.65 (m, 1H), δ: 2.94 (q, *J* 4 Hz, 1H), 2.17–2.01 (m, 2H), 1.68–1.63 (m, 4H), 1.60 (s, 3H), 1.33 (s, 3H).

#### Isopiperitenol

(*S*)-(−)-Limonene (360 mg, 2.4 mmol) was dissolved in acetone (7.5 mL) and poured into a solution of potassium phosphate buffer (100 mM, 93 mL, pH 7.0) and 200 nM *Mth*UPO Var 28 (stock solution of 1.5 μM, 20 mL). Twenty millimolar hydrogen peroxide (stock solution 100 mM, 30 mL) was added via a syringe pump over the period of the reaction time. The solution (total: 150 mL) was stirred at 30 °C, overnight. Afterward, the mixture was extracted three times with ethyl acetate. The organic phase was washed with brine, dried with sodium sulfate, filtered, and concentrated under reduced pressure. The crude product was purified by column chromatography on silica gel using *n*-hexane/ethyl acetate (19/1 → 5/1) obtaining 3 mg (1%) of isopiperitenol as a pale-yellow oil. GC–MS analysis showed impurities of silicone grease leading to an even lower yield of isolated product.

^1^H NMR (400 MHz, CDCl_3_) δ: 5.42 (s, 1H), 4.86 (d, *J* 20 Hz, 2H), 4.15–4.05 (m, 2H), 2.11 (s, 1H), 2.05 (m, 1H), 1.71 (s, 3H), 1.68 (s, 3H), 1.41 (d, *J* 8 Hz, 2H).

## Results

### Modeling and Design Calculations

Because the molecular structure of *Mth*UPO has not been determined experimentally, we started from an AlphaFold2 model (UniProt entry: G2QID2) of the core enzymatic domain (amino acid positions 1–227).^75^ Because AlphaFold2 does not model cofactors, we compared the model to the structure of an artificial UPO (artUPO, PDB entry: 7ZNV), a homologue from *Marasmius rotula* (*Mro*UPO).^76^ Visual inspection verified that the side chain conformations of amino acids that are in direct contact with heme in other UPOs were aligned with those observed in experimentally determined UPOs.^26,76^ The proximal axial ligand Cys18 and the catalytic dyad Glu158/His88 were kept fixed in their modeled conformations in all design calculations to maintain the core catalytic activity. Active-site positions were chosen for design to alter the active-site cavity based on our previous work and modeling (Table S1).^26^ The designs were selected to contain 2–4 mutations each and to differ by at least two mutations from each other. 50 lowest-energy FuncLib designs (Table S2) were selected for experimental screening (see section *FuncLib design* in the SI for further details). Among the 50 designs, some positions exhibited no mutation (Leu206 and Met210) or only low sequence diversity, reflecting the high sequence conservation and energetic sensitivity of active-site positions, whereas others showed radical mutations with position Gly157 mutated in 88% of the designs.

### Designs Generate Chemically Challenging Oxyfunctionalized Terpenoids

We selected ten small cyclic and noncyclic terpenes and terpenoids (Scheme 1) to assess the catalytic capabilities of the designed *Mth*UPO variants. Additionally, we chose three standard colorimetric substrates that serve as indicators for peroxygenase or peroxidase activity (ABTS (2,2′-azino-bis­(3-ethylbenzothiazoline-6-sulfonic acid), DMP (2,6-dimethoxyphenol), and NBD (5-nitro-1,3-benzodioxole) and performed a splitGFP assay to determine the protein secretion levels independently of activity.^45^

**Scheme 1:**
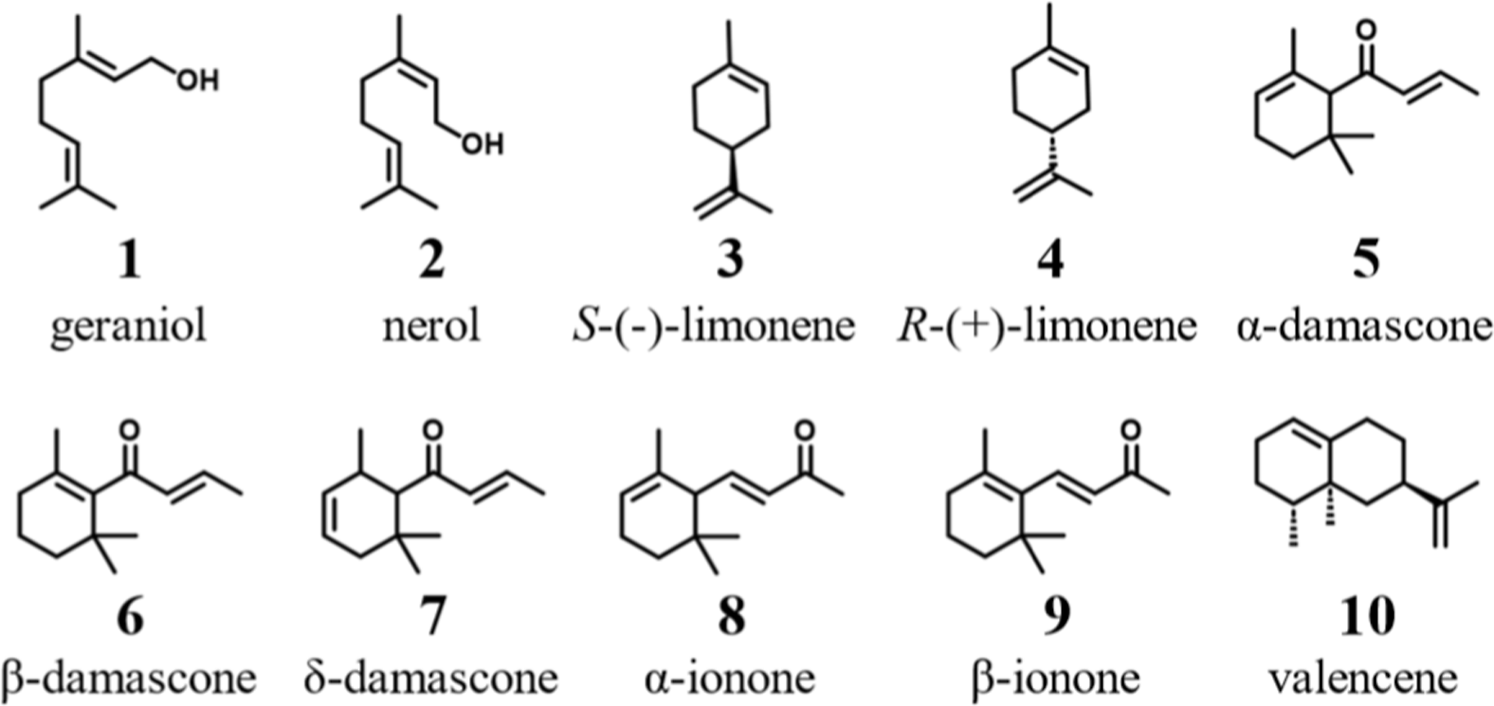
Terpenoid Substrates Investigated in This Study

Among the substrates, the oxyfunctionalized products of geraniol and nerol are of outstanding interest, as partial oxidation of their terminal alcohol to aldehyde produces citral A (geranial, **33**) and citral B (neral, **31**), which are commonly used aroma compounds in perfumery due to their strong lemon (citrus) scents.^77^ Moreover, citral is a key building block for the synthesis of vitamin A^78^ and shows anti-inflammatory, antitumor, and antibacterial activity.^79–81^ Limonene and its oxyfunctionalized products are widely used in the fragrance^82,83^ and polymer^84,85^ industry and have important pharmacological effects.^86–89^ Wild-type *Mth*UPO, however, yields a diverse array of products from limonene (Figure S3), including multiple oxyfunctionalizations, similar to the results observed with PaDa-I and artUPO.^76^ Additionally, the volatility of limonene renders upscaling reactions for product isolation and identification challenging.

Wild-type *Mth*UPO is readily secreted in functional form from yeast, and remarkably, all 50 designs were successfully expressed and secreted. Secretion levels were between 0.6-fold and 1.9-fold of wild-type levels (Figure 2), indicating that active-site design using FuncLib preserves the expressibility of the parental enzyme. Furthermore, among the designed enzymes, we measured improvements in activity relative to the wild type for nine of the ten terpenes, with only valencene (**10**), the only substrate comprising two conjugated rings in our set, not showing detectable activity in the wild type or any of the designs. The greatest improvement was seen in designs 3 and 47 relative to ABTS (1,880-fold and 1,950-fold increase in absorbance related to product formation, respectively). Design 34 demonstrated a 200-fold activity improvement over DMP (Figure 2). Both design 3 and 47 performed excellently as well for DMP, but underperformed with all other substrates, indicating that those designs improved the peroxidase rather than peroxygenase activity. By contrast, designs 22 and 25 showed 2-fold improvement on the peroxygenase model substrate NBD.

**Figure 2:**
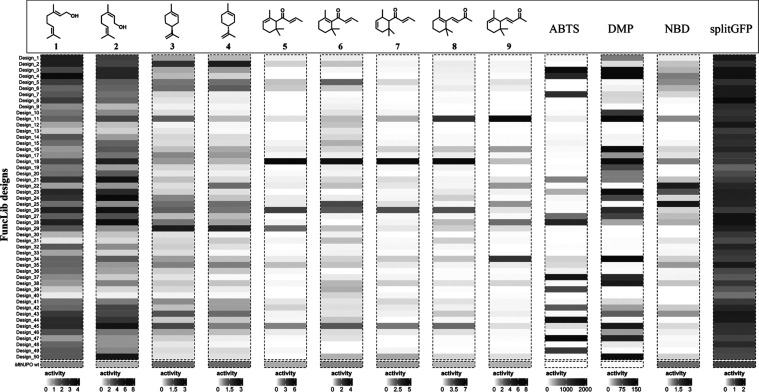
Total activity (sum of all products when multiple products were formed) relative to the activity of wild-type *Mth*UPO. The activity of wild-type *Mth*UPO is set to 1 separately for each substrate. All measurements were performed in a single replicate in microtiter plates. ABTS, DMP, and NBD were analyzed photometrically, while product formation for all other substrates was determined by GC–MS.

All FuncLib designs showed at least baseline activity toward geraniol (**1**) and nerol (**2**), confirming that all designs were functionally secreted. The largest improvement toward geraniol was seen with design 4 exhibiting a 3.8-fold increase compared to the wild type. The highest activity for nerol (**2**) is seen by design 24 with 6.2-fold improvement. The highest overall activity toward all damascones and α-ionone (**8**) is seen in design 18 with 3.1- to 6.7-fold increases. The highest activity on β-ionone (**9**) was revealed by design 11 with a 7-fold increase relative to the wild type. For limonene, the activity gain reached 1.7- and 2.2-fold with designs 2 and 29, respectively, for (*R*)-(+)-limonene (**4**) and (*S*)-(−)-limonene (**3**). Thus, a variety of different designs exhibited excellent levels of oxyfunctionalization of different substrates.

### Novel Products and Outstanding Improvements in Regioselectivity

Aside from the increase in activity, we also observed dramatic shifts in regioselectivity among the designs (Figure 3). Design 45 displays a notable shift in the formation of 3-hydroxy-β-damascone (**15**), which increased from 3% for wild-type *Mth*UPO to 46%. This regioselectivity shift is remarkable as the aliphatic C3-position is substantially less activated compared to the allylic C4-position of the main product 4-hydroxy-β-damascone (**14**). Thus, the designs can substantially change the profile of the resulting products toward ones that are less favored for chemical reactivity.

**Figure 3:**
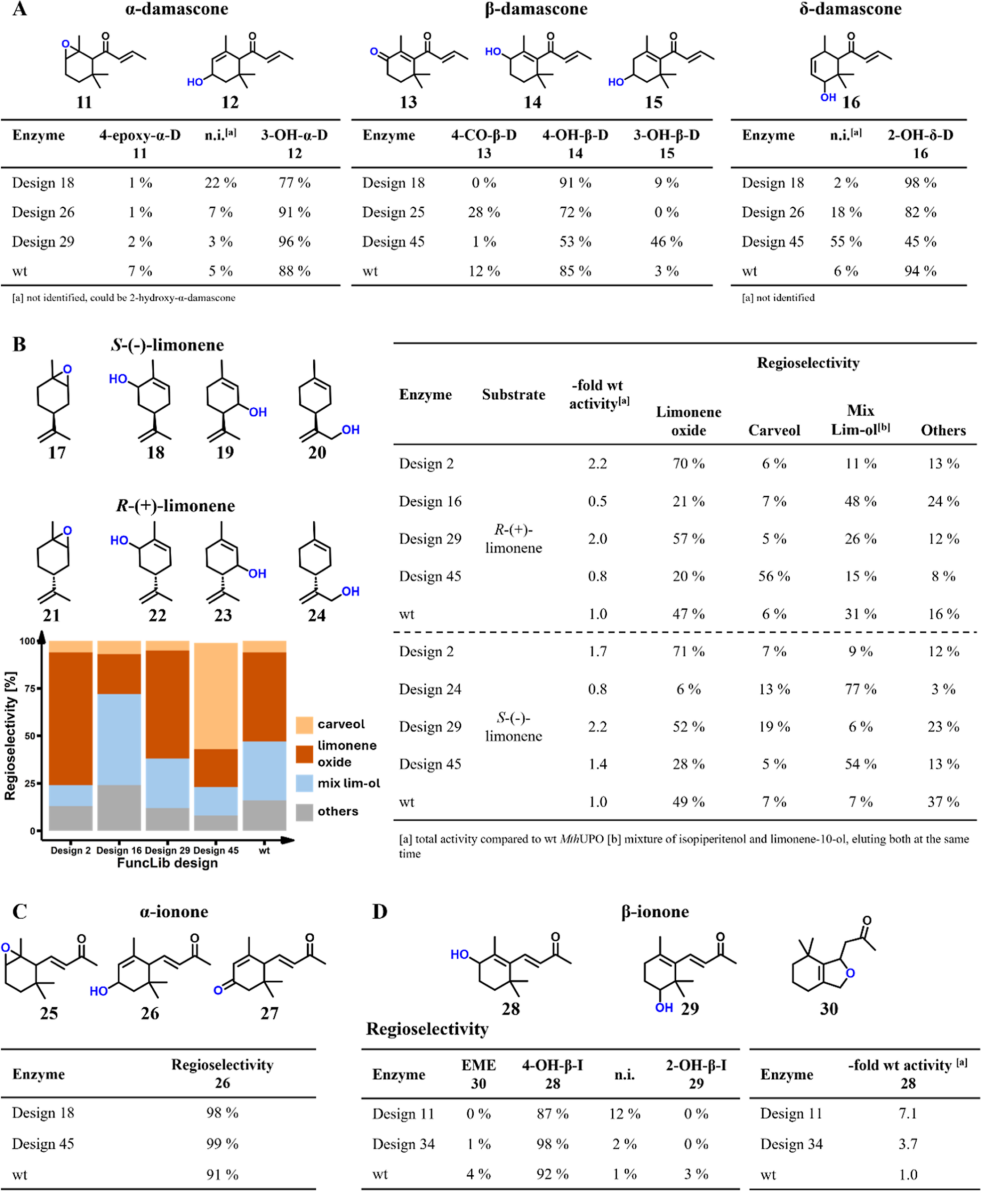
Relative abundance of selected products after conversion with different FuncLib designs. All data are analyzed by GC–MS. (A) Conversion of α-, β-, and δ-damascone. (B) Conversion of (*S*)-limonene and (*R*)-limonene stacked bar chart displays the relative abundance of products after conversion of (*R*)-limonene leading to carveol (**22**) (orange), limonene oxide (**21**) (red), mix lim-ol (**23/24**) (blue), and others (gray). Isopiperitenol elutes on the GC–MS simultaneously with a second limonene alcohol (presumably limonene-10-ol), elution peaks are not separable, but mass spectra indicate the presence of two different moieties, the sum of both products is referred to as *mix lim-ol*. (C) Conversion of α-ionone. (D) Conversion of β-ionone reveals differing relative product abundance and increased enzyme activity compared to wild-type *Mth*UPO.

We also saw shifts in chemo- and regioselectivity for limonene. For instance, wild-type *Mth*UPO mainly generates the epoxide limonene oxide (**21**), whereas design 45 mainly produces (+)-carveol (**22**) (Figure 3B) from (*R*)-(+)-limonene, alongside a 5.6-fold increase in turnover number (TON). This change in chemoselectivity is noteworthy, as hydroxylation reactions are energetically less favored than epoxidation reactions. Moreover, this reaction provides access to a valuable fragrance and pharmacologically active compound.^86,87^ The main product of (*S*)-(−)-limonene with design 45 was identified as isopiperitenol (**19**), which could only be found in trace amounts after conversion by wild-type *Mth*UPO. This activity is boosted 25-fold, reaching 310 TONs.

α- and β-Ionones are mainly converted by *Mth*UPO to 3-hydroxy-α-ionone (**26**) and 4-hydroxy-β-ionone (**28**), respectively.^26^ With the FuncLib designs, the main products remained **26** and **28**, respectively, but the regioselectivity increased strongly. For the formation of **26**, it increased from 91% (wild-type *Mth*UPO) to 98% (design 18) and 99% (design 45), respectively. For **28**, the improvement was from 92% (wild-type *Mth*UPO) to 98% (design 34).

Overall, our experiments revealed large shifts in the main substrates, the formation of novel products, and an increase from satisfactory to outstanding regioselectivities, thus underscoring the efficacy of FuncLib in generating useful functional diversity. In addition, some FuncLib designs significantly enhanced reactivities and produced regioisomers that are energetically disfavored, demonstrating how changes to the active-site geometry and electrostatics can dramatically impact the outcome of the reactions (see below).

### Large Chemoselectivity Shifts among FuncLib Designs

The FuncLib-enabled mutations lead to large shifts in chemoselectivity, as demonstrated by geraniol (**1**) and nerol (**2**). Design 4 displays excellent selectivity (>99%) for citral A (geranial, **33**, Figure 4) and a 4.5-fold improvement in TON compared to wild-type *Mth*UPO, which only showed 40% chemoselectivity for converting the terminal alcohol group. The most pronounced chemoselectivity shift toward citral B (neral, **31**) was achieved with design 26 with 89% relative abundance reaching 11,170 TONs, which corresponds to a 4.5-fold activity gain, but only a minor chemoselectivity shift compared to the 72% of wild-type *Mth*UPO. Further products are 2,3-epoxides, with a relative abundance of up to 58% for 2,3-epoxy geraniol (**34**) with design 2 and 69% for 2,3-epoxy nerol (**32**) with design 24. Some designs further exhibited overoxidation from nerol to neric acid, a reaction that was not observed with geraniol (Figure 4B). The selectively oxidized aldehydes citral A and citral B are of great importance for industrial applications, which renders design 4 with its outstanding chemoselectivity an exciting biocatalyst. Upscaling reactions with design 28 were successfully performed and yielded more than 150 mg of 2,3-epoxy nerol, which was subsequently used to determine TONs of both nerol and geraniol 2,3 oxides. Design 2 displayed a 1.8-fold increase in TON for **34** when using geraniol. Design 28 displayed a 14.3-fold boost for **32** compared to the wild-type *Mth*UPO enzyme, resulting in 9,470 TONs for product **32** (Figure 4C).

**Figure 4:**
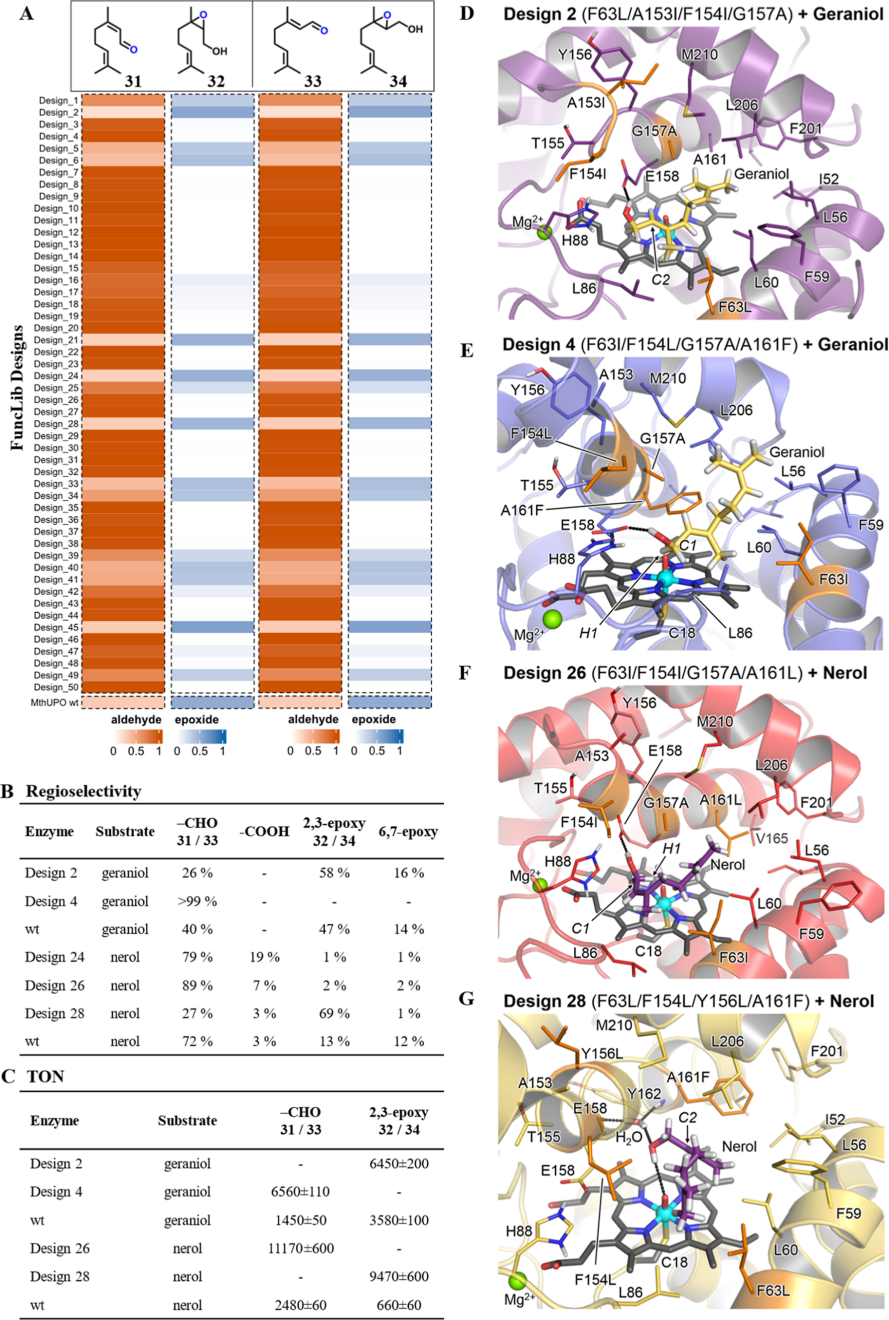
Oxyfunctionalization of geraniol (**1**) and nerol (**2**) with FuncLib designs of *Mth*UPO. (A) Proportion of 2,3-epoxide (**32/34**) (blue) and aldehyde (**31/33**) (orange) formation in the overall reaction. Measurements were performed in single replicates with enzyme from the microtiter plate supernatant. (B) Regioselectivity of selected enzyme variants toward citral A (**33**)/citral B (**31**), neric acid and 2,3-epoxy (**32/34**), and 6,7-epoxy formation measurements was performed in triplicates. (C) Turnover number of selected products; data are mean ± s.d. of measurements in triplicates. (D–G) Most representative structure of the binding mode of geraniol/nerol in different FuncLib designs as determined by clustering analysis.

### Molecular Basis of Chemoselectivity Changes

To shed light on the molecular basis for the chemoselectivity changes observed on the noncyclic terpene substrates, we conducted computational modeling based on Density Functional Theory (DFT) calculations using truncated models and molecular dynamics (MD) simulations. DFT calculations are used to elucidate intrinsic reactivity trends and provide descriptions of optimal geometries for the competing C–H activation and epoxidation transition states, while MD simulations offer structural and molecular insights into key substrate–enzyme interactions that ultimately govern the selectivities observed across selected variants. Additionally, the AI-based structure predictor Chai-1 was used to explore catalytically relevant substrate binding modes and to assess its predictive power.

For both geraniol and nerol, DFT calculations in the absence of enzyme show that allylic hydroxylation on the terminal carbon atom (C1) and epoxidation at the 2,3 double bond are the energetically most favored oxyfunctionalizations, with the terminal allylic C–H activation (via Hydrogen Atom Transfer, HAT) slightly preferred (Figures S6 and S7 in the Supporting Information). This is consistent with the major products detected experimentally for these substrates with all variants.

We chose to conduct Molecular Dynamics (MD) simulations (see Supporting Information for details) to analyze the large selectivity shifts seen in design 4 vs design 2 for geraniol oxidation and design 28 vs 26 for nerol. According to these simulations, geraniol establishes a persistent polar interaction with the Glu158 side chain of design 4, thus positioning both C1–H and C2–C3 bonds near the catalytically active oxo-ferryl cation radical complex species (compound I, see Figures 4E and S8 in the Supporting Information). The presence of the bulky Phe161 (mutated from wild-type Ala) side chain prevents the substrate from approaching the catalytic species in alternative near-attack conformations. Considering that terminal allylic hydroxylation is energetically slightly preferred, the citral A product is preferentially formed from this binding mode. On the other hand, design 2 led mainly to 2,3-epoxy geraniol with high activity and moderate chemoselectivity (1.8-fold increase and 58% selectivity). The models show that design 2 has a less dense active site, and the C2–C3 double bond of geraniol may approach the catalytic iron-oxo group. At the same time, C1–H bonds are not geometrically well-oriented for effective HAT (Figures 4D and S9 in the Supporting Information), leading to effective epoxidation by subtle substrate repositioning in the active site as compared to design 4. Therefore, MD simulations describe that active-site reshaping due to mutations repositions the substrate in the active-site cavity, which is finally responsible to switch the selectivity observed for design 4 vs design 2.

A similar scenario is found for nerol and designs 26 and 28. In design 26, nerol is preferentially bound by placing both the allylic terminal position and the C2–C3 double bond geometrically preorganized for oxyfunctionalization (Figures 4F and S10 in the Supporting Information). Nevertheless, mutations included in design 28, particularly Tyr156Leu, induce structural changes in the active-site cavity that favor the positioning of a water molecule that persistently interacts with the Glu158 and Tyr162 backbones. These structural changes, stemming from cooperation among several mutations, reposition nerol in the active site placing the C2–C3 double bond in a near-attack conformation for epoxidation by compound I while geometrically disfavoring terminal C–H activation (Figures 4G and S11 in the Supporting Information).

Recently developed AI-based structure predictors, such as AlphaFold3,^90^ can accurately predict biomolecular interactions between proteins, cofactors, and ligands. We asked whether the open-access predictor Chai-1 ^91^ could be used to shed light on the promiscuity and specificity of some of the designed enzymes modeling selected design/substrate pairs (Figure 5). We focused on design 18, which exhibits high activity toward substrates 5–8 (Figure 2), and generated ab initio models for the design, substrates, heme, and compound I oxygen. The models show that the substrates may all orient similarly, with the compound I oxygen atom placed close to the specific carbon atom that undergoes oxidation (Figures 5A and S14). In addition, we asked why design 18 oxidized substrate 7 much more efficiently than design 11. Modeling suggests that three mutated positions close to the compound I oxygen determine this specificity profile: in design 18, Phe60, Ile154, and Leu161 provide sufficient room for the ring to come close to the reactive oxygen, whereas Leu60, Leu154, and Phe161 in design 11 prevent this close approach (Figure 5B). Therefore, the Chai-1 structure predictive tool is able to capture and describe steric modifications in the active-site cavity due to mutations that impact substrate catalytically relevant binding poses.

**Figure 5:**
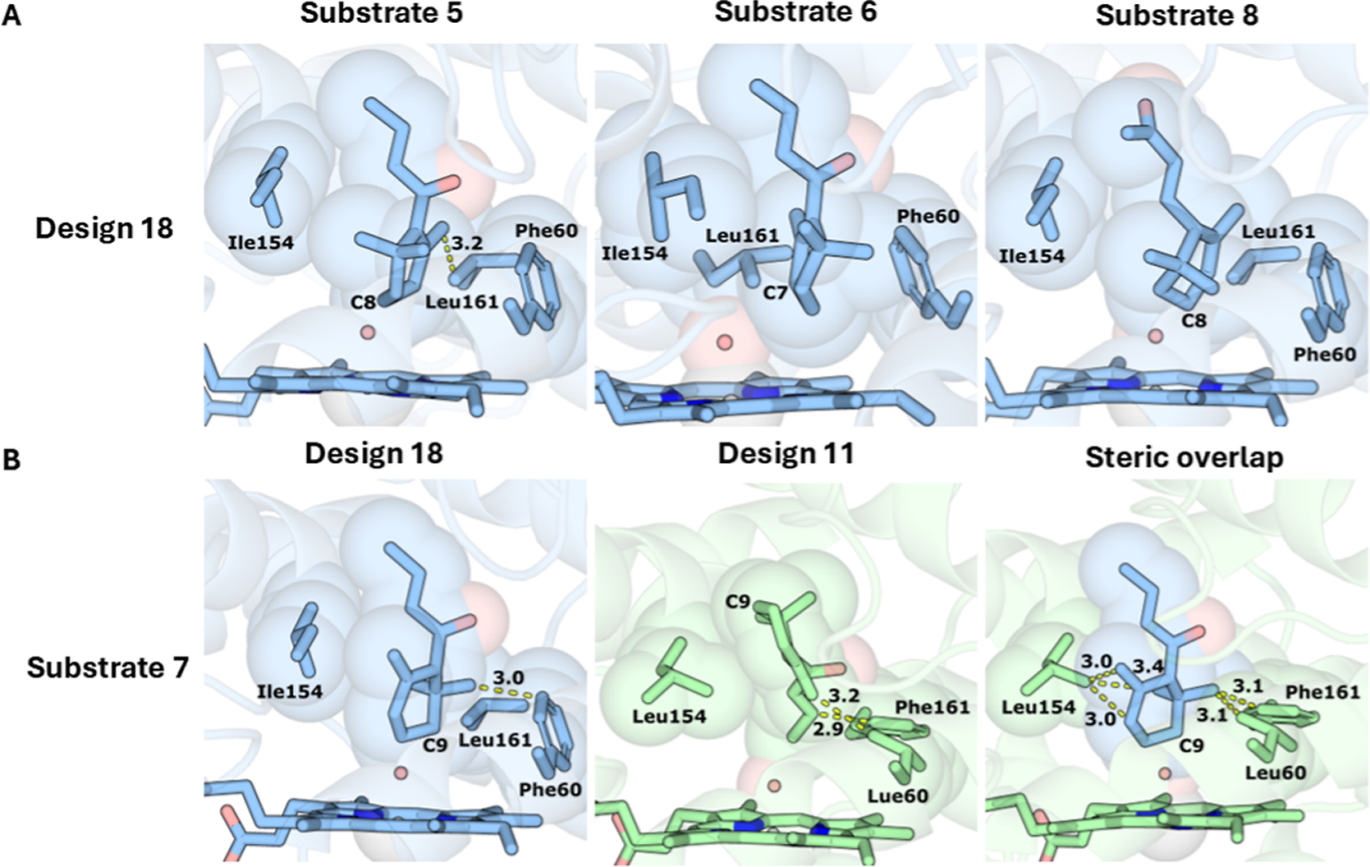
AI-based docking provides a molecular basis for the observed reactivities in design/substrate pairs. (A) Design 18, substrates 5, 6, and 8, and compound I were modeled using Chai-1, generating up to 50 models for each. For each pair, we selected a model that formed a near-attack conformation (NAC) defined as exhibiting distance <4.5 Å between the oxidized carbon and the compound I oxygen. (B) Substrate 7 exhibits much higher activity with design 18 than with design 11. In both cases, 10 models were generated with Chai-1. (left) The modeled interaction places the oxidized C9 atom of substrate 7 in a NAC. (center) Design 11 does not accommodate the NAC with this substrate, and (right) superimposing the substrate conformation modeled in design 18 in the active-site pocket of design 11 reveals significant steric overlap with designed amino acid residues that are close to the heme cofactor. Carbons in blue or green; compound I oxygen in red sphere; heme in sticks; steric overlap and interatomic distances (Å) indicated with dashed yellow lines.

### FuncLib Designs Impact Stereoselectivity of Limonene Oxide and 4-Hydroxy-β-ionone

The major influence of the FuncLib library on stereoselectivity is shown by shifts in the diastereomeric ratio of 1,2-epoxy limonene (Figure 6A) ranging from 86:14 (design 28) for *trans*-(*R*)-limonene oxide to 23:77 (design 43) for *cis*-(*R*)-limonene oxide and 94:6 (design 28 and 34) for *trans*-(*S*)-limonene oxide to 10:90 (design 6) for *cis*-(*S*)-limonene oxide. We further see outstanding changes in the enantioselectivity of 4-hydroxy-β-ionone (**28**) from an enantiomeric ratio of 76:24 for wild-type *Mth*UPO to 1:99 for both design 11 and 34 producing (*S*)-4-hydroxy-β-ionone (Figure 6B). A lack of enantioselectivity of the FuncLib designs toward the (*R*)-4-hydroxyl-β-ionone product is not surprising as the key position for controlling *R*-selectivity in β-ionone hydroxylation (Leu206)^26^ was not diversified in the FuncLib library (Table S2). These shifts confirm that FuncLib not only influences regio- and chemoselectivity but can further exert major influences on stereoselectivity in accordance with previous work on small aromatic substrates.^38^

**Figure 6:**
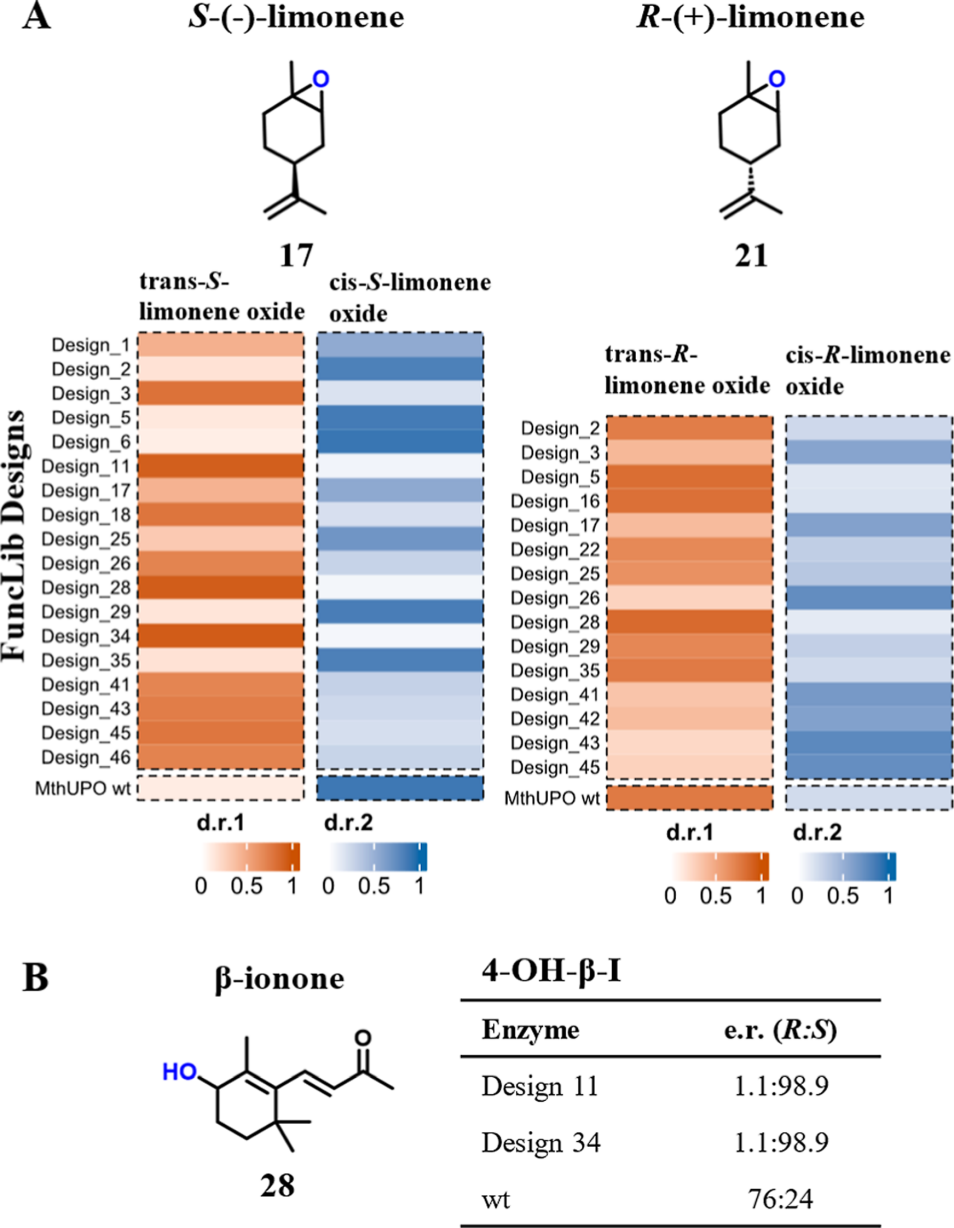
Influence of FuncLib designs on stereoselectivity. (A) Shifts in the diastereomeric ratio of *trans*-limonene oxide (orange) and *cis*-limonene oxide (blue). Measurements were performed as single replicates with enzyme from the supernatant in the microtiter plate scale. (B) Shifts in the enantiomeric ratio of 4-hydroxy-β-ionone (**28**) giving access to *S*-hydroxy-β-ionone with design 11 and design 34. Measurements are performed as single replicates.

## Discussion

In both industrial and academic contexts, the ability to produce a small set of highly active enzymes with diverse selectivities is extremely valuable to determining the feasibility of desired reactions. Previously, such diversity was generated, including in UPOs, using natural or engineered enzymes from a variety of sources. We demonstrated here that substantial diversity can also be provided by the FuncLib algorithm starting from a single enzyme that exhibits favorable stability and activity profiles.

All FuncLib designs we tested experimentally were functional and exhibited large and potentially useful changes in activity and selectivity profiles. This is remarkable, especially given that the designs were based on an AlphaFold model and that the UPO family is challenging for protein engineering due to difficulties in heterologous expression, a limited number of solved crystal structures, and its high glycosylation. For example, in a previous engineering effort in our lab, we screened 2,500 *Mth*UPO active-site variants, of which 75% displayed lower activity than the parental variant. By contrast, screening 50 designed variants in the current study led to substantial improvements in activity, chemo-, regio-, and stereoselectivity such as >99% chemo- and regioselectivity for the fragrant and pharmacological active citral A (starting from 40% for wild-type *Mth*UPO), more than 1,900-fold activity increase for ABTS, variants with at least doubled activity for all substrates, 98% regioselectivity and an inversion of enantioselectivity to an e.r. of 1:99 (starting from 76:24) for (*S*)-4-hydroxy-β-ionone, and increased regioselectivity for the formation of the energetically less favored 3-hydroxy-β-damascone (from 3% to 46%) as well as novel products and significant regioselectivity shifts with limonene. While achieving excellent selectivity may require further improvements in design methodology, FuncLib can identify substantially enhanced starting sequences for subsequent engineering campaigns, including using traditional engineering approaches, presenting a fascinating opportunity to accelerate enzyme engineering efforts toward new oxyfunctionalization selectivities.

In addition to high predictive value, the computational chemistry modeling retrospectively suggested mechanistic explanations and molecular bases for the high reactivities observed in several design variants. These calculations are highly sensitive to structural details and crucial for describing critical active-site remodeling and substrate positioning, although they require a significant computational effort. We are encouraged that the high reliability of AlphaFold and FuncLib calculations enables such sophisticated simulations, yielding valuable insights into the structural rearrangements that enable efficient catalysis. Such insights may be useful in the design of efficient and selective enzymes in other UPOs targeting different substrates. The high level of conservation of UPO structures around the heme binding site suggests that mutations in these positions may impact substrate specificity in other members of this family. We also found that the recent AI-based biomolecular modeling tools^90^ produce model structures that may help rationalize the observed specificity profiles. Our results underscore the complementary nature of MD simulations and AI-based structure prediction tools such as Chai-1. While MD provides fine-grained mechanistic insight by capturing the conformational flexibility and dynamic substrate–enzyme interactions that govern catalytic selectivity, Chai-1 offers a fast and accessible approach for identifying potential near-attack conformations. This makes it a valuable initial screening tool for exploring catalytically relevant binding modes. However, its limited resolution in accounting for subtle dynamic effects, such as those influencing enantioselectivity, highlights the need to integrate both approaches for a more comprehensive understanding of enzyme function. Importantly, our benchmarking of Chai-1 is intended as a preliminary test of its applicability in this context. A more systematic evaluation of its predictive power across broader data sets and different enzymatic systems will be a valuable direction for future work. Regardless, this is an encouraging sign that in the near future, we may be able to focus design calculations on specific desired substrates using a combination of atomistic design and AI-based substrate docking calculations and MD simulations.

Notwithstanding the high success rate of the FuncLib design library, for some substrates, we found a limited improvement in catalytic efficiency. We note that FuncLib usually designs thousands of active-site variants, but cost and time considerations preclude screening such large sets. We recently demonstrated an economical approach to design large combinatorial libraries for screening (htFuncLib).^92^ We envision that such an approach, in combination with mechanistic insights from computational modeling, may enable finding many more high-efficiency and specificity designs, including against substrates for which we did not find high-efficiency designs.

Recent progress in AI-based modeling and evolution-guided atomistic design provides unanticipated opportunities to address protein-engineering challenges at the forefront of biocatalysis that have frustrated conventional in vitro evolution approaches. The new approaches could be further refined by considering additional mechanistic insights from atomistic models obtained from computational chemistry calculations. In this study, we demonstrated that this combination effectively samples the functional space of an enzyme active site toward diverse regio- and enantioselectivity outcomes while accounting for high activities. Using this approach, in principle, any natural enzyme can be engineered quickly and effectively to tune its activity profile for basic or applied needs. These findings underscore the potential of this strategy to contribute meaningfully to future developments in green and medicinal chemistry.

## Supplementary Material



## Data Availability

Raw data for all activities found in the screening process and the sequences of the 50 FuncLib designs can be found on the following dataverse server: Muench, Judith, 2024, “UPO FuncLib Paper”, https://doi.org/10.7910/DVN/ZPKEAI, Harvard Dataverse, DRAFT VERSION

## Notes

Material and methods; gene and protein sequence; table containing mutations for all FuncLib designs; GC–MS temperature programs; utilized linker and tags; and structural characterization data for selected substrates and variants (PDF)

## Abbreviations

HAT: hydrogen atom transfer
Mix-10-ol: mixture of limonene-10-ol and isopiperitenol
TON: turnover number
UPO: unspecific peroxygenase
