# Supplementary material for: Computationally Designed Peroxygenases That Exhibit Diverse and Selective Terpene Oxyfunctionalization
Source: ACS Catal. 2025 Jul 14;15(15):12741–55. doi: 10.1021/acscatal.5c02412 (PMC12322917; doi:10.1021/acscatal.5c02412)
Supplement: Supplementary file 1 [file cs5c02412_si_001.pdf]

Supplemental Information

Computationally designed peroxygenases that exhibit diverse and selective terpene oxyfunctionalization

*Judith Münch[1], Jordi Soler[2], Ofir Gildor-Cristal[3], Sarel J. Fleishman**[3], Marc Garcia-Borràs***[2], Martin J. Weissenborn*[1]*

[1] Institute of Chemistry, Martin Luther-University Halle-Wittenberg, Weinbergweg 22, 06120, Halle (Saale), Germany

[2]Institut de Química Computacional i Catàlisi and Departament de Química, Universitat de Girona, Carrer Maria Aurèlia Capmany 69, Girona 17003, Catalonia, Spain

[3] Department of Biomolecular Sciences, Weizmann Institute of Science, Rehovot 7600001, Israel

*Martin J. Weissenborn

martin.weissenbornq@chemie.uni-halle.de

**Sarel J. Fleishman

sarel@weizmann.ac.il

Marc Garcia-Borràs

marc.garcia@udg.edu.de

I Further Material and Methods

Chemicals. Solvents were used as provided without further purification from Carl Roth (Karlsruhe, DE) as GC ultragrade. The commercially available compounds were also used without further purification from the following suppliers:

Sigma-Aldrich, St. Louis, US: ethanol (absolute), (*S*)-(-)-perillaldehyde, *trans*-(+)-limonene, (*S*)-(-)-limonene (>96 %), (-)-carveol (mixture of isomeres) (>97 %), δ-damascone (>90 %), (-)-limonene oxide (mixture of *cis* and *trans*) (99 %), β-damascone (≥90 %), (+)-valencene (>70 %), β-ionone (96 %), hydrogen peroxide solution (30 % (*w/w*) in H_2O, naphthalene (99 %), NBD (98 %), ABTS, DMP

Carl Roth, Karlsruhe, DE: acetone (Rotisolv ≥99.9 % GC Ultra Grade), acetic acid ethyl ester (Rotisolv ≥99.9 % GC Ultra Grade),

Fluka, Buchs, CH: (-)-Carvone (>99 %), (-)-perillyl alcohol

TCI chemicals, Tokyo, Japan: 4-octanone (>98 %), citral (*cis* and *trans* mixture) (>96 %), (+)-limonene (>95 %), α-ionone (>90 %), nerol (>98 %), geraniol (>96 %)

For cultivation of *S. cerevisiae* cells D-Galactose, Peptone and Synthetic Complete Mixture (Kaiser) Drop-Out (-URA) were purchased from Formedium (Hunstanton, GB). Yeast nitrogen base (without amino acids) and Yeast extract were purchased from Carl Roth (Karlsruhe, DE). BsaI was purchased from New England Biolabs (Ipswich, US). BbsI and FastDigest AscI were purchased from ThermoFisherScientific (Waltham, US) and T4 DNA Ligase from Promega (Madison, US).

Further FuncLib designs for comparison with previous work[1]

The starting structure for FuncLib design is derived from the most populated cluster of the parent F63I holoenzyme, determined by MD simulations. Key substrate-interacting

positions (L56, L60, F63, L86, F154, A161, F206, and M210) were selected based on previous work for mutation to reduced alphabet A, V, I, L, and F (M allowed at position 210). The C18 axial ligand remained fixed, with one to two mutations per design. The heme cofactor and the structural Mg ion are kept on the FuncLib model. In total, 509 variants were generated in the first round with 267 variants with lower Rosetta scores than the parent enzyme.

The second round of the FuncLib evolution was set up again using the most populated cluster structure of the previous hit 12 (L60F/F63I/A161V) as a parent holoenzyme and the same parameters as the ones used before. In total 197 variants were generated in the second round with 65 variants with lower Rosetta scores than the parent enzyme.

Calibration curves.

In every case, each sample was prepared in triplicates. The samples were extracted with 0.5 mL EtOAc containing an internal standard by vortexing for 30 seconds and centrifuged at 3000 rpm for 1 min to separate the aqueous and the organic phase. The organic phase was transferred to a GC vial with glass insert and analyzed via GC-MS.

Citral A and B

The product was available as mix of both isomers citral A and B, in a first step the ratio of both products and thus the purity of each isomer was determined. Afterwards, five stock solutions (15 mM, 10 mM, 5 mM, 2 mM, and 1 mM) in acetone were prepared. In each case 0.025 mL were added to 0.475 mL of buffered solution (0.1 M Kpi, pH 7) with final concentrations of 0.75 mM, 0.5 mM, 0.2 mM, 0.1 mM, and 0.05 mM. 0.5 mM β-ionone was used as internal standard.

2,3-Epoxy geraniol and nerol

Four stock solutions (15 mM, 10 mM, 5 mM, and 2 mM) in acetone were prepared. In each case 0.025 mL were added to 0.475 mL of buffered solution (0.1 M Kpi, pH 7) with

final concentrations of 0.75 mM, 0.5 mM, 0.2 mM, and 0.1 mM. 0.5 mM β-ionone was used as internal standard.

Carveol

Four stock solutions (10 mM, 5 mM, 2 mM, and 1 mM) in acetone were prepared. In each case 0.025 mL were added to 0.475 mL of buffered solution (0.1 M Kpi, pH 7) with final concentrations of 0.5 mM, 0.2 mM, 0.1 mM, and 0.05 mM. 0.25 mM 4-octanone was used as internal standard. The calibration curve of carveol was used for TON determination of both carveol and mix-10-ol (consisting of isopiperitenol and another product, maybe limonene-10-ol, that elute at the same time).

Identification of products.

Identification of the different products was performed through mass spectrum comparison with the literature (**Table S4**), elution time comparison with product standards and NMR for isopiperitenol. To ensure correct epoxide identification the injection temperature was lowered to 200 °C for side product identification runs.

Gene and amino acid sequence of wild type *Mth*UPO with signal peptide from α Galactosidase (*S.cerevisiae*, underline) and TwinStrep-GFP11 tag (italic) in pAGT572_Nemo 2.0 used for enzyme expression in *S. cerevisiae*:

<u>ATGTTTGCTTTTTATTTCTTGACTGCTTGTATTTCTTTGAAAGGTGTTTTTG</u>

<u>GA</u>GCAGGTTTTGACACTTGGTCACCACCTGGACCCTATGATGTTAGGGCTCC

TTGTCCGATGTTGAATACATTGGCTAATCATGGTTTCTTACCACATGATGGC

AAAGATATTACTCGTGAACAGACAGAAAACGCCTTGTTCGAAGCATTGCAC

ATCAACAAAACCTTAGCCAGCTTTCTGTTTGACTTTGCATTAACAACGAATC

CGAAGAATACCTCGACGTTTTCACTGAACGACTTAGGCAATCACAACATTTT

GGAACATGATGCATCACTAAGTAGGGCTGATGCGTACTTTGGGAATGTTCT

ACAGTTCAATCAAACTGTCTTTGATGAGACTAAAACCTACTGGGAAGGAGA
TACTATTGATTTGAGAATGGCAGCCAAAGCTAGACTAGGTAGAATCAAGAC
ATCTCAAGCTACTAATCCAACGTATTCCATGTCGGAATTAGGAGATGCTTTC
ACATATGGGGAATCTGCTGCGTATGTGGTAGTGTTAGGTGACAAAGAGTCT
CGTACTGTCAAAAGATCCTGGGTTGAATGGTTCTTCGAACATGAGCAACTTC
CTCAACATCTTGGTTGGAAAAGACCAGCAGCATCCTTCGAAGAAGAAGATC
TGAACTCATCAATGGAGGAGATTGAGAAGTACACCAAGGAATTGGAAGGTA
GCAACTCTACAAGTGGTAGTCAAAAGCATAGAAGGAGACTTCCAAGAAGA
AGAGCTCACTTTGGCTTTTCGGGTGGTTCTGCTTGGTCACATCCACAATTTG
AAAAAGGTGGAGGTTCAGGTGGAGGT*TCGGGTGGTTCTGCTTGGTCACATCC*
ACAATTTGAAAAAGATGGTGGTTCTGGTGGTGGTTCTACTAGTCGTGATCATATGG
TTCTTCATGAATATGTTAATGCTGCT GGTATTACTTGA

MFAFYFLTACISLKGVFGAGFDTWSPPGPYDVRAPCPMLNTLANHGFLPHDG
KDITREQTENALFEALHINKTLASFLFDFALTTNPKNTSTFSLNDLGNHNILEHD
ASLSRADAYFGNVLQFNQTVFDETKTYWEGDTIDLRMAAKARLGRIKTSQATN
PTYSMSELGDAFTYGESAAYVVVLGDKESRTVKRSWVEWFFEHEQLPQHLGW
KRPAASFEEEDLNSSMEEIEKYTKELEGSNSTSGSQKHRRRLPRRRAHFGF*SGGS*
AWSHPQFEKGGGSGGGSGGSAWSHPQFEKDGGSGGGSTSRDHMVLHEYVNAAGI
T

#

II Supplementary Tables

Table S1 Explored sequence space during FuncLib creation.

Position	Sequence Space
F59	FYQ
L60	LFIQ
F63	FILQV
L86	LIMV
A153	AILQV
F154	FILMV
Y156	YIL
G157	GAS
S159	SAIYQ
A161	AFILM
L206	LA
M210	MAFI

Table S2 Targeted positions and corresponding amino acid substitutions in each FuncLib variantim

Variants	Positions[a]									
	**59**	**60**	**63**	**86**	**153**	**154**	**156**	**157**	**159**	**161**
*Mth*UPO wt	**F**	**L**	**F**	**L**	**A**	**F**	**Y**	**G**	**S**	**A**
Var 1	F	L	L	L	A	L	Y	A	S	L
Var 2	F	L	L	L	I	I	Y	A	S	A
Var 3	F	L	L	L	A	F	L	A	S	F
Var 4	F	L	I	L	A	L	Y	A	S	F
Var 5	F	L	F	I	L	F	Y	A	S	F
Var 6	F	L	L	V	A	F	L	A	S	A
Var 7	F	L	L	I	A	F	Y	A	S	F
Var 8	F	L	F	L	A	L	L	A	S	M
Var 9	F	L	F	V	A	L	Y	A	S	M
Var 10	F	L	F	I	A	I	Y	A	S	M
Var 11	F	L	F	I	A	L	L	G	S	F
Var 12	F	L	L	M	A	F	Y	A	S	M
Var 13	F	L	Q	V	A	F	Y	A	S	M
Var 14	F	L	L	I	A	F	Y	G	A	M
Var 15	F	L	L	V	L	F	Y	A	S	A
Var 16	F	L	L	I	A	F	L	G	S	F
Var 17	F	L	L	V	A	F	Y	A	S	L
Var 18	F	F	F	L	A	I	Y	A	S	L
Var 19	F	I	F	I	A	F	Y	A	S	F
Var 20	F	L	I	L	A	I	Y	S	S	M
Var 21	F	L	F	L	A	F	L	A	A	F
Var 22	F	L	F	I	A	F	L	S	S	L
Var 23	F	L	F	L	A	L	I	A	S	F
Var 24	F	L	I	L	A	F	L	A	S	M
Var 25	F	L	L	V	A	F	Y	A	A	A
Var 26	F	L	I	L	A	I	Y	A	S	L
Var 27	Y	L	L	L	A	F	Y	A	S	F
Var 28	F	L	L	L	A	L	L	G	S	F
Var 29	F	L	F	I	A	L	L	A	S	A
Var 30	F	L	F	I	A	F	L	A	S	M
Var 31	F	L	L	I	A	F	Y	A	Y	A
Var 32	F	F	F	L	A	L	Y	A	S	M
Var 33	F	F	F	I	A	F	Y	A	S	M
Var 34	F	L	L	I	A	I	Y	G	S	F
Var 35	Y	L	L	I	A	F	Y	A	S	A
Var 36	F	L	L	I	I	F	Y	S	S	A
Var 37	F	L	F	I	A	L	Y	S	S	F
Var 38	F	L	L	I	I	F	Y	G	S	F
Var 39	F	L	F	V	I	F	Y	S	S	F
Var 40	F	L	Q	I	A	F	Y	S	S	F
Var 41	F	L	F	V	L	F	Y	A	S	L
Var 42	F	L	L	L	Q	F	Y	A	S	F
Var 43	F	L	L	L	L	F	Y	A	S	L
Var 44	F	L	L	L	A	L	L	S	S	A
Var 45	F	L	I	L	A	F	L	S	S	L
Var 46	F	L	Q	I	A	F	Y	A	S	L
Var 47	F	L	L	L	A	I	Y	S	S	F
Var 48	F	L	F	L	I	L	Y	A	S	M
Var 49	F	L	L	I	A	L	Y	S	S	A
Var 50	F	L	F	L	Q	I	Y	A	S	F
Number of mutations	2	4	31	29	11	21	14	44	4	40
Diversifica-tion	4 %	8 %	62 %	58 %	22 %	42 %	28 %	88 %	8 %	80 %

[a]Positions L206 and M210 were also available for mutation, but no diversification occurred among the first 50 variants. Mutations are highlighted in different colours.

Table S3 GC-MS parameter.

Substrate	GC-MS	Split	Temperature program	Internal standard
α-damascone<br>β-damascone<br>δ-damascone	GCMS-QP2010 Ultra	20	110 °C<br>4 °C/min to 185 °C<br>50 °C/min to 300 °C<br>hold 2 min	β-ionone
α-ionone	GCMS-QP2010 Ultra	10	110 °C<br>4 °C/min to 185 °C<br>50 °C/min to 300 °C<br>hold 2 min	β-ionone
geraniol<br>nerol	GCMS-QP2010 Ultra	10	100 °C<br>4 °C/min to 175 °C<br>50 °C/min to 300 °C<br>hold 2 min	β-ionone
R-(+)-limonene<br>*S*-(-)-limonene	GCMS-QP2010 Ultra	20	70 °C<br>4 °C/min to 145 °C<br>100 °C/min to 300 °C<br>hold 2 min	4-octanone
β-ionone	GCMS-QP2010 Ultra	10	110 °C<br>4 °C/min to 185 °C<br>50 °C/min to 300 °C<br>hold 2 min	α-ionone
valencene	GCMS-QP2010 Ultra	20	110 °C<br>10 °C/min to 300 °C<br>hold 2 min	naphthalene

β-ionone<br>(chiral measurement)	GCMS-QP2010 Ultra	10	45 °C<br>2 °C/min to 80 °C<br>1 °C/min to 170 °C<br>20 °C/min to 200 °C<br>hold 10 min	-

Table S4 Chemical structures and mass fragmentation of oxyfunctionalization products from enzymatic reactions with *Mth*UPO.

Nr.	Name	Structure	GC-MS (m/z)	Identification / Literature
	4-epoxy-α-ionone<br>(4-epoxy- α-I)	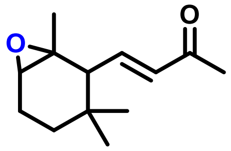	208 [M^+], 193 [$M-CH_3$]$^+$, 121 (24), 111 (37), 109 (100), 107 (24), 95 (56), 93 (30), 67 (21)	[2]
	3-hydroxy-α-ionone<br>(3-OH-α-I)	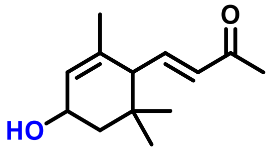	208 [M^+], 193 [$M-CH_3$]$^+$, 125 (29), 124 (25), 109 (100), 107 (23), 91 (19), 81 (29)	[2]
	3-oxo-α- ionone<br>(3-CO-α-I)	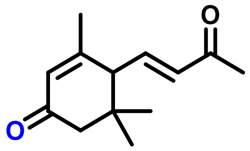	206 [M^+], 193 [$M-CH_3$]$^+$, 150 (11), 108 (100), 107 (15), 91 (8), 77 (10)	[2]

	4-hydroxy-β-ionone<br><br>(4-OH-β-I)	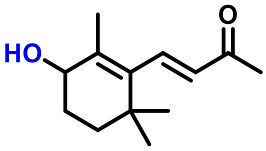	208 $[M^+]$ (28), 193 $[M-CH_3]^+$ (14), 175 (15), 165 (12), 151 (14), 137 (26), 123 (37), 109 (100), 107 (17), 95 (20), 91 (25)	1
	7,11-epoxymegastigma-5(6)-en-9-one<br><br>(EME)	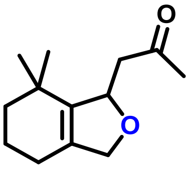	208 $[M^+]$, 193 $[M-CH_3]^+$, 151 (36) 150 (92), 135 (100), 107 (28), 95 (32), 81 (52), 79 (22)	2
	not identified product		208 [M+], 193 $[M-CH_3]^+$ (98), 175 (33), 131 (21), 109 (20), 105 (33), 91 (28), 79 (17)	
	2-hydroxy-β-ionone<br><br>(2-OH-β-I)	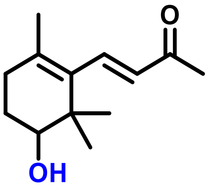	208 [M+], 193 $[M-CH_3]^+$ (67), 175 (41), 121 (42), 149 (33), 105 (37), 91 (26), 79 (21)	2
	4-epoxy-α-damascone<br><br>(4-epoxy-α-I)	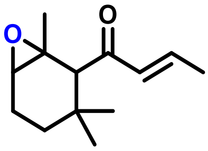	208 [M+], 193 $[M-CH_3]^+$, 137 (90), 111 (27), 109 (30), 95 (31), 81 (25), 69 (100)	2
	3-hydroxy-α-damascone<br><br>(3-OH-α-D)	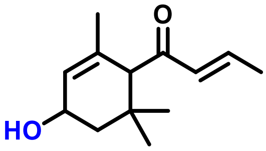	208 [M+], 193 $[M-CH_3]^+$,151 (29), 123 (22), 109 (31), 107 (73), 91 (17), 69 (100)	2
	not identified		208 [M+], 193 $[M-CH_3]^+$, 122 (33), 107 (100), 105 (12), 91 (15), 69 (51)	
	4-oxo-β-damascone<br><br>(4-CO-β-D)	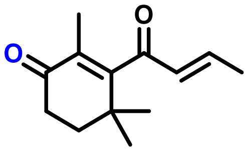	206 [M+], 191 $[M-CH_3]^+$ (18), 138 (22), 123 (15), 69 (100)	2
	4-hydroxy-β-damascone<br><br>(4-OH-β-D)	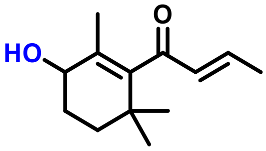	208 [M+], 193 $[M-CH_3]^+$ (23), 139 (98), 123 (25), 109 (26), 105 (26), 91 (25), 69 (100)	2
	3-hydroxy-β-damascone<br><br>(3-OH-β-D)	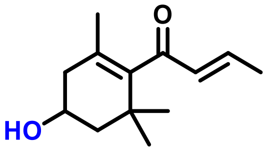	208 [M+], 193 $[M-CH_3]^+$ (42), 175 (67), 147 (28), 121 (82), 119 (33), 105 (51), 93 (30), 69 (100)	2

	2-hydroxy-δ-damascone<br><br>(2-OH-δ-D)	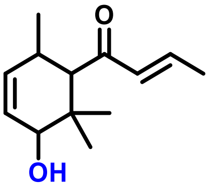	208 [M+], 193 $[M-CH_3]^+$, 124 (11), 109 (23), 107 (46), 97 (14), 69 (100)	[3], fragmentation pattern (Fig. S2)
	not identified		125 (28), 120 (33), 109 (30), 107 (54), 69 (100)	
	citral B (neral)	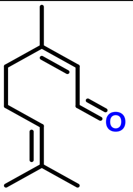	152 [M+], 137 $[M-CH_3]^+$ (9),109 (34), 94 (36), 84 (31), 69 (100), 67 (28)	product standard
	neric acid (TMS)	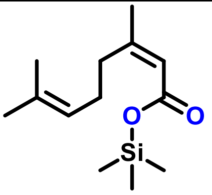	240 [M+] (2), 225 $[M-CH_3]^+$(3), 156 (34), 122 (34), 112 (30), 82 (90), 75 (56), 73 (78), 69 (100)	NIST library comparison
	2,3-epoxynerol	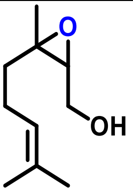	170 [M+], 155 $[M-CH_3]^+$, 109 (56), 82 (34), 69 (66), 67 (67), 41 (100)	[4]
	6,7-epoxynerol	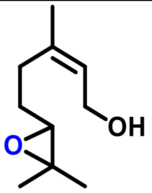	170 [M+], 155 $[M-CH_3]^+$, 85 (62), 84 (51), 81 (46), 71 (48), 59 (92), 57 (46), 41 (100)	[4]
	citral A (geranial)	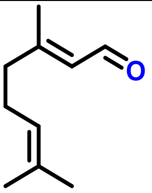	152 [M+], 137 $[M-CH_3]^+$ (16),109 (13), 94 (17), 84 (31), 69 (100), 67 (10)	product standard
	2,3-epoxygeraniol	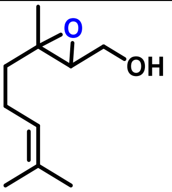	170 [M+], 155 $[M-CH_3]^+$, 109 (58), 82 (44), 69 (87), 67 (99), 41 (100)	[4]
	6,7-epoxygeraniol	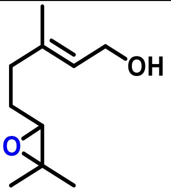	170 [M+], 155 $[M-CH_3]^+$, 85 (62), 81 (84), 71 (60), 59 (100), 57 (62), 41 (87)	[4]

	(-)-limonene oxide (1)	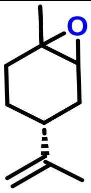	152 [M+] (1), 137 $[M-CH_3]^+$ (32), 109 (37), 93 (45), 81 (33), 79 (45), 67 (86), 43 (100)	product standard
	(-)-limonene oxide (2)	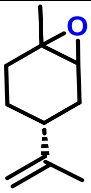	152 [M+], 137 $[M-CH_3]^+$ (11), 108 (37), 94 (52), 81 (40), 79 (52), 67 (76), 43 (100)	product standard
	(-)-isopiperitenol	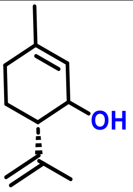	152 [M+] (1), 137 $[M-CH_3]^+$ (4), 119 (11), 91 (21), 84 (100), 79 (13), 56 (16)	NMR[5]
	(-)-carveol	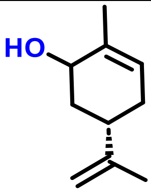	152 [M+] (2), 137 $[M-CH_3]^+$ (4), 109 (15), 94 (18), 91 (19), 84 (100), 79 (21), 69 (15), 56 (20)	product standard
	not identified (probably limonene-10-ol)	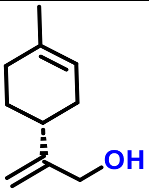	152 [M+] (2), 137 $[M-CH_3]^+$ (12), 121 (83), 105 (40), 94 (60), 93 (71), 91 (67), 84 (76), 79 (100), 67 (47)	
	not identified		152 [M+] (1), 137 $[M-CH_3]^+$ (14), 121 (58), 105 (35), 94 (59), 93 (71), 91 (57), 79 (100), 77 (37), 67 (52)	
	(+)-limonene oxide (1)	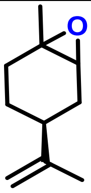	152 [M+] (2), 137 $[M-CH_3]^+$ (33), 109 (38), 93 (45), 81 (32), 79 (41), 67 (85), 43 (100)	product standard
	(+)-limonene oxide (2)	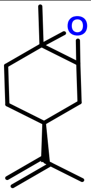	152 [M+], 137 $[M-CH_3]^+$ (5), 108 (40), 94 (62), 81 (41), 79 (50), 67 (73), 43 (100)	product standard
	(+)-isopiperitenol	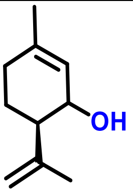	152 [M+] (1), 137 $[M-CH_3]^+$ (7), 121 (33), 91 (39), 84 (100), 79 (42), 67 (24)	NMR[5]

	not identified (probably limonene-10-ol)	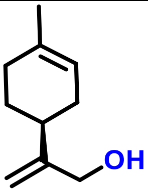	152 [M+] (1), 137 $[M-CH_3]^+$ (15), 121 (100), 105 (44), 93 (81), 91 (72), 84 (45), 79 (97), 67 (48)	
	(+)-carveol	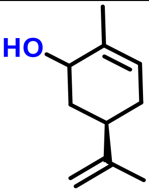	152 [M+] (2), 137 $[M-CH_3]^+$ (7), 109 (70), 91 (32), 84 (100), 79 (21), 69 (23), 56 (23)	product standard
	not identified		152 [M+] (1), 137 $[M-CH_3]^+$ (15), 121 (56), 105 (41), 94 (64), 93 (73), 91 (67), 79 (100), 77 (36), 67 (43)	

Table S5 Turnover numbers of (*R*)-(+)-limonene and (*S*)-(-)-limonene products for selected FuncLib variants.

Enzyme	Substrate	Turnover Numbers (TON)	
		**Mix Lim-ol[a]**	**Carveol**
Var 45	(*R*)-(+)-limonene	70±3	340±10
wt	(*R*)-(+)-limonene	230±11	60±5
Var 45	(*S*)-(-)-limonene	310±40	50±3
wt	(*S*)-(-)-limonene	12±2	12±1

[a] mixture of isopiperitenol and another product, maybe, limonene-10-ol, eluting both at the same time

Table S6 Regioselectivity of 3-hydroxy-α-ionone and diastereomric ratio of both 3-hydroxy-α-ionone diastereomeres for selected FuncLib variants.

Enzyme	Regioselectivity 3-OH-α-I	d.r. 3-OH-α-I
Var 18	98 %	2:98
Var 45	99 %	15:85
wt	91 %	12:88

Table S7 Energies and thermochemistry parameters of all DFT structures reported in Fig. S6.

Energies and thermochemistry parameters (at T = 298.15 K and P = 1 atm) of all DFT optimized stationary points reported in **Fig. S6**. Electronic energies (E), enthalpy (H), free energy (G), quasi harmonic corrected free energy (G-qh), electronic energies from high level single point calculations (E(SP)), and imaginary frequencies for the TS. Energies and frequencies are given in a.u. and cm^{-1}.

Cpd I + Geraniol	Electronic State	E	H	G	G-qh	E(SP)	Imag. Freq
**TS C2-O formation**	doublet (d)	-2092.676457	-2092.059380	-2092.173424	-2092.171701	-3233.342682	427.7i
	quartet (q)	-2092.675786	-2092.058498	-2092.171871	-2092.170118	-3233.342579	416.4i
**TS C1-HAT**	doublet (d)	-2092.676749	-2092.064277	-2092.179030	-2092.176207	-3233.339982	1990.5i
	quartet (q)	-2092.677116	-2092.065004	-2092.177325	-2092.175477	-3233.343119	1569.2i
**TS C4-HAT**	doublet (d)	-2092.664984	-2092.053853	-2092.167345	-2092.165676	-3233.336364	1814.3i
	quartet (q)	-2092.665536	-2092.054319	-2092.167296	-2092.165898	-3233.336749	1917.7i
**TS C5-HAT**	doublet (d)	-2092.666019	-2092.053922	-2092.166633	-2092.164939	-3233.338410	910.5i
	quartet (q)	-2092.664891	-2092.053127	-2092.166914	-2092.164988	-3233.335709	1745.1i
**TS C8-HAT**	doublet (d)	-2092.668543	-2092.055992	-2092.172137	-2092.168783	-3233.332926	908.6i
	quartet (q)	-2092.667835	-2092.056061	-2092.171090	-2092.168361	-3233.333114	1804.8i

Table S8 Energies and thermochemistry parameters of all DFT structures reported in Fig. S7.

Energies and thermochemistry parameters (at T = 298.15 K and P = 1 atm) of all DFT optimized stationary points reported in **Fig. S7**. Electronic energies (E), enthalpy (H), free energy (G), quasi harmonic corrected free energy (G-qh), electronic energies from high level single point calculations (E(SP)), and imaginary frequencies for the TS. Energies and frequencies are given in a.u. and cm^{-1}.

Cpd I + Nerol	Electronic State	E	H	G	G-qh	E(SP)	Imag. Freq
**TS C2-O formation**	doublet (d)	-2092.676947	-2092.059721	-2092.173215	-2092.171300	-3233.345299	447.3i
(conformer 1)	quartet (q)	-2092.675772	-2092.058432	-2092.170153	-2092.168889	-3233.343573	425.2i
**TS C2-O formation**	doublet (d)	-2092.684172	-2092.066749	-2092.176974	-2092.175914	-3233.351789	330.2i
(conformer 2)	quartet (q)	-2092.682501	-2092.065190	-2092.175118	-2092.174047	-3233.351443	423.1i
**TS C1-HAT**	doublet (d)	-2092.675913	-2092.063148	-2092.176817	-2092.174418	-3233.338928	1946.5i
	quartet (q)	-2092.676163	-2092.063771	-2092.176228	-2092.174314	-3233.340942	1527.9i
**TS C4-HAT**	doublet (d)	-2092.666498	-2092.054786	-2092.166546	-2092.165161	-3233.339990	1044.2i
	quartet (q)	-2092.665399	-2092.053886	-2092.164638	-2092.163646	-3233.338742	1864.3i
**TS C5-HAT**	doublet (d)	-2092.666190	-2092.054013	-2092.166757	-2092.164763	-3233.340396	946.8i
	quartet (q)	-2092.665321	-2092.053676	-2092.165825	-2092.164151	-3233.340209	1768.3i
**TS C8-HAT**	doublet (d)	-2092.664861	-2092.052733	-2092.167188	-2092.164358	-3233.329377	1480.2i
	quartet (q)	-2092.669458	-2092.057613	-2092.172305	-2092.169503	-3233.335003	1818.7i

Table S9 Cartesian coordinates (xyz, in Å) of all DFT optimized structures in **Fig. S6** and **Table S7**.

Geraniol (**1**)

O	-3.939366	-1.810334	0.258374
H	-4.888995	-2.006158	0.295011
C	-3.802368	-0.486664	-0.279572
H	-4.206891	0.259801	0.417015
H	-4.381041	-0.401889	-1.214941
C	-2.349563	-0.253304	-0.569924
H	-1.895475	-1.046633	-1.165415
C	-1.580691	0.787080	-0.210249
C	-2.059529	1.976331	0.588633
H	-1.394960	2.174071	1.438861
H	-2.046611	2.883050	-0.032123
H	-3.072938	1.855592	0.978295
C	-0.121420	0.826834	-0.624843
H	0.058440	0.098124	-1.423739
H	0.115756	1.817136	-1.041603
C	0.864158	0.532749	0.536618
H	0.673888	1.242313	1.354798
H	0.648164	-0.463421	0.935671
C	2.301162	0.661512	0.106190
H	2.583685	1.667481	-0.212557
C	3.249733	-0.287497	0.049112
C	3.053168	-1.733030	0.438382
H	3.739828	-2.011887	1.249945
H	2.036428	-1.960265	0.766733
H	3.290329	-2.394814	-0.406237
C	4.645323	0.044524	-0.426712
H	4.745293	1.100419	-0.697992
H	5.391679	-0.180829	0.348301
H	4.918514	-0.560630	-1.302816

TS C2-O formation
(d, Cpd I + Geraniol)

C	2.947069	-0.427962	-3.285195
S	2.609376	1.004223	-2.198180
H	3.486491	-1.215905	-2.752634
H	3.582260	-0.067478	-4.102155
N	2.659855	-1.104880	0.091343
N	2.275853	1.657467	0.726731
N	0.277840	-1.065570	-1.457474
C	1.702366	-3.035488	-1.077522
C	4.160076	0.267952	1.470092
C	0.789951	3.588424	0.433040
C	-1.541407	0.354347	-2.312505
C	2.663676	-2.425651	-0.281062
C	3.450556	1.459762	1.409491
C	-0.142407	2.989774	-0.404785
C	-0.855076	-0.848995	-2.209665
O	0.420124	-0.112194	1.165252
C	3.823757	-3.092932	0.261487
C	3.845199	2.676857	2.078815
C	-1.273334	3.675924	-0.984653
C	-1.249783	-2.066919	-2.871755
C	4.519103	-2.158749	0.966407
C	2.892298	3.607503	1.797885
C	-1.921174	2.772268	-1.769831
C	-0.348580	-3.023545	-2.511338
C	3.782773	-0.921086	0.860719
C	1.915404	2.962192	0.952731
C	-1.190112	1.531443	-1.664125
C	0.604703	-2.391561	-1.634450
N	-0.113603	1.684943	-0.828382
Fe	1.209397	0.256806	-0.276425
H	1.831001	-4.089490	-1.302403
H	5.076719	0.261867	2.051277
H	0.636773	4.632531	0.686891
H	-2.421690	0.374423	-2.947039
C	-0.282240	-2.402762	2.430832
O	-1.021049	-3.402633	3.148645
C	-1.186345	-1.452316	1.681431
C	-2.026003	-0.544995	2.288218
C	-1.945411	-0.223798	3.753188
C	-3.049496	0.201653	1.478896
C	-4.486675	-0.376400	1.651162
C	-5.511092	0.446887	0.918189
C	-6.286955	0.089551	-0.118805
C	-6.288021	-1.271374	-0.772800
C	-7.263597	1.071861	-0.723666
H	-1.542861	-3.902555	2.500230
H	0.424553	-2.864627	1.731650
H	0.297644	-1.869238	3.186210
H	-1.365208	-1.678174	0.633549
H	-1.516566	-1.037584	4.340895
H	-1.323119	0.672035	3.900913
H	-2.935136	0.013227	4.159885
H	-3.062853	1.256780	1.787010
H	-2.787930	0.174428	0.416288
H	-4.489755	-1.419181	1.318890
H	-4.733738	-0.389082	2.722292
H	-5.610802	1.469421	1.288153
H	-5.566296	-1.965121	-0.335456
H	-7.283338	-1.732085	-0.703521
H	-6.063864	-1.182599	-1.844870
H	-8.292994	0.690655	-0.666490
H	-7.232000	2.043538	-0.220493
H	-7.052538	1.231870	-1.790505
H	2.029483	-0.842071	-3.710744
H	5.444490	-2.276508	1.515606
H	4.058828	-4.138378	0.107738
H	4.738348	2.783492	2.681094
H	2.839133	4.639925	2.119012
H	-1.516751	4.715708	-0.807314
H	-2.810372	2.912034	-2.371108
H	-2.108106	-2.158986	-3.524949
H	-0.311716	-4.063691	-2.809215

TS C2-O formation
(q, Cpd I + Geraniol)

C	3.344879	-0.541700	-2.964278
S	2.297020	0.838353	-2.385487
H	4.136993	-0.776191	-2.249459
H	3.791775	-0.238159	-3.916581
N	2.708654	-1.056682	0.138785
N	2.273050	1.699413	0.689177
N	0.288435	-1.154716	-1.374656
C	1.757825	-3.063919	-0.922884
C	4.199578	0.385570	1.453347
C	0.784750	3.610839	0.277200
C	-1.553874	0.207075	-2.258480
C	2.719215	-2.397580	-0.174553
C	3.461039	1.556832	1.364422
C	-0.138376	2.968172	-0.542247
C	-0.864328	-0.989580	-2.102551
O	0.370123	-0.100367	1.132653
C	3.893695	-3.025608	0.380755
C	3.843863	2.811759	1.969274
C	-1.289408	3.605591	-1.136508
C	-1.264654	-2.248335	-2.687318
C	4.589134	-2.054364	1.034817
C	2.877617	3.713909	1.648121

C	-1.939380	2.649607	-1.856796
C	-0.338695	-3.171736	-2.305744
C	3.837418	-0.831474	0.887014
C	1.903351	3.015185	0.841570
C	-1.188071	1.426705	-1.701132
C	0.629852	-2.476173	-1.489822
N	-0.090988	1.647595	-0.902931
Fe	1.265019	0.271472	-0.284039
H	1.906830	-4.125753	-1.092607
H	5.124089	0.417499	2.021088
H	0.624018	4.665066	0.479693
H	-2.453877	0.188068	-2.865146
C	-0.289587	-2.297728	2.410025
O	-1.063467	-3.295620	3.091352
C	-1.160700	-1.328336	1.642396
C	-2.038308	-0.448475	2.260369
C	-1.950684	-0.127824	3.725867
C	-3.080258	0.274304	1.453484
C	-4.507711	-0.319481	1.648927
C	-5.551997	0.476990	0.914095
C	-6.338383	0.090314	-0.104247
C	-6.333965	-1.283931	-0.729959
C	-7.333559	1.049544	-0.716032
H	-1.561601	-3.788822	2.419666
H	0.434720	-2.759603	1.729924
H	0.268494	-1.775932	3.189613
H	-1.372568	-1.591350	0.610049
H	-1.238451	0.696091	3.888060
H	-2.916974	0.208210	4.117067
H	-1.615464	-0.981214	4.320254
H	-3.104813	1.333620	1.747920
H	-2.830525	0.238850	0.388414
H	-4.499814	-1.367528	1.333318
H	-4.743212	-0.319250	2.722900
H	-5.658022	1.505894	1.264359
H	-5.599992	-1.961236	-0.287269
H	-7.323626	-1.752954	-0.638564
H	-6.124037	-1.215599	-1.806449
H	-8.358109	0.659285	-0.636539
H	-7.305247	2.031972	-0.233926
H	-7.138959	1.189179	-1.788883
H	2.741353	-1.437963	-3.137305
H	5.521756	-2.139501	1.577790
H	4.137071	-4.074956	0.272761
H	4.741221	2.961217	2.556048
H	2.815054	4.761401	1.914144
H	-1.549397	4.648481	-1.007206
H	-2.846318	2.742741	-2.440446
H	-2.139944	-2.386256	-3.309334
H	-0.294229	-4.225510	-2.549993

TS C1-HAT formation

(d, Cpd I + Geraniol)

C	3.974442	-0.205218	2.314093
S	3.370507	1.080592	1.160308
H	3.253328	-0.396207	3.113460
H	4.897211	0.175836	2.765128
N	1.913191	-1.581818	0.170341
N	0.568649	0.094197	2.027847
N	2.157481	0.590949	-1.654143
C	3.074772	-1.655598	-1.997864
C	0.991587	-2.300240	2.335570
C	-0.225462	2.384660	2.428129
C	1.581151	2.947844	-2.030900
C	2.632966	-2.225366	-0.811380
C	0.492760	-1.071355	2.748291
C	0.151985	2.941225	1.213724
C	2.148552	1.737098	-2.407935
O	-0.246386	0.009189	-0.520083
C	2.844103	-3.602993	-0.446866
C	-0.168961	-0.834831	4.009306
C	-0.095813	4.311357	0.833308
C	2.845830	1.518097	-3.653333
C	2.239664	-3.790781	0.760037
C	-0.492926	0.487204	4.040023
C	0.398583	4.466791	-0.425610
C	3.281156	0.228296	-3.638418
C	1.666928	-2.525059	1.142502
C	-0.038009	1.058776	2.794821
C	0.955296	3.193556	-0.815898
C	2.843106	-0.343130	-2.387207
N	0.795962	2.279780	0.196492
Fe	1.277159	0.312724	0.146883
H	3.631125	-2.290850	-2.679503
H	0.867325	-3.144056	3.006709
H	-0.727222	3.032793	3.139513
H	1.647073	3.771388	-2.734744
C	-1.068820	-2.242532	-1.643300
O	-0.462188	-2.311950	-2.907358
C	-2.546600	-2.155794	-1.646169
C	-3.382408	-2.463809	-0.628962
C	-2.913176	-2.987776	0.704930
C	-4.875372	-2.284466	-0.788745
C	-5.502840	-1.211725	0.147421
C	-4.976598	0.176884	-0.098682
C	-5.654422	1.270564	-0.485481
C	-7.139461	1.323824	-0.755269
C	-4.940604	2.588721	-0.681673
H	-0.875242	-1.648194	-3.485180
H	-0.661087	-1.255101	-1.089164
H	-0.655133	-3.057520	-1.043097
H	-2.981667	-1.741746	-2.557410
H	-2.995984	-2.213719	1.479500
H	-3.538631	-3.828376	1.031938
H	-1.871329	-3.317710	0.692656
H	-5.109851	-2.025021	-1.827508
H	-5.370580	-3.243053	-0.573578
H	-6.589243	-1.255459	0.022708
H	-5.304164	-1.498657	1.190397
H	-3.901953	0.288031	0.054305
H	-7.332022	1.670682	-1.780122
H	-7.641022	0.361692	-0.627419
H	-7.627339	2.048604	-0.088401
H	-5.359685	3.366022	-0.026764
H	-3.868812	2.508909	-0.473028
H	-5.062459	2.954380	-1.711234
H	4.196675	-1.142045	1.796852
H	2.979563	2.268745	-4.421755
H	3.845101	-0.304741	-4.393111
H	3.384451	-4.320505	-1.050847
H	2.184431	-4.693537	1.354761
H	-0.348214	-1.593350	4.760555
H	-0.997994	1.041643	4.820595
H	0.400041	5.353187	-1.046871
H	-0.587764	5.042595	1.461769

TS C1-HAT formation

(q, Cpd I + Geraniol)

C	4.539645	0.278563	1.264551
S	2.883515	0.648567	1.935639
H	4.598600	-0.769917	0.955312
H	5.267320	0.444719	2.064881
N	2.565245	0.491502	-1.211188
N	1.682155	-1.651503	0.468557
N	0.788293	2.277363	0.108792
C	2.289997	2.872686	-1.739044

C 3.293684 -1.855089 -1.365797
C 0.265067 -2.204982 2.391919
C -0.827692 2.496720 1.945039
C 2.857912 1.618619 -1.937056
C 2.543522 -2.373414 -0.314485
C -0.268386 -0.942032 2.616110
C -0.101467 2.995028 0.873085
O -0.065923 -0.054330 -0.837188
C 3.817250 1.311191 -2.969708
C 2.574349 -3.751728 0.117899
C -1.230809 -0.639612 3.647347
C -0.144338 4.369238 0.428083
C 4.086441 -0.020699 -2.871901
C 1.723788 -3.848810 1.177361
C -1.542439 0.680985 3.522120
C 0.738381 4.477704 -0.600992
C 3.296691 -0.527580 -1.776202
C 1.173516 -2.530149 1.391700
C -0.770178 1.187461 2.414128
C 1.320944 3.169322 -0.791445
N 0.007207 0.190718 1.883731
Fe 1.205956 0.331972 0.261979
H 2.620000 3.678486 -2.386985
H 3.931876 -2.544385 -1.909829
H -0.054716 -3.003550 3.054041
H -1.488695 3.184002 2.463523
C -0.889299 -2.354615 -1.664732
O -0.141093 -2.537871 -2.836293
C -2.338246 -2.158166 -1.841436
C -3.323363 -2.432745 -0.955310
C -3.084697 -3.067367 0.392217
C -4.761698 -2.104950 -1.290596
C -5.430766 -1.066440 -0.345642
C -4.761339 0.281925 -0.363180
C -5.269515 1.461702 -0.756865
C -6.671281 1.674401 -1.277511
C -4.431946 2.719117 -0.699679
H -0.460289 -1.909664 -3.506095
H -0.466195 -1.289293 -1.136087
H -0.590116 -3.129414 -0.956420
H -2.620079 -1.670248 -2.776830
H -3.169467 -2.326108 1.198046
H -3.838023 -3.839982 0.593432
H -2.095026 -3.522711 0.478618
H -4.828479 -1.737421 -2.321116
H -5.355669 -3.030010 -1.237119
H -6.486083 -0.992170 -0.626376
H -5.416054 -1.465995 0.678923
H -3.727560 0.279679 -0.014423
H -6.644844 2.113082 -2.284800
H -7.261970 0.756666 -1.327648
H -7.214412 2.390199 -0.644444
H -4.909020 3.482244 -0.067926
H -3.429029 2.527365 -0.304356
H -4.325375 3.167941 -1.697576
H 4.779222 0.915829 0.410358
H -0.769079 5.136407 0.867310
H 0.990850 5.352540 -1.186478
H 4.213975 2.031220 -3.673990
H 4.752102 -0.621848 -3.478059
H 3.176916 -4.526388 -0.338834
H 1.482613 -4.720172 1.772787
H -2.231180 1.271952 4.112212
H -1.609744 -1.358052 4.363018

TS C4-HAT formation
(d, Cpd I + Geraniol)
C 4.21842 -1.761495 0.052300
S 3.193658 -0.757331 1.176804
H 3.779620 -2.747878 -0.117350
H 5.211516 -1.870154 0.499105
N 1.185336 -1.978769 -1.045246
N 0.064120 -1.602248 1.568062
N 2.008307 0.745807 -1.082587
C 2.365051 -0.870823 -2.888836
C 0.065787 -3.702335 0.291772
C -0.206064 -0.013817 3.416407
C 1.984562 2.846865 0.181657
C 1.748987 -1.970365 -2.294019
C -0.211708 -2.939307 1.422871
C 0.353193 1.097956 2.799779
C 2.299723 2.081718 -0.932096
O -0.507354 0.052951 -0.660821
C 1.630861 -3.271475 -2.909327
C -0.807389 -3.464140 2.628746
C 0.478480 2.399160 3.410147
C 2.990012 2.576405 -2.101659
C 0.983552 -4.068782 -2.014781
C -0.879305 -2.428916 3.511836
C 1.089630 3.201819 2.495532
C 3.105628 1.529337 -2.962951
C 0.707355 -3.251550 -0.856395
C -0.330414 -1.275845 2.840778
C 1.340870 2.389453 1.328638
C 2.482540 0.393160 -2.322632
N 0.880090 1.111873 1.529058
Fe 0.938662 -0.407742 0.206551
H 2.777802 -1.010750 -3.883262
H -0.231765 -4.746364 0.315500
H -0.568197 0.108339 4.432623
H 2.286160 3.889745 0.167977
C -5.177124 -1.751113 -1.699916
O -6.467099 -1.286777 -2.123728
C -4.285780 -0.628214 -1.249371
C -3.669055 -0.467223 -0.052753
C -3.768221 -1.463002 1.080894
C -2.803883 0.703906 0.212422
C -3.034472 1.987396 -0.586228
C -2.328451 3.192445 -0.022591
C -1.652221 4.145660 -0.684865
C -1.416211 4.164292 -2.176068
C -1.070576 5.330819 0.050569
H -6.327872 -0.696172 -2.881469
H -4.688790 -2.304589 -2.519538
H -5.376360 -2.469572 -0.902523
H -4.133414 0.135490 -2.011936
H -2.788980 -1.906986 1.289324
H -4.091739 -0.961876 2.002077
H -4.465417 -2.278948 0.879262
H -2.677385 0.892389 1.284619
H -1.586631 0.328418 -0.104941
H -2.764420 1.829548 -1.634952
H -4.120448 2.185943 -0.583167
H -2.430508 3.310666 1.058151
H -1.832873 3.297041 -2.693002
H -1.853883 5.066855 -2.625666
H -0.339666 4.198970 -2.392806
H -1.505389 6.272397 -0.314541
H -1.245575 5.269987 1.129360
H 0.013088 5.405130 -0.115579
H 4.330175 -1.255259 -0.913053
H 0.711955 -5.112133 -2.111662
H 2.002364 -3.523339 -3.894371
H -1.117183 -4.492280 2.765373
H -1.260825 -2.429339 4.524797
H 0.141973 2.645901 4.408958

H	1.359702	4.245908	2.586899
H	3.334530	3.594904	-2.227120
H	3.562610	1.508640	-3.944030

TS C4-HAT formation

(q, Cpd I + Geraniol)

C	3.134204	-2.989605	1.040403
S	2.980613	-1.176208	1.176332
H	2.288905	-3.485203	1.527860
H	4.052604	-3.282460	1.558671
N	0.921189	-2.066038	-1.099405
N	0.040288	-1.475331	1.556520
N	2.033752	0.531614	-1.337148
C	2.081469	-1.190180	-3.080316
C	-0.335728	-3.587799	0.372584
C	0.060331	0.232272	3.321671
C	2.461299	2.632587	-0.136404
C	1.398699	-2.179455	-2.384135
C	-0.429823	-2.759541	1.488333
C	0.753166	1.221030	2.632436
C	2.536071	1.808036	-1.249926
O	-0.490045	0.178182	-0.569609
C	1.069962	-3.475582	-2.924533
C	-1.031541	-3.138527	2.745999
C	1.097493	2.508814	3.184051
C	3.194355	2.167886	-2.485223
C	0.379061	-4.142173	-1.958859
C	-0.921542	-2.061527	3.573196
C	1.783014	3.180462	2.216905
C	3.091764	1.093744	-3.313772
C	0.285999	-3.257610	-0.823312
C	-0.250410	-1.026059	2.823550
C	1.849489	2.305832	1.070955
C	2.371522	0.074493	-2.586113
N	1.222864	1.119534	1.344935
Fe	0.994668	-0.448513	0.069094
H	2.405749	-1.421890	-4.089830
H	-0.773316	-4.578292	0.448375
H	-0.241817	0.454456	4.340345
H	2.922673	3.612297	-0.211160
C	-5.304754	-1.292845	-1.563112
O	-6.540426	-0.706315	-1.996925
C	-4.311116	-0.257988	-1.116766
C	-3.706738	-0.130534	0.093652
C	-3.933348	-1.080421	1.248205
C	-2.745033	0.957170	0.353844
C	-2.868098	2.264416	-0.434131
C	-2.068212	3.401713	0.144866
C	-1.289384	4.286841	-0.498972
C	-1.014492	4.281156	-1.983462
C	-0.616403	5.411388	0.253660
H	-6.341464	-0.141067	-2.760737
H	-4.868527	-1.897607	-2.375646
H	-5.577563	-1.980545	-0.760661
H	-4.066326	0.470718	-1.889390
H	-2.985564	-1.518486	1.578484
H	-4.347138	-0.538043	2.108416
H	-4.612675	-1.900949	1.008988
H	-2.582514	1.123706	1.424969
H	-1.564954	0.483336	0.012011
H	-2.610059	2.097206	-1.484312
H	-3.934015	2.550894	-0.429243
H	-2.185676	3.530743	1.222902
H	-1.510331	3.465501	-2.514247
H	-1.338165	5.227119	-2.440066
H	0.064559	4.196242	-2.171890
H	-0.944311	6.389994	-0.125312
H	-0.82945	5.371618	1.326535
H	0.473799	5.376060	0.122054
H	3.181813	-3.309982	-0.002977
H	-0.036599	-5.141129	-1.993334
H	1.338515	-3.811656	-3.917832
H	-1.474698	-4.105015	2.949460
H	-1.255004	-1.960184	4.598074
H	0.843682	2.836309	4.184116
H	2.207783	4.175177	2.256893
H	3.672162	3.121563	-2.670035
H	3.466216	0.978879	-4.322915

TS C5-HAT formation

(d, Cpd I + Geraniol)

C	0.829618	-3.167299	2.745891
S	1.650247	-2.766128	1.159542
H	0.869386	-2.325420	3.441941
H	1.376849	-4.009283	3.183397
N	-1.216681	-1.590941	0.737112
N	0.699258	0.143027	1.909115
N	0.501365	-1.948343	-1.524078
C	-1.691661	-3.008582	-1.216339
C	-1.505974	-0.394210	2.858632
C	2.961500	1.087616	1.676877
C	2.658928	-1.320745	-2.512950
C	-2.007207	-2.452914	0.016782
C	-0.269541	0.238421	2.882695
C	3.269723	0.553332	0.432792
C	1.437360	-1.979514	-2.527546
O	-0.151255	0.414482	-0.733711
C	-3.251454	-2.679283	0.712473
C	0.193015	1.079388	3.957476
C	4.504488	0.791882	-0.275553
C	0.982247	-2.832110	-3.600442
C	-3.209890	-1.931572	1.850343
C	1.453938	1.481339	3.631624
C	4.412184	0.125375	-1.459518
C	-0.234611	-3.316861	-3.230240
C	-1.936880	-1.252961	1.856284
C	1.764775	0.895011	2.353035
C	3.123663	-0.525742	-1.473131
C	-0.530002	-2.757849	-1.932232
N	2.447347	-0.252915	-0.311045
Fe	0.572326	-0.845749	0.177810
H	-2.421702	-3.674612	-1.664825
H	-2.173500	-0.222992	3.696972
H	3.709665	1.712302	2.154079
H	3.306305	-1.446773	-3.374802
C	-4.944764	0.869330	-1.150892
O	-5.791000	1.131816	-2.277190
C	-3.715964	1.718349	-1.286661
C	-3.105917	2.453573	-0.342429
C	-3.597084	2.593859	1.080428
C	-1.850764	3.231424	-0.686321
C	-0.596487	2.837498	0.123063
C	0.625685	3.654185	-0.037336
C	1.316582	3.921095	-1.174968
C	0.980481	3.346517	-2.527068
C	2.523738	4.821887	-1.140992
H	-6.499416	0.469234	-2.265696
H	-4.651277	-0.193962	-1.137668
H	-5.476010	1.065898	-0.209195
H	-3.275499	1.685386	-2.283616
H	-3.517346	3.636464	1.414875
H	-4.637429	2.279888	1.199021
H	-2.995884	1.990581	1.772984
H	-1.649806	3.158756	-1.758581
H	-2.034295	4.298126	-0.475228
H	-0.839843	2.731825	1.183948

H	-0.330738	1.693234	-0.214633
H	1.038579	4.058508	0.887356
H	0.498251	4.101523	-3.164552
H	1.898856	3.042985	-3.045131
H	0.326181	2.475260	-2.462297
H	3.424946	4.273650	-1.449514
H	2.409140	5.652126	-1.851858
H	2.698824	5.242723	-0.146281
H	-0.887869	-3.989909	-3.770601
H	1.539753	-3.025047	-4.508033
H	-0.211219	-3.466233	2.598694
H	5.138107	0.064535	-2.260262
H	5.321217	1.394729	0.100356
H	2.126980	2.115947	4.193786
H	-0.383116	1.313610	4.843472
H	-3.959350	-1.837096	2.625734
H	-4.042519	-3.327273	0.357249

TS C5-HAT formation

(q, Cpd I + Geraniol)

C	1.261461	-2.922474	2.881248
S	2.198391	-2.156586	1.514343
H	0.845778	-2.151657	3.537751
H	1.962762	-3.530003	3.461812
N	-0.868523	-1.762625	0.842047
N	0.717030	0.422754	1.789117
N	0.857676	-2.056374	-1.404727
C	-1.136490	-3.415281	-0.952986
C	-1.331416	-0.410728	2.846150
C	2.754770	1.718687	1.367787
C	2.932461	-1.263267	-2.450127
C	-1.525864	-2.800093	0.232908
C	-0.236760	0.445215	2.772832
C	3.170149	1.068127	0.213364
C	1.812792	-2.080784	-2.392994
O	-0.172908	0.309827	-0.763453
C	-2.708081	-3.150134	0.981970
C	0.064048	1.481129	3.734358
C	4.353977	1.418371	-0.532953
C	1.504930	-3.111304	-3.357819
C	-2.766986	-2.296917	2.043425
C	1.215435	2.078617	3.318342
C	4.409085	0.579127	-1.604431
C	0.363279	-3.717986	-2.935387
C	-1.619622	-1.428488	1.944684
C	1.616806	1.408152	2.103215
C	3.256676	-0.284153	-1.517087
C	-0.031795	-3.059865	-1.711496
N	2.521913	0.019354	-0.399165
Fe	0.769537	-0.811317	0.152841
H	-1.752566	-4.230992	-1.317897
H	-2.016593	-0.274470	3.677131
H	3.375899	2.532092	1.729531
H	3.607836	-1.397191	-3.289166
C	-5.147983	-0.095705	-0.824604
O	-5.839572	-0.430169	-2.036207
C	-3.956278	0.740224	-1.185751
C	-3.588337	1.931335	-0.685938
C	-4.361575	2.711260	0.351931
C	-2.317017	2.597473	-1.180957
C	-1.179100	2.592147	-0.138305
C	-0.155394	3.641151	-0.110236
C	0.440273	4.329053	-1.123820
C	0.189704	4.114902	-2.593898
C	1.451074	5.403608	-0.810792
H	-6.501958	-1.102590	-1.811732
H	-4.803202	-1.014644	-0.322162
H	-5.818039	0.423559	-0.126063
H	-3.320581	0.283385	-1.944679
H	-4.600166	3.713881	-0.028539
H	-5.296903	2.228987	0.644350
H	-3.768544	2.859529	1.263812
H	-1.991166	2.101547	-2.100742
H	-2.542468	3.640824	-1.447967
H	-1.558474	2.411399	0.871453
H	-0.580518	1.472924	-0.346495
H	0.208108	3.872854	0.890895
H	-0.373951	4.956446	-3.022725
H	1.145079	4.078444	-3.133708
H	-0.353646	3.195446	-2.810606
H	2.427662	5.163375	-1.254640
H	1.145070	6.365993	-1.245500
H	1.587286	5.543490	0.265837
H	-0.174976	-4.539389	-3.390795
H	2.102914	-3.329450	-4.233375
H	0.449605	-3.552840	2.511583
H	5.149008	0.537638	-2.393520
H	5.040372	2.209021	-0.258042
H	1.759656	2.892534	3.780230
H	-0.534696	1.702005	4.608773
H	-3.510325	-2.245344	2.828793
H	-3.392644	-3.944590	0.713612

TS C8-HAT formation

(d, Cpd I + Geraniol)

C	5.000364	-0.483150	1.444956
S	3.777903	0.874437	1.320029
H	4.644857	-1.291348	2.088661
H	5.908638	-0.055982	1.883155
N	2.868798	-0.387560	-1.334294
N	2.113888	-1.786251	1.033201
N	1.578660	1.965951	-0.341337
C	2.826834	1.798563	-2.453564
C	3.319627	-2.756387	-0.879108
C	0.975036	-1.587850	3.200579
C	0.178969	2.866174	1.460721
C	3.176294	0.457474	-2.372361
C	2.736540	-2.823499	0.379687
C	0.537360	-0.277953	3.065362
C	0.854619	2.968805	0.251346
O	0.397000	-0.442098	-0.601702
C	3.910880	-0.258468	-3.387910
C	2.719723	-4.005939	1.204906
C	-0.229568	0.430029	4.060200
C	0.906403	4.162029	-0.558587
C	4.034679	-1.543528	-2.954915
C	2.081486	-3.672593	2.361558
C	-0.472553	1.673532	3.560905
C	1.667900	3.865435	-1.647860
C	3.383523	-1.615781	-1.668635
C	1.696224	-2.288265	2.241480
C	0.154187	1.734143	2.263397
C	2.076459	2.488170	-1.510817
N	0.752719	0.530138	1.973203
Fe	1.766228	0.057524	0.304972
H	3.144319	2.345402	-3.335597
H	3.775986	-3.661144	-1.267694
H	0.721733	-2.109502	4.117806
H	-0.346166	3.745755	1.818609
C	-7.160631	2.447679	-0.589513
O	-8.527732	2.595036	-0.179129
C	-6.791776	1.014222	-0.857641
C	-6.360775	0.459332	-2.003269
C	-6.154562	1.213216	-3.296785
C	-6.053181	-1.025347	-2.060168
C	-4.560993	-1.377918	-2.314849

C	-3.649879	-0.920298	-1.216185
C	-2.706866	-1.636328	-0.543829
C	-2.362459	-3.078946	-0.825555
C	-1.907961	-0.979866	0.500264
H	-8.645041	2.069312	0.628512
H	-6.489822	2.864684	0.179775
H	-7.056017	3.073291	-1.478522
H	-6.900304	0.360385	0.011087
H	-5.090300	1.272585	-3.560119
H	-6.651997	0.691395	-4.125008
H	-6.539536	2.234772	-3.263359
H	-6.374174	-1.507051	-1.129246
H	-6.638030	-1.477957	-2.874010
H	-4.483364	-2.457220	-2.475493
H	-4.244401	-0.906135	-3.257981
H	-3.777699	0.121555	-0.921190
H	-2.483048	-3.687168	0.080161
H	-2.967923	-3.523057	-1.618038
H	-1.308291	-3.160092	-1.121163
H	-1.630087	-1.626188	1.339369
H	-0.816554	-0.712017	0.030277
H	-2.299705	-0.018685	0.842388
H	5.246655	-0.887196	0.459549
H	0.418673	5.095394	-0.308087
H	1.933046	4.503097	-2.481434
H	4.273749	0.182567	-4.307442
H	4.522791	-2.377316	-3.443108
H	3.148727	-4.958213	0.920364
H	1.874549	-4.294344	3.223192
H	-1.012986	2.492093	4.018868
H	-0.532511	0.012492	5.011855

TS C8-HAT formation

(q, Cpd I + Geraniol)

C	4.148078	-0.868149	2.835890
S	3.647489	0.294873	1.521036
H	3.362138	-0.948872	3.593215
H	5.047798	-0.465002	3.310644
N	2.052964	-2.390937	0.973657
N	0.791908	-0.161793	2.251527
N	2.614078	-0.793975	-1.302685
C	3.254283	-3.146154	-1.030241
C	0.899680	-2.439483	3.148937
C	0.342021	2.242314	2.052063
C	2.419212	1.461582	-2.257644
C	2.704010	-3.345262	0.230816
C	0.532368	-1.097949	3.216283
C	0.879900	2.439023	0.785803
C	2.795013	0.127282	-2.307885
O	0.156413	-0.905323	-0.271966
C	2.694228	-4.608930	0.926089
C	-0.150354	-0.486311	4.333385
C	0.866724	3.699310	0.083784
C	3.488609	-0.481270	-3.419734
C	2.010128	-4.414198	2.087789
C	-0.290061	0.834460	4.031175
C	1.439536	3.479680	-1.132763
C	3.740564	-1.771254	-3.069161
C	1.609900	-3.028596	2.111458
C	0.300279	1.029056	2.727631
C	1.807531	2.085146	-1.173792
C	3.194414	-1.958255	-1.744949
N	1.467224	1.471922	0.003242
Fe	1.684924	-0.496627	0.440739
H	3.744292	-3.991978	-1.501891
H	0.625174	-3.074124	3.985723
H	-0.090926	3.105361	2.548076
H	2.633888	2.073171	-3.128222
C	-5.310431	4.715756	-1.900542
O	-6.494754	5.506798	-1.724847
C	-5.588125	3.238789	-1.836380
C	-5.379565	2.304759	-2.779928
C	-4.798684	2.582680	-4.147194
C	-5.739534	0.855885	-2.509363
C	-4.532946	-0.123512	-2.464018
C	-3.575181	0.170226	-1.348249
C	-3.100016	-0.674787	-0.389092
C	-3.445244	-2.143593	-0.299081
C	-2.149685	-0.179519	0.603876
H	-6.867594	5.279411	-0.857890
H	-4.562000	4.984922	-1.136815
H	-4.902435	5.016572	-2.867920
H	-6.013961	2.910083	-0.885215
H	-3.801453	2.136366	-4.255976
H	-5.427990	2.133234	-4.926751
H	-4.704436	3.648946	-4.364390
H	-6.288019	0.780675	-1.563143
H	-6.416334	0.504735	-3.301983
H	-4.921699	-1.144130	-2.399338
H	-3.995838	-0.063435	-3.423222
H	-3.230555	1.203806	-1.303053
H	-3.863460	-2.380272	0.687686
H	-4.159736	-2.468921	-1.057908
H	-2.537321	-2.750394	-0.410507
H	-2.189937	-0.668552	1.580852
H	-0.958174	-0.517846	0.209011
H	-2.071689	0.906875	0.682290
H	4.357812	-1.861659	2.432997
H	3.748589	0.033697	-4.335767
H	4.248128	-2.540157	-3.637448
H	3.147183	-5.518374	0.552651
H	1.788376	-5.130080	2.868982
H	-0.466046	-1.012930	5.225021
H	-0.747247	1.617969	4.621973
H	1.604296	4.182956	-1.939101
H	0.461881	4.620282	0.483496

Table S10 Cartesian coordinates (xyz, in Å) of all DFT optimized structures in **Fig. S7** and **Table S8**

Nerol (**2**)

O	-3.721164	-1.593448	-0.854277
H	-4.069693	-2.477592	-0.657598
C	-3.178687	-1.073848	0.370253
H	-2.300118	-1.654353	0.680203
H	-3.927118	-1.160081	1.175631
C	-2.844596	0.372495	0.153983
H	-3.693824	0.972084	-0.176657
C	-1.660286	0.984028	0.316637
C	-1.526547	2.467594	0.058720
H	-0.747551	2.676381	-0.686475
H	-2.463301	2.905012	-0.300354
H	-1.229590	2.998275	0.974034
C	-0.383571	0.285902	0.744931
H	0.158499	0.928066	1.452299
H	-0.600041	-0.644006	1.280506
C	0.560148	-0.036431	-0.445139
H	0.006743	-0.673909	-1.149835
H	0.798675	0.884977	-0.985493
C	1.808381	-0.751348	-0.000843
H	1.626147	-1.728624	0.451920
C	3.084729	-0.339783	-0.073678
C	3.538667	0.977223	-0.656035
H	4.227525	0.811594	-1.496150
H	2.716578	1.600612	-1.014986
H	4.098716	1.555997	0.091597
C	4.206722	-1.210902	0.442623
H	3.836015	-2.155196	0.854105
H	4.924828	-1.445149	-0.356013
H	4.777064	-0.696079	1.228774

TS C2-O formation
(d, conformer 1, Cpd I + Nerol)

C	-3.971650	2.283952	0.182077
S	-2.999423	1.340278	1.411622
H	-3.368922	3.067161	-0.285692
H	-4.797376	2.760738	0.721831
N	-0.509567	2.155884	-0.222988
N	-0.186493	0.535374	2.112171
N	-2.121959	0.121697	-1.398673
C	-1.478536	2.237778	-2.474277
C	0.740634	2.788918	1.793812
C	-0.717446	-1.632184	3.129377
C	-3.147318	-2.081395	-1.038377
C	-0.728136	2.767119	-1.432435
C	0.531964	1.629109	2.528612
C	-1.501525	-2.138863	2.100381
C	-2.907484	-0.936203	-1.788298
O	0.378919	-0.273981	-0.374466
C	-0.081412	4.058511	-1.459960
C	1.067165	1.396407	3.849336
C	-2.182801	-3.410348	2.150384
C	-3.435701	-0.702112	-3.109772
C	0.529502	4.217473	-0.254077
C	0.669527	0.148639	4.221429
C	-2.874397	-3.532975	0.983594
C	-2.951330	0.503732	-3.519590
C	0.263373	3.021472	0.510986
C	-0.113456	-0.381064	3.130159
C	-2.613137	-2.338593	0.216964
C	-2.134153	1.012821	-2.445488
N	-1.766964	-1.506135	0.910925
Fe	-1.058287	0.278365	0.302970
H	-1.575957	2.834867	-3.375461
H	1.337980	3.570084	2.253234
H	-0.589672	-2.250821	4.011972
H	-3.798155	-2.835361	-1.469699
C	1.114759	-1.283829	-2.773790
O	1.633413	-2.121987	-3.817503
C	0.868520	-2.053254	-1.496962
C	1.862727	-2.559827	-0.688461
C	1.521828	-3.475892	0.449536
C	3.311123	-2.185500	-0.852669
C	3.771806	-1.088197	0.155722
C	5.234705	-0.774709	0.001428
C	5.806601	0.382013	-0.373501
C	5.057940	1.648087	-0.714503
C	7.309772	0.499215	-0.479453
H	0.974513	-2.813622	-3.991377
H	0.186507	-0.793845	-3.089787
H	1.865347	-0.506018	-2.622955
H	-0.134021	-2.448146	-1.360700
H	0.463587	-3.748653	0.457152
H	2.125397	-4.392814	0.402459
H	1.751912	-3.003286	1.415659
H	3.930534	-3.079267	-0.694413
H	3.510110	-1.838006	-1.869540
H	3.141949	-0.205451	0.021573
H	3.583801	-1.454144	1.175000
H	5.899905	-1.614970	0.212656
H	3.973419	1.548543	-0.630437
H	5.373075	2.470731	-0.057343
H	5.289841	1.967303	-1.740196
H	7.693030	1.283985	0.188127
H	7.813024	-0.439345	-0.225712
H	7.611929	0.785034	-1.497021
H	-4.386301	1.635693	-0.593837
H	1.112155	5.056885	0.103291
H	-0.107923	4.740713	-2.300051
H	1.668179	2.107375	4.401722
H	0.872823	-0.379224	5.144433
H	-2.134134	-4.100173	2.983237
H	-3.511630	-4.344796	0.656883
H	-4.088408	-1.383920	-3.639875
H	-3.124840	1.019014	-4.455692

TS C2-O formation
(q, conformer 1, Cpd I + Nerol)

C	-4.316532	1.625798	0.094928
S	-3.123386	1.073396	1.363359
H	-3.927746	2.463261	-0.489003
H	-5.228667	1.936605	0.614684
N	-1.439727	1.474541	-1.307303
N	-0.033934	1.862275	1.136195
N	-2.225760	-1.137477	-0.460902
C	-2.735479	-0.138804	-2.640708
C	-0.207319	3.504045	-0.679292
C	0.515570	0.854814	3.308588
C	-2.174464	-2.709406	1.424671
C	-2.110603	1.086005	-2.445140
C	0.237463	3.082057	0.565603
C	-0.181103	-0.339783	3.152697
C	-2.547917	-2.328916	0.140993
O	0.362013	-0.307919	-0.341556
C	-2.084101	2.148199	-3.421548
C	1.052112	3.877554	1.455027
C	-0.248900	-1.383953	4.147372
C	-3.337097	-3.139443	-0.757322
C	-1.387862	3.178338	-2.865722
C	1.264578	3.127503	2.569938

C	-1.013662	-2.380016	3.619666
C	-3.489256	-2.421219	-1.904727
C	-0.980879	2.745430	-1.550688
C	0.576482	1.872949	2.368375
C	-1.411308	-1.947359	2.301137
C	-2.795244	-1.169456	-1.706520
N	-0.898856	-0.698300	2.042163
Fe	-1.073562	0.358570	0.320581
H	-3.230388	-0.298665	-3.593574
H	0.082770	4.497990	-1.004904
H	1.042524	1.007183	4.245208
H	-2.505108	-3.683515	1.771578
C	1.139632	-1.141927	-2.703980
O	1.627325	-1.975239	-3.765361
C	0.880315	-1.929248	-1.438534
C	1.891526	-2.512292	-0.685830
C	1.561807	-3.494341	0.399610
C	3.343854	-2.157405	-0.860281
C	3.827089	-1.060525	0.137174
C	5.305617	-0.811018	0.015207
C	5.936633	0.311959	-0.366891
C	5.252417	1.601059	-0.753936
C	7.445856	0.363018	-0.432533
H	0.942782	-2.636964	-3.955601
H	0.224197	-0.620463	-3.004230
H	1.914097	-0.389991	-2.540964
H	-0.111001	-2.363341	-1.347047
H	0.484539	-3.639061	0.513994
H	2.025413	-4.469379	0.188312
H	1.967658	-3.166870	1.367014
H	3.951526	-3.058661	-0.698983
H	3.543324	-1.820587	-1.881134
H	3.239884	-0.153571	-0.026758
H	3.599348	-1.397955	1.158315
H	5.928334	-1.673852	0.262175
H	4.162582	1.546919	-0.703589
H	5.579499	2.422401	-0.101002
H	5.529814	1.891449	-1.776885
H	7.843990	1.145486	0.229049
H	7.900960	-0.590142	-0.144491
H	7.788328	0.612763	-1.446827
H	-4.568771	0.803337	-0.581303
H	-1.155347	4.145441	-3.292960
H	-2.544497	2.093983	-4.399900
H	1.401674	4.878494	1.236296
H	1.824170	3.383730	3.460542
H	0.231837	-1.341960	5.116457
H	-1.290217	-3.327466	4.064497
H	-3.719107	-4.124547	-0.521345
H	-4.023729	-2.692909	-2.806201

TS C2-O formation

(d, conformer 2, Cpd I + Nerol)

C	3.664136	1.898083	-1.924978
S	2.536853	0.504690	-2.289661
H	3.109367	2.818348	-1.724568
H	4.277459	2.051871	-2.819918
N	0.377253	1.986556	-0.652384
N	-0.222774	-0.561378	-1.816791
N	2.469117	0.789681	0.865393
C	1.842650	3.167061	0.924018
C	-1.376685	1.553049	-2.314911
C	0.398714	-2.933987	-1.871175
C	3.641991	-1.314433	1.344265
C	0.794471	3.111919	0.014090
C	-1.161077	0.191244	-2.481541
C	1.413625	-2.883777	-0.925218
C	3.451857	0.053693	1.485764
O	-0.107419	-0.057749	0.876578
C	-0.003529	4.248071	-0.384839
C	-1.908871	-0.643207	-3.390530
C	2.219742	-4.015699	-0.536602
C	4.250788	0.906763	2.329132
C	-0.905308	3.794187	-1.297794
C	-1.409780	-1.905067	-3.269764
C	3.128297	-3.560121	0.369444
C	3.737857	2.164297	2.219085
C	-0.661774	2.379758	-1.459059
C	-0.358252	-1.844216	-2.283799
C	2.880873	-2.148365	0.536086
C	2.626696	2.085458	1.304085
N	1.820124	-1.758640	-0.247524
Fe	1.036602	0.089554	-0.380392
H	2.073857	4.131569	1.364591
H	-2.166732	2.008009	-2.903864
H	0.188120	-3.894447	-2.330463
H	4.456266	-1.766216	1.901599
C	-0.571757	-0.505346	3.446225
O	0.728391	0.054422	3.471044
C	-0.733107	-1.485459	2.312321
C	-1.940991	-1.898749	1.778474
C	-1.997927	-3.080667	0.858119
C	-3.245006	-1.196989	2.050892
C	-3.591123	-0.131889	0.963399
C	-4.873522	0.587366	1.281307
C	-6.043342	0.544659	0.621643
C	-6.304905	-0.263066	-0.626990
C	-7.230650	1.344584	1.106271
H	0.876548	0.337956	2.546951
H	-1.340757	0.277897	3.394523
H	-0.706928	-1.033638	4.400728
H	0.137711	-2.110212	2.130045
H	-2.621287	-3.871875	1.299770
H	-2.469614	-2.816337	-0.098332
H	-1.008473	-3.492965	0.649534
H	-4.054546	-1.937103	2.075419
H	-3.235459	-0.704468	3.028690
H	-2.759271	0.581716	0.921255
H	-3.631040	-0.619267	-0.015339
H	-4.828634	1.209474	2.177730
H	-6.615180	0.392142	-1.452911
H	-5.439113	-0.838617	-0.962573
H	-7.133982	-0.965536	-0.463246
H	-7.571383	2.051494	0.336545
H	-8.085791	0.688875	1.323482
H	-6.998778	1.913692	2.012225
H	4.323241	1.680140	-1.081079
H	0.128535	5.253708	-0.006528
H	-1.670072	4.348886	-1.826287
H	-2.705628	-0.289943	-4.032550
H	-1.710734	-2.804278	-3.791930
H	2.097488	-5.018261	-0.926242
H	3.908997	-4.109694	0.879621
H	5.088138	0.568649	2.925921
H	4.068024	3.073120	2.705635

TS C2-O formation

(q, conformer 2, Cpd I + Nerol)

C	4.119854	0.101975	-1.954462
S	2.362938	0.364798	-2.383675
H	4.483850	0.855732	-1.252486
H	4.694624	0.166037	-2.884184
N	0.452306	2.133097	-0.572793
N	-0.418353	-0.295721	-1.772303
N	2.548134	0.735224	0.750293
C	2.203179	3.166769	0.805592

C	-1.476617	1.896487	-2.075504
C	0.023836	-2.710734	-1.939869
C	3.479400	-1.493155	1.225815
C	1.059625	3.214092	0.021432
C	-1.367000	0.530660	-2.315631
C	1.081963	-2.787286	-1.041859
C	3.450010	-0.111768	1.358513
O	-0.069504	-0.020283	0.915783
C	0.338985	4.425235	-0.296357
C	-2.241208	-0.221007	-3.183755
C	1.771535	-4.007090	-0.689481
C	4.384442	0.650085	2.148020
C	-0.704835	4.065450	-1.091017
C	-1.807214	-1.512503	-3.156979
C	2.727662	-3.669478	0.220195
C	4.042916	1.962343	2.011297
C	-0.621507	2.634192	-1.270308
C	-0.672012	-1.553894	-2.266704
C	2.629468	-2.240914	0.415180
C	2.890760	2.006871	1.145971
N	1.617238	-1.729290	-0.352742
Fe	0.998258	0.213339	-0.424297
H	2.579188	4.104953	1.200842
H	-2.279904	2.429662	-2.573813
H	-0.295449	-3.633573	-2.413955
H	4.246916	-2.029905	1.774402
C	-0.462482	-0.258194	3.451793
O	0.809306	0.362770	3.450326
C	-0.577352	-1.303852	2.368273
C	-1.786137	-1.853952	1.945508
C	-1.805421	-3.122924	1.148334
C	-3.116949	-1.194804	2.196816
C	-3.498097	-0.169440	1.084241
C	-4.837476	0.462835	1.346343
C	-5.952389	0.405807	0.598504
C	-6.079815	-0.328097	-0.715117
C	-7.213420	1.112212	1.040789
H	0.953261	0.595053	2.512117
H	-1.267771	0.483244	3.355691
H	-0.578080	-0.743948	4.430734
H	0.314644	-1.911003	2.238632
H	-2.433611	-3.875543	1.646227
H	-2.249890	-2.959952	0.155572
H	-0.806099	-3.540187	1.005427
H	-3.898243	-1.963434	2.239304
H	-3.129222	-0.678208	3.163364
H	-2.716000	0.599719	1.057574
H	-3.469611	-0.670731	0.112267
H	-4.890918	1.030650	2.277913
H	-6.385732	0.362740	-1.513170
H	-5.156251	-0.817266	-1.033118
H	-6.865427	-1.094239	-0.653820
H	-7.533421	1.853607	0.294938
H	-8.046210	0.402577	1.147490
H	-7.080291	1.627541	1.997431
H	4.269238	-0.892628	-1.524164
H	0.615311	5.412201	0.052103
H	-1.464659	4.695365	-1.535724
H	-3.070818	0.201963	-3.735738
H	-2.208588	-2.370802	-3.680681
H	1.541278	-4.983467	-1.096531
H	3.448329	-4.309456	0.713120
H	5.192010	0.219818	2.726422
H	4.509939	2.831326	2.456831

TS C1-HAT formation

(d, Cpd I + Nerol)

C	4.63920	-1.006724	0.206391
S	3.621168	0.222432	1.102265
H	4.242221	-2.019080	0.317897
H	5.642034	-0.974297	0.646257
N	2.137301	0.227568	-1.653405
N	1.376718	-1.816200	0.160825
N	1.471475	2.206187	0.305854
C	2.365933	2.658798	-1.935372
C	2.147913	-2.175679	-2.145216
C	0.588064	-2.277152	2.445718
C	0.608976	2.560672	2.576202
C	2.472516	1.346563	-2.377301
C	1.711788	-2.621373	-0.903288
C	0.452465	-0.964716	2.879443
C	1.082902	3.008094	1.350205
O	-0.244866	0.351269	-0.360738
C	2.929101	0.967021	-3.692718
C	1.558475	-4.008695	-0.542751
C	-0.016399	-0.582054	4.190495
C	1.262206	4.397774	1.001652
C	2.852871	-0.390887	-3.760489
C	1.134720	-4.035547	0.752468
C	-0.017546	0.778680	4.223796
C	1.765187	4.424749	-0.262739
C	2.356829	-0.843962	-2.483186
C	1.019596	-2.664887	1.183984
C	0.453450	1.227358	2.934414
C	1.891175	3.051591	-0.691100
N	0.735231	0.150296	2.133564
Fe	1.340025	0.188038	0.200047
H	2.666284	3.441989	-2.624176
H	2.369642	-2.926002	-2.897329
H	0.331472	-3.064451	3.147376
H	0.346639	3.311269	3.314892
C	-1.685819	-1.454244	-1.636839
O	-2.530084	-0.602552	-2.360174
C	-2.350487	-2.443420	-0.777317
C	-3.625151	-2.500961	-0.312152
C	-4.030698	-3.621033	0.611614
C	-4.705948	-1.487534	-0.615411
C	-4.598766	-0.227880	0.292702
C	-5.745868	0.726369	0.086263
C	-5.742470	1.884645	-0.594466
C	-4.538021	2.457985	-1.303687
C	-6.992138	2.728546	-0.693129
H	-1.969995	0.028779	-2.840299
H	-0.995056	-0.773917	-0.927461
H	-0.920783	-1.925259	-2.277400
H	-1.659042	-3.212318	-0.433754
H	-4.824363	-4.230577	0.157246
H	-3.193884	-4.278070	0.866822
H	-4.450400	-3.218363	1.544405
H	-4.651436	-1.161465	-1.655116
H	-5.688637	-1.951368	-0.460723
H	-4.587959	-0.552624	1.342790
H	-3.635236	0.253264	0.104226
H	-6.689061	0.412664	0.538306
H	-4.798983	2.728443	-2.336205
H	-4.202532	3.385657	-0.818010
H	-3.693001	1.767246	-1.344136
H	-6.821200	3.735251	-0.285552
H	-7.294300	2.867532	-1.740919
H	-7.832533	2.281577	-0.152041
H	2.032580	5.282508	-0.866431
H	1.030705	5.228585	1.655746
H	4.720090	-0.766053	-0.857016
H	-0.303906	1.435635	5.035024
H	-0.302337	-1.278700	4.968042
H	0.916623	-4.894410	1.374167

H	1.761157	-4.840596	-1.205009
H	3.108299	-1.042496	-4.586277
H	3.257753	1.664497	-4.452491

TS C1-HAT formation

(q, Cpd I + Nerol)

C	4.710051	-0.401406	0.183978
S	3.384139	-0.002890	1.373421
H	4.513213	-1.365532	-0.295681
H	5.646358	-0.479163	0.745296
N	2.191759	0.486081	-1.518108
N	1.436582	-1.813078	0.011077
N	1.184650	2.193283	0.529165
C	2.182079	2.939472	-1.587128
C	2.445288	-1.859675	-2.222118
C	0.493208	-2.541980	2.152744
C	0.227757	2.256994	2.788365
C	2.453422	1.685976	-2.127818
C	1.929687	-2.473083	-1.083983
C	0.280897	-1.289403	2.713532
C	0.699251	2.857876	1.629755
O	-0.284892	0.206599	-0.463894
C	3.023862	1.467862	-3.434621
C	1.838084	-3.901962	-0.887549
C	-0.291989	-1.074141	4.019869
C	0.779187	4.285980	1.425167
C	3.086425	0.118637	-3.615333
C	1.285975	-4.093047	0.343171
C	-0.362492	0.273447	4.204271
C	1.329168	4.477449	0.196070
C	2.558288	-0.487638	-2.417755
C	1.040946	-2.781464	0.897809
C	0.163426	0.885507	3.008657
C	1.587750	3.167193	-0.355135
N	0.561812	-0.079563	2.118581
Fe	1.278486	0.218666	0.261577
H	2.449531	3.807454	-2.181533
H	2.790833	-2.505182	-3.023674
H	0.216620	-3.405494	2.749558
H	-0.122217	2.907694	3.583415
C	-1.537773	-1.627280	-1.750174
O	-2.311151	-0.754542	-2.523367
C	-2.269810	-2.586807	-0.922056
C	-3.551911	-2.570841	-0.472860
C	-4.040826	-3.674714	0.430798
C	-4.559699	-1.482125	-0.771317
C	-4.378692	-0.247414	0.158151
C	-5.446872	0.793382	-0.054462
C	-5.344053	1.955018	-0.721476
C	-4.087053	2.442598	-1.403239
C	-6.524492	2.892072	-0.832467
H	-1.707206	-0.114457	-2.934061
H	-0.862330	-0.897243	-0.980086
H	-0.732152	-2.102552	-2.330398
H	-1.632559	-3.401324	-0.579440
H	-4.867493	-4.225964	-0.039289
H	-3.251302	-4.389070	0.683709
H	-4.442427	-3.260440	1.366617
H	-4.471547	-1.144263	-1.804936
H	-5.573720	-1.880630	-0.635841
H	-4.413148	-0.587109	1.203135
H	-3.378206	0.163459	-0.002948
H	-6.419186	0.547475	0.377662
H	-4.305276	2.730024	-2.441207
H	-3.698893	3.345032	-0.909224
H	-3.290886	1.695246	-1.424933
H	-6.286419	3.878387	-0.408881
H	-6.795374	3.065506	-1.883712
H	-7.406437	2.503616	-0.312661
H	1.553979	5.409315	-0.306833
H	0.457095	5.027310	2.145201
H	4.806429	0.367227	-0.586106
H	-0.738961	0.820215	5.059256
H	-0.597428	-1.864998	4.692941
H	1.064806	-5.025350	0.847029
H	2.162432	-4.644246	-1.605689
H	3.450784	-0.432173	-4.473086
H	3.324422	2.255748	-4.113316

TS C4-HAT formation

(d, Cpd I + Nerol)

C	2.190659	-2.952305	2.228085
S	1.157421	-3.096145	0.723869
H	3.102278	-2.379929	2.040536
H	2.468735	-3.971046	2.518749
N	2.113001	-0.115031	0.724500
N	1.189302	-1.283822	-1.718723
N	-0.394166	-0.655841	1.929924
C	1.468077	0.546246	2.997557
C	3.430687	-0.359050	-1.340314
C	-0.725356	-2.357155	-2.817886
C	-2.563749	-1.782284	1.626891
C	2.360543	0.481627	1.936992
C	2.459525	-0.961202	-2.126935
C	-1.583950	-2.380618	-1.726480
C	-1.622211	-1.092699	2.377600
O	-0.165636	0.577131	-0.583361
C	3.699776	1.015456	1.961532
C	2.643264	-1.335009	-3.509361
C	-2.910725	-2.948911	-1.747749
C	-1.808266	-0.706644	3.753019
C	4.256807	0.737393	0.749923
C	1.469298	-1.881252	-3.929168
C	-3.422466	-2.804403	-0.493599
C	-0.687169	-0.029265	4.131717
C	3.255425	0.042745	-0.022397
C	0.566427	-1.850488	-2.802565
C	-2.412831	-2.139309	0.293567
C	0.195804	-0.010588	2.993979
N	-1.302720	-1.894458	-0.474040
Fe	0.376013	-0.921518	0.107076
H	1.799336	1.037703	3.906488
H	4.395631	-0.164479	-1.797138
H	-1.086473	-2.783264	-3.748253
H	-3.494606	-2.049835	2.116217
C	-3.193624	1.423645	-1.921666
O	-3.820485	2.277038	-2.891853
C	-3.716716	1.685215	-0.537840
C	-3.017971	2.076436	0.550927
C	-3.712001	2.231653	1.887676
C	-1.558432	2.393093	0.556745
C	-1.106513	3.628788	-0.242976
C	0.310108	4.043862	0.047135
C	1.342552	4.156074	-0.804840
C	1.281112	3.868073	-2.285150
C	2.699768	4.601310	-0.311191
H	-3.649767	3.192330	-2.616587
H	-2.101625	1.497080	-1.966215
H	-3.459462	0.405468	-2.229411
H	-4.790683	1.531546	-0.428648
H	-3.261370	1.569205	2.637557
H	-3.604210	3.256936	2.266654
H	-4.779513	1.999869	1.820196
H	-1.165935	2.433046	1.579042
H	-0.925333	1.434283	0.074846
H	-1.277883	3.473276	-1.311745

H	-1.779889	4.451711	0.052657
H	0.497616	4.282039	1.096051
H	0.293887	3.549286	-2.626304
H	1.569741	4.756795	-2.863631
H	1.996926	3.077780	-2.549134
H	3.037963	5.501850	-0.843065
H	2.693520	4.820061	0.761428
H	3.456532	3.826482	-0.495576
H	1.636037	-2.493407	3.050510
H	5.249037	0.978005	0.390209
H	4.139236	1.530175	2.806348
H	3.559136	-1.188132	-4.067346
H	1.219171	-2.280348	-4.903804
H	-3.370356	-3.399395	-2.618179
H	-4.391634	-3.107744	-0.118847
H	-2.689840	-0.934245	4.338553
H	-0.457328	0.412791	5.092693

TS C4-HAT formation
(q, Cpd I + Nerol)

C	2.752034	-2.426204	2.257646
S	1.392710	-2.845238	1.115136
H	3.565714	-1.910558	1.742962
H	3.120828	-3.362648	2.687457
N	2.191226	0.152227	0.504046
N	1.218295	-1.332671	-1.715317
N	-0.177796	-0.474449	1.982423
C	1.642395	0.970693	2.760291
C	3.361341	-0.139775	-1.634251
C	-0.600072	-2.778446	-2.510590
C	-2.244695	-1.794613	1.942700
C	2.455665	0.887127	1.637891
C	2.409032	-0.923101	-2.268249
C	-1.379352	-2.763268	-1.358404
C	-1.335211	-0.941034	2.554792
O	-0.288534	0.522115	-0.548708
C	3.723269	1.561828	1.505376
C	2.540323	-1.441764	-3.610233
C	-2.635632	-3.456764	-1.212675
C	-1.473094	-0.414936	3.893149
C	4.216680	1.237399	0.277246
C	1.423025	-2.176201	-3.858882
C	-3.093461	-3.182134	0.040827
C	-0.384786	0.372599	4.120036
C	3.247344	0.368674	-0.344209
C	0.606471	-2.112112	-2.668356
C	-2.119358	-2.319408	0.662604
C	0.420644	0.322874	2.921785
N	-1.077071	-2.084958	-0.205236
Fe	0.516829	-0.882134	0.099199
H	1.994817	1.582983	3.584525
H	4.258490	0.108713	-2.192291
H	-0.961988	-3.363354	-3.350226
H	-3.125819	-2.075755	2.510870
C	-3.302113	1.034893	-2.113516
O	-3.860768	1.966258	-3.057933
C	-3.931938	1.168091	-0.756104
C	-3.346866	1.627823	0.375645
C	-4.124260	1.640159	1.676032
C	-1.960218	2.154833	0.461270
C	-1.605790	3.388107	-0.383500
C	-0.268567	3.988275	-0.041094
C	0.770724	4.239583	-0.853800
C	0.808746	3.937010	-2.332209
C	2.030622	4.877905	-0.315535
H	-3.768046	2.850385	-2.667591
H	-2.211984	1.125339	-2.060803
H	-3.525469	0.046440	-2.530556
H	-4.976926	0.862137	-0.703392
H	-3.595301	1.068128	2.448036
H	-4.227786	2.664638	2.058460
H	-5.125645	1.214484	1.557021
H	-1.639153	2.285809	1.499996
H	-1.113759	1.214322	0.040698
H	-1.683393	3.160913	-1.450363
H	-2.386339	4.143531	-0.181899
H	-0.159259	4.260106	1.010863
H	-0.094110	3.443496	-2.698490
H	0.951389	4.859411	-2.912703
H	1.662071	3.285961	-2.565713
H	2.262493	5.808906	-0.852062
H	1.947203	5.110851	0.751020
H	2.894958	4.213320	-0.451492
H	2.383870	-1.793024	3.071052
H	5.143969	1.552596	-0.183756
H	4.161773	2.198498	2.263113
H	3.388132	-1.258825	-4.257994
H	1.159625	-2.725154	-4.753912
H	-3.092923	-4.067152	-1.980839
H	-4.005941	-3.519557	0.515470
H	-2.297910	-0.635908	4.558630
H	-0.130068	0.933566	5.010157

TS C5-HAT formation
(d, Cpd I + Nerol)

C	1.033249	3.183216	2.703866
S	0.335356	3.228254	1.012243
H	0.407517	2.595163	3.380199
H	1.062515	4.217238	3.063983
N	1.941719	0.551609	0.981138
N	-0.743546	0.399594	1.882685
N	1.040717	1.671775	-1.493426
C	3.382600	1.259296	-0.882727
C	1.221592	-0.420886	3.114425
C	-3.075290	0.986436	1.353194
C	-0.940315	2.355537	-2.772007
C	3.164851	0.723279	0.380052
C	-0.139453	-0.182588	2.974370
C	-2.865553	1.484660	0.073998
C	0.425852	2.163867	-2.617776
O	0.055524	-0.547465	-0.612608
C	4.211234	0.221478	1.238151
C	-1.126073	-0.486023	3.979697
C	-3.910855	1.966313	-0.797006
C	1.414987	2.493886	-3.616653
C	3.605392	-0.271842	2.353364
C	-2.329058	-0.069325	3.492946
C	-3.310980	2.333591	-1.963381
C	2.631161	2.201429	-3.079412
C	2.187741	-0.063690	2.183850
C	-2.085172	0.479735	2.183524
C	-1.897970	2.084644	-1.802964
C	2.388872	1.685179	-1.752975
N	-1.650693	1.568709	-0.555640
Fe	0.137855	0.970197	0.183869
H	4.410449	1.330472	-1.223729
H	1.555876	-0.896190	4.030921
H	-4.093438	0.979637	1.728699
H	-1.285151	2.764782	-3.716137
C	2.141391	-2.829558	-2.072964
O	3.047299	-3.508440	-2.954845
C	2.107323	-3.585075	-0.778688
C	1.037443	-3.987947	-0.074045
C	1.219937	-4.726160	1.233669
C	-0.400946	-3.775458	-0.501723
C	-1.138648	-2.674383	0.294432

C	-2.605421	-2.564884	0.152478
C	-3.331387	-2.377504	-0.979394
C	-2.732633	-2.161760	-2.345121
C	-4.836512	-2.341455	-0.924245
H	3.183654	-2.931424	-3.722675
H	1.144470	-2.744291	-2.524804
H	2.494166	-1.803074	-1.884335
H	3.098846	-3.806832	-0.381414
H	2.278425	-4.860858	1.476790
H	0.749814	-4.193279	2.070874
H	0.748158	-5.717818	1.191457
H	-0.467921	-3.572254	-1.572704
H	-0.951896	-4.713670	-0.332501
H	-0.864478	-2.715723	1.352618
H	-0.637143	-1.620623	-0.072407
H	-3.165413	-2.602588	1.087284
H	-1.675662	-1.892889	-2.302280
H	-2.841396	-3.061245	-2.968002
H	-3.265883	-1.356083	-2.865349
H	-5.212912	-1.362957	-1.253947
H	-5.268356	-3.084889	-1.608759
H	-5.218899	-2.538080	0.081880
H	3.611993	2.316434	-3.522756
H	1.187223	2.902363	-4.592926
H	2.049898	2.782237	2.711525
H	-3.763883	2.742603	-2.857512
H	-4.959702	2.008436	-0.532542
H	-3.302680	-0.121752	3.963128
H	-0.906493	-0.948555	4.933443
H	4.058314	-0.735076	3.220563
H	5.265942	0.244983	0.995642

TS C5-HAT formation

(q, Cpd I + Nerol)

C	-1.003024	-3.078092	2.801350
S	0.384629	-2.918053	1.626165
H	-1.045862	-2.206314	3.461681
H	-0.816524	-3.966607	3.412580
N	-1.893530	-0.979292	0.594641
N	0.415202	0.043750	1.941278
N	-0.236479	-2.108654	-1.420575
C	-2.686116	-2.248423	-1.351931
C	-1.908951	0.415324	2.623447
C	2.867785	0.066577	1.926870
C	2.087805	-2.491037	-2.116056
C	-2.885435	-1.524840	-0.180247
C	-0.533940	0.568021	2.777558
C	3.066888	-0.689050	0.778384
C	0.715112	-2.609136	-2.277866
O	0.023263	0.447206	-0.702645
C	-4.180241	-1.208798	0.372170
C	0.098179	1.290756	3.857565
C	4.363191	-1.001971	0.228200
C	0.081356	-3.321605	-3.363423
C	-3.959514	-0.448186	1.480709
C	1.442676	1.187773	3.663033
C	4.146841	-1.718389	-0.910172
C	-1.259845	-3.260938	-3.144997
C	-2.530417	-0.303139	1.610550
C	1.631585	0.403754	2.464663
C	2.717192	-1.844266	-1.057336
C	-1.451211	-2.508170	-1.927009
N	2.078265	-1.223376	-0.015357
Fe	0.081350	-1.018244	0.222663
H	-3.566371	-2.626490	-1.862173
H	-2.549178	0.890668	3.360019
H	3.751771	0.423245	2.446250
H	2.721300	-2.943117	-2.872614

C	-2.744841	2.083916	-2.169729
O	-3.743968	2.574131	-3.077378
C	-2.809187	2.911421	-0.921298
C	-1.817582	3.578798	-0.308692
C	-2.093208	4.354883	0.960037
C	-0.390468	3.649550	-0.816548
C	0.618043	2.843258	0.024716
C	2.060350	3.049243	-0.166156
C	2.780088	3.093679	-1.319295
C	2.205845	2.858761	-2.694530
C	4.261008	3.370259	-1.280416
H	-3.802461	1.937287	-3.807067
H	-1.748924	2.110906	-2.629518
H	-2.951053	1.032032	-1.916492
H	-3.805218	2.950466	-0.477893
H	-3.141983	4.274685	1.262535
H	-1.475546	4.001670	1.796161
H	-1.855388	5.419336	0.824840
H	-0.339097	3.349378	-1.864476
H	-0.070750	4.703794	-0.786638
H	0.365193	2.878702	1.087141
H	0.368816	1.603121	-0.256538
H	2.633459	3.137736	0.757219
H	1.303651	2.243426	-2.667873
H	1.959856	3.807636	-3.192556
H	2.941568	2.352734	-3.331150
H	4.827302	2.520469	-1.687399
H	4.512727	4.236643	-1.908599
H	4.618846	3.568650	-0.265492
H	-2.061594	-3.684565	-3.736274
H	0.613806	-3.806455	-4.171693
H	-1.959391	-3.182125	2.284041
H	4.873815	-2.127225	-1.600256
H	5.305331	-0.699846	0.667432
H	2.245518	1.592353	4.266134
H	-0.433047	1.796257	4.653957
H	-4.684434	-0.014537	2.157704
H	-5.123654	-1.528024	-0.051902

TS C8-HAT formation

(d, Cpd I + Nerol)

C	4.900079	-1.146388	0.958429
S	3.762499	0.164819	1.537871
H	4.476611	-2.143838	1.100989
H	5.823409	-1.062787	1.541216
N	2.869843	0.659389	-1.333799
N	2.007173	-1.839461	-0.232759
N	1.564241	2.026997	0.798602
C	2.862385	3.088104	-0.997891
C	3.303422	-1.547987	-2.303874
C	0.785283	-2.909731	1.606804
C	0.117432	1.744283	2.758413
C	3.213153	1.939452	-1.694387
C	2.663571	-2.320976	-1.340338
C	0.357139	-1.752449	2.245302
C	0.835827	2.516880	1.852141
O	0.351915	0.265753	-0.828875
C	3.988129	1.923015	-2.914205
C	2.619664	-3.762388	-1.357519
C	-0.448427	-1.730579	3.442554
C	0.917911	3.957351	1.889936
C	4.103325	0.619956	-3.288638
C	1.930914	-4.149466	-0.247302
C	-0.652807	-0.420821	3.752821
C	1.699458	4.332766	0.839574
C	3.402036	-0.161514	-2.294788
C	1.545212	-2.943655	0.442825
C	0.035646	0.357103	2.750014

C	2.091706	3.122428	0.158488
N	0.631017	-0.471794	1.829106
Fe	1.703493	0.090952	0.227847
H	3.204275	4.038144	-1.396612
H	3.777970	-2.075321	-3.125714
H	0.494436	-3.860204	2.042940
H	-0.408169	2.268000	3.550887
C	-6.464476	-1.054345	0.823012
O	-7.574226	-1.680474	1.481195
C	-6.772203	0.366773	0.443067
C	-6.653878	0.953211	-0.761286
C	-7.030697	2.406098	-0.944117
C	-6.151046	0.256696	-2.010329
C	-4.683314	0.608324	-2.399481
C	-3.676650	0.157269	-1.387361
C	-2.658304	-0.735579	-1.542031
C	-2.317761	-1.434903	-2.837743
C	-1.789458	-1.027045	-0.409507
H	-8.330361	-1.618077	0.875356
H	-6.134165	-1.649493	-0.038837
H	-5.652467	-1.081811	1.560071
H	-7.151679	0.965500	1.272570
H	-6.184521	2.999309	-1.316110
H	-7.832436	2.509481	-1.688408
H	-7.371340	2.858444	-0.007723
H	-6.230503	-0.830735	-1.917814
H	-6.789690	0.550161	-2.854665
H	-4.480311	0.181815	-3.385855
H	-4.605173	1.700300	-2.510888
H	-3.798209	0.594272	-0.396206
H	-2.339736	-2.523744	-2.703038
H	-2.993087	-1.181220	-3.657084
H	-1.297670	-1.174108	-3.146842
H	-1.359160	-2.030765	-0.371741
H	-0.668240	-0.348512	-0.584529
H	-2.130907	-0.670637	0.563186
H	5.148470	-1.012179	-0.098755
H	0.433610	4.581711	2.629746
H	1.989474	5.330413	0.535685
H	4.382014	2.804375	-3.403953
H	4.613394	0.205844	-4.149000
H	3.066585	-4.381086	-2.125137
H	1.691725	-5.151627	0.084676
H	-1.203138	-0.001043	4.585125
H	-0.798884	-2.611876	3.964396

TS C8-HAT formation

(q, Cpd I + Nerol)

C	4.715341	-1.320242	1.186898
S	3.531887	-0.050071	1.750379
H	4.224042	-2.296049	1.120974
H	5.514315	-1.383332	1.931894
N	2.847066	-0.609949	-1.292353
N	1.269916	-1.749041	0.807566
N	2.254028	1.999593	-0.346844
C	3.680688	1.480509	-2.273929
C	2.364557	-3.005525	-0.989227
C	-0.008826	-1.223449	2.834881
C	1.099627	3.274547	1.407546
C	3.566071	0.095677	-2.225369
C	1.593911	-2.915156	0.166982
C	0.107521	0.160409	2.787973
C	1.868587	3.173415	0.257442
O	0.315573	0.223164	-0.785131
C	4.154216	-0.805367	-3.185851
C	1.042194	-4.045033	0.879062
C	-0.497004	1.059522	3.740378
C	2.424347	4.302931	-0.451899
C	3.764467	-2.062922	-2.836141
C	0.386892	-3.542826	1.962411
C	-0.193147	2.324840	3.336850
C	3.162565	3.800033	-1.477772
C	2.945676	-1.934762	-1.655604
C	0.533394	-2.107130	1.910058
C	0.599696	2.200289	2.137901
C	3.055033	2.361375	-1.403515
N	0.779840	0.878321	1.825549
Fe	1.743313	0.147497	0.198867
H	4.287059	1.904813	-3.067816
H	2.538999	-3.996888	-1.395651
H	-0.575887	-1.647336	3.657709
H	0.879530	4.272050	1.774471
C	-6.521187	-1.127523	0.745540
O	-7.680067	-1.727568	1.341455
C	-6.730413	0.335265	0.472422
C	-6.569055	0.999692	-0.685595
C	-6.848359	2.483990	-0.758787
C	-6.109639	0.367095	-1.984428
C	-4.625950	0.660643	-2.355244
C	-3.641288	0.072196	-1.389750
C	-2.644951	-0.826612	-1.633240
C	-2.320024	-1.392755	-2.996647
C	-1.772741	-1.254326	-0.541885
H	-8.420944	-1.566596	0.734885
H	-6.216250	-1.675952	-0.155631
H	-5.724801	-1.264262	1.487392
H	-7.072778	0.895385	1.344031
H	-5.962465	3.045964	-1.083603
H	-7.636550	2.695876	-1.494585
H	-7.164650	2.886266	0.208522
H	-6.252695	-0.717783	-1.969401
H	-6.733945	0.757050	-2.800257
H	-4.452308	0.300852	-3.373566
H	-4.483757	1.751544	-2.386150
H	-3.755646	0.408806	-0.359044
H	-2.358867	-2.489415	-2.975381
H	-2.992474	-1.045097	-3.783443
H	-1.296477	-1.118682	-3.283101
H	-1.368251	-2.265990	-0.629190
H	-0.683660	-0.544062	-0.618020
H	-2.130086	-1.030725	0.465606
H	5.139183	-1.068299	0.212075
H	2.265576	5.337858	-0.176864
H	3.736024	4.334481	-2.224303
H	4.772899	-0.496442	-4.018631
H	4.000122	-3.002063	-3.320259
H	1.157675	-5.078489	0.578015
H	-0.150549	-4.077900	2.734906
H	-0.472696	3.265463	3.793906
H	-1.079212	0.745130	4.597126

Table S11 The number of models generated using Chai-1 for each design/substrate pair and the ligand SMILES notation used for modeling.

Model	Number of models generated	Substrate SMILES notation
Design 18/α-damascone (5)	50	C/C=C/C(=O)C1C(=CCCC1(C)C)C
Design 18/β-damascone (6)	10	O=C(/C1=C(/CCCC1(C)C)C)/C=C/C
Design 18/α-ionone (8)	10	CC1=CCCC(C1/C=C/C(=O)C)(C)C
Design 18/δ-damascone (7)	10	C/C=C/C(=O)C1C(C=CCC1(C)C)C
Design 11/δ-damascone (7)	10	C/C=C/C(=O)C1C(C=CCC1(C)C)C

II Supplementary Figs

Fig. S1 Calibration curves for determining TON using GC-MS for **A)** citral A, **B)** citral B, **C)** 2,3-epoxy nerol), **D)** carveol.

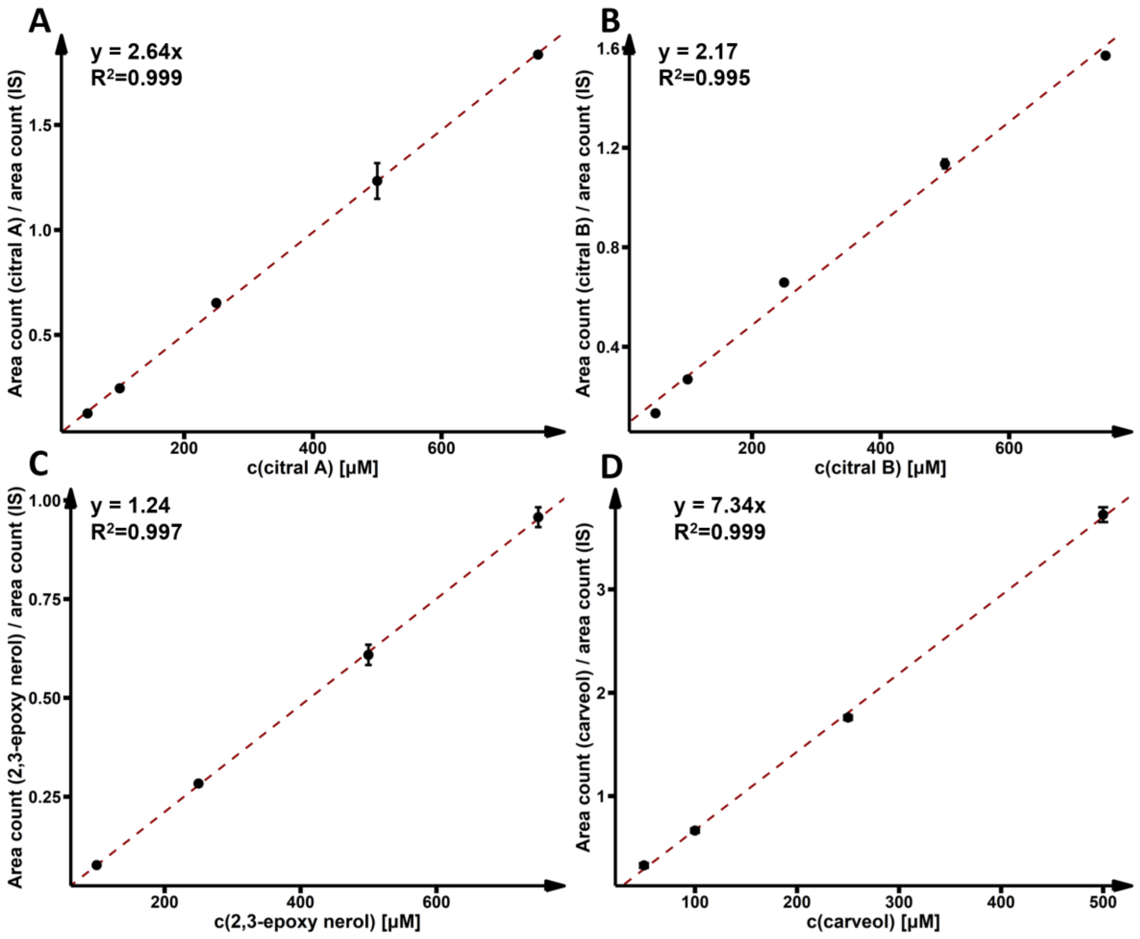

Fig. S2 Fragmentation of 2-hydroxy-δ-damascone.

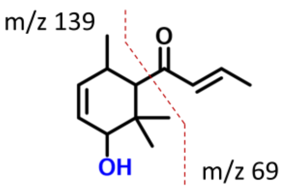

Fig. S3 GC-MS chromatogram after the reaction of (*S*)-(-)-limonene with wt *Mth*UPO and extraction with EtOAc (IS: 4-octanone).

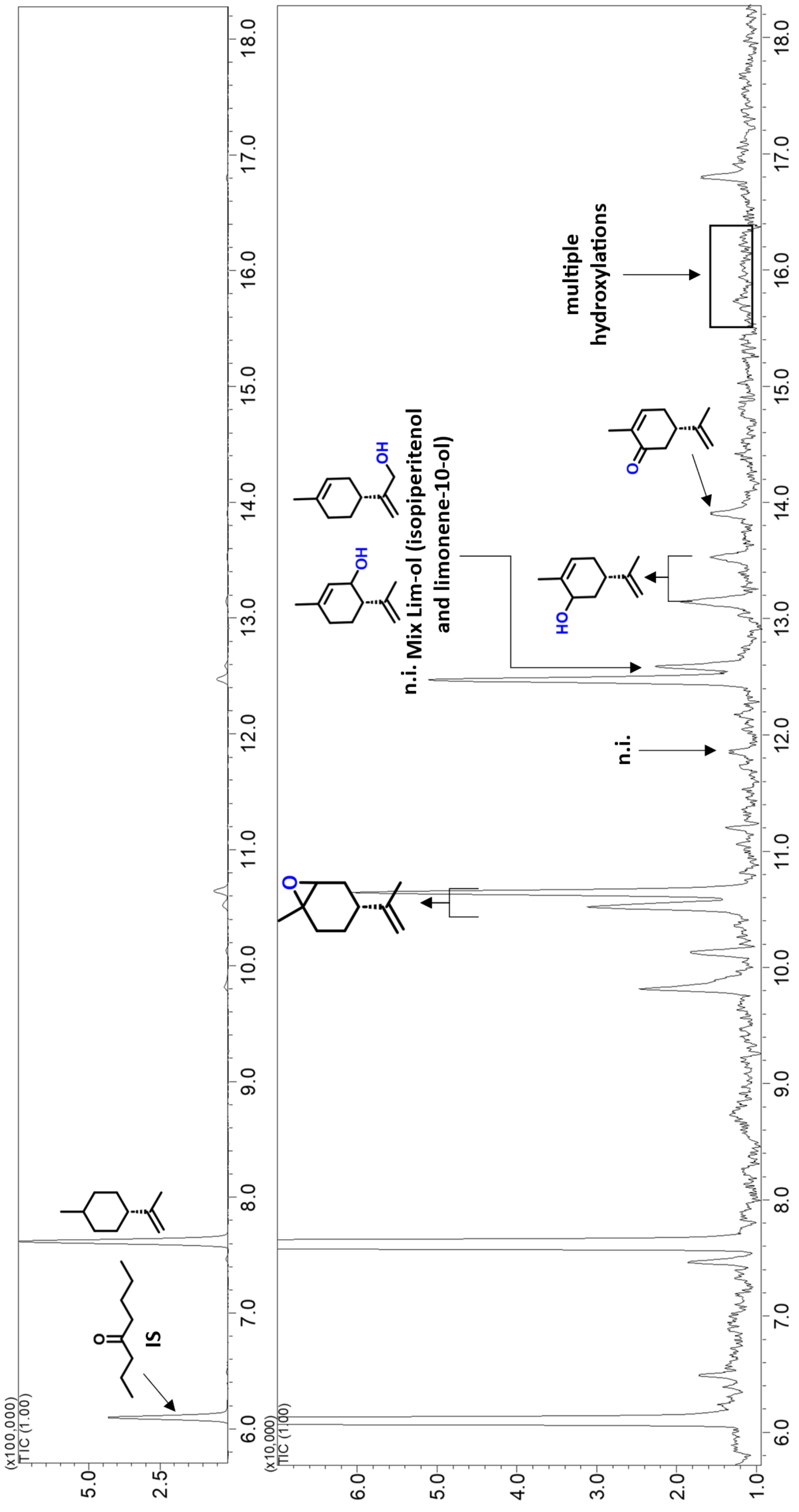

Fig. S4 Chiral GC-MS chromatograms after enzymatic conversion of β-ionone catalyzed by **A)** wt *Mth*UPO, **B)** FuncLib Var 11, **C)** FuncLib Var 34.

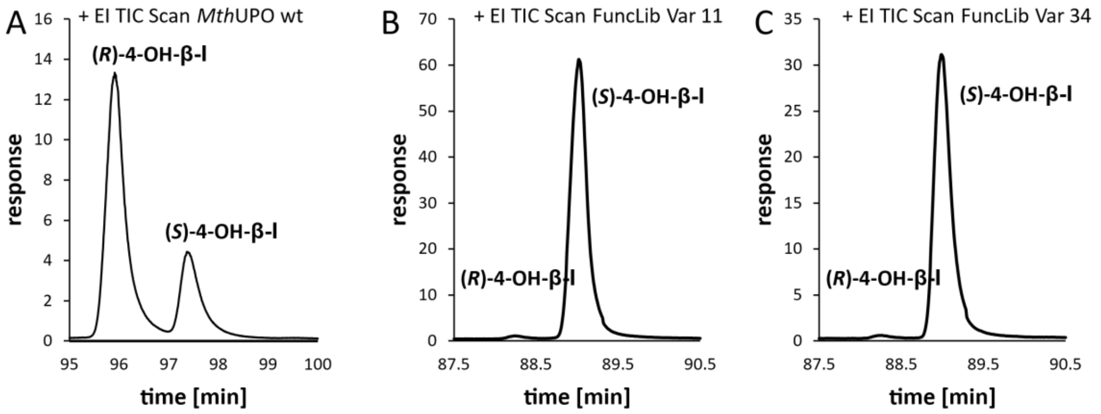

Fig. S5 GC-MS chromatogram after the reaction of **A** δ-damascone and **B** α-damascone with wt *Mth*UPO (grey) and different FuncLib variants after extraction with EtOAc (β-ionone, elution time 12.25 min).

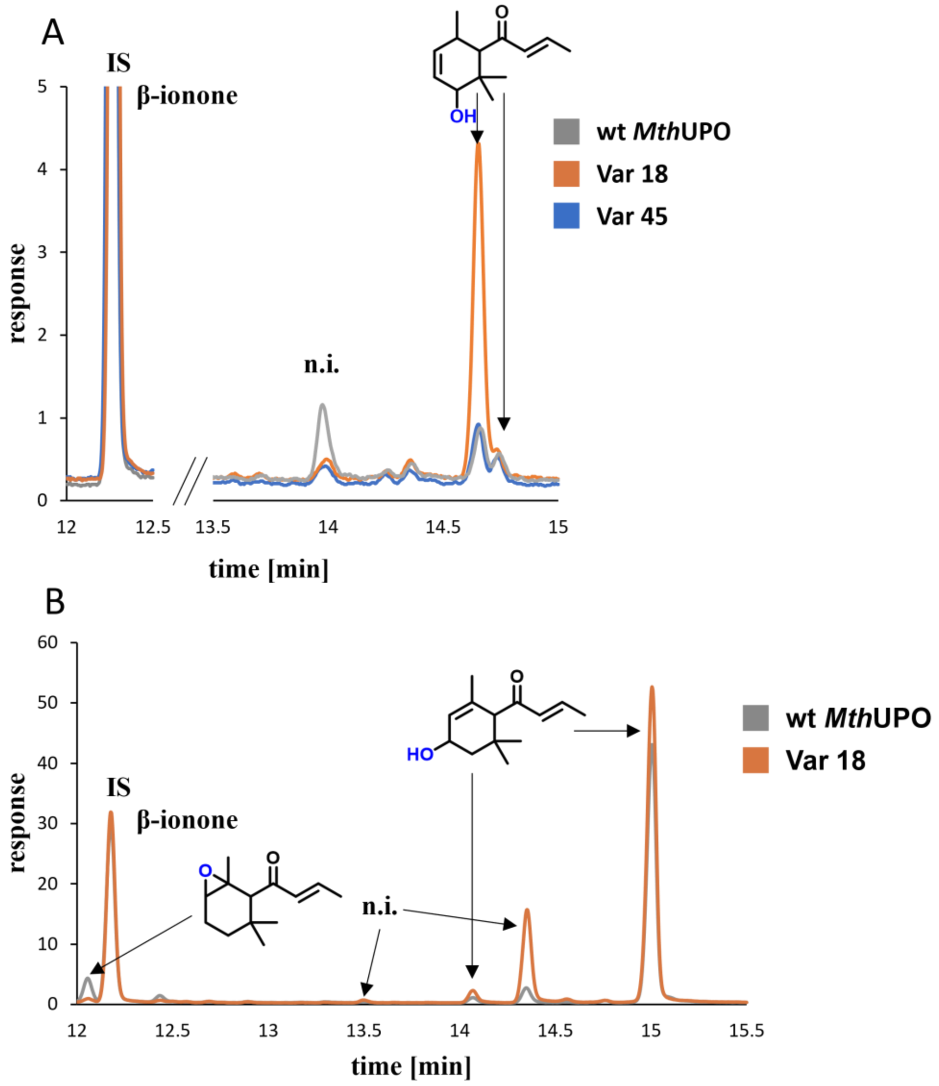

Fig. S6 DFT model calculations exploring the intrinsic oxidation pattern of geraniol.

A) Schematic structure and atom numbering of geraniol (**1**).

B) DFT computed relative energies using a computational truncated model based on *Mth*UPO active site (iron-oxo species coordinated to the porphyrin pyrrole core and methane thiolate as the axial ligand) for all rate-limiting transition states (TS) leading to different oxidation products of **1**, including TS C2-O formation involved in geraniol epoxidation and Hydrogen Atom Transfers (TS HAT) involved in geraniol hydroxylation. Energy values were obtained at the (U)B3LYP/Def2TZVP/PCM(dichloromethane)// (U)B3LYP/6-31G(d)+SDD(Fe)/PCM(dichloromethane) level (see details on the Computational Methods section). The reported relative energies were computed in doublet (d) and quartet (q) electronic states in terms of electronic energy (ΔE), enthalpy (ΔH), and quasi-harmonic corrected Gibbs energy (ΔG). All energies are referred considering the quartet separated reactants **[Cpd I + geraniol (q)]** as zero.

C) Optimized geometries for the stationary points reported in B). Energies are referred considering as zero the lowest in energy TS **[TS C1-HAT (q)]**.

All energies are given in kcal·mol^{-1}. Distances, and angles are given in Angstrom (Å) or degrees (º), respectively.

A)

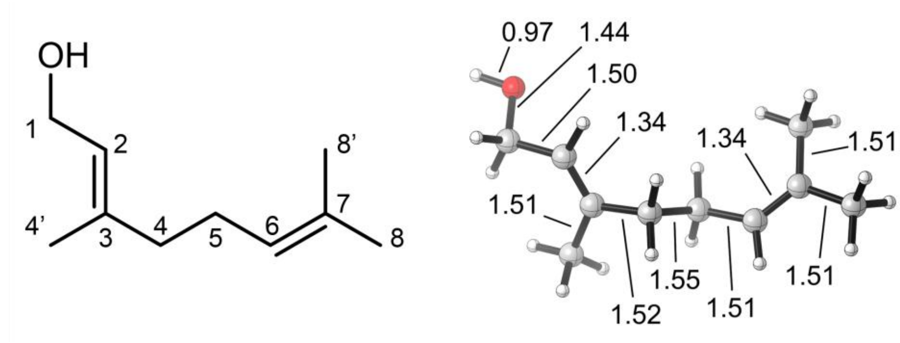

B)

Cpd I + Geraniol	Electronic state	ΔE	ΔH	ΔG
**TS C2-O formation**	doublet (d)	3.1	3.3	20.5
	quartet (q)	3.2	3.5	21.2
**TS C1-HAT**	doublet (d)	4.8	2.1	19.6
	quartet (q)	2.9	-0.1	18.3
**TS C4-HAT**	doublet (d)	7.1	3.5	21.1
	quartet (q)	6.9	3.4	21.1
**TS C5-HAT**	doublet (d)	5.8	2.9	20.9
	quartet (q)	7.5	4.4	21.9
**TS C8-HAT**	doublet (d)	9.3	6.6	23.5
	quartet (q)	9.2	6.0	23.2

C)

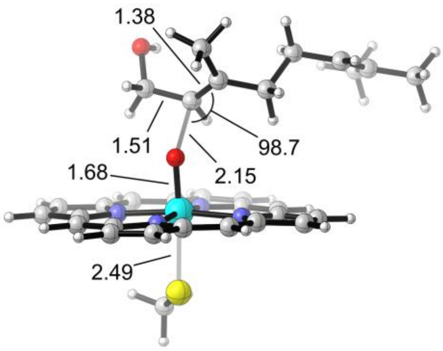

Cpd I + Geraniol
TS C2-O formation (d)
ΔΔG‡ = 2.2 (ΔΔE‡ = 0.3)

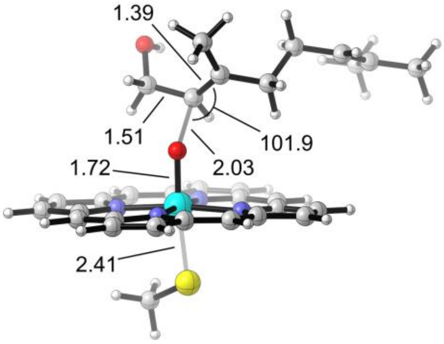

Cpd I + Geraniol
TS C2-O formation (q)
ΔΔG‡ = 2.9 (ΔΔE‡ = 0.3)

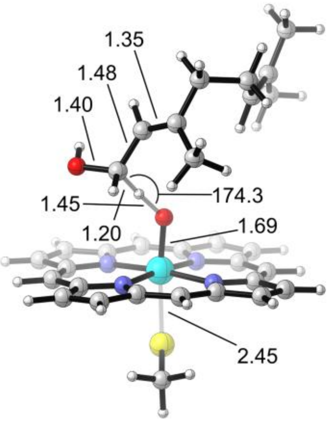

Cpd I + Geraniol
TS C1-HAT (d)
ΔΔG‡ = 1.3 (ΔΔE‡ = 2.0)

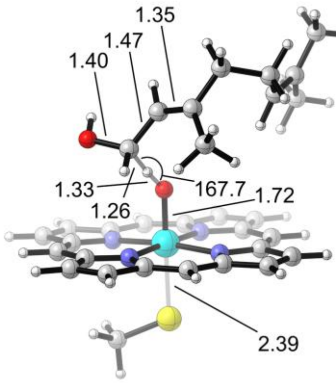

Cpd I + Geraniol
TS C1-HAT (q)
ΔΔG‡ = 0.0 (ΔΔE‡ = 0.0)

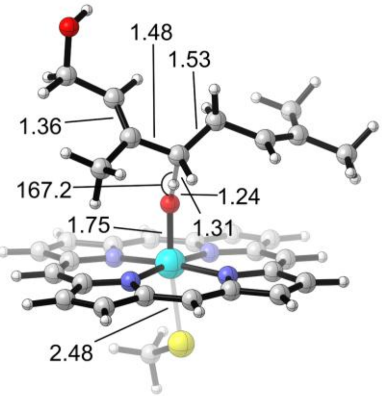

Cpd I + Geraniol
TS C4-HAT (d)
ΔΔG‡ = 2.8 (ΔΔE‡ = 4.2)

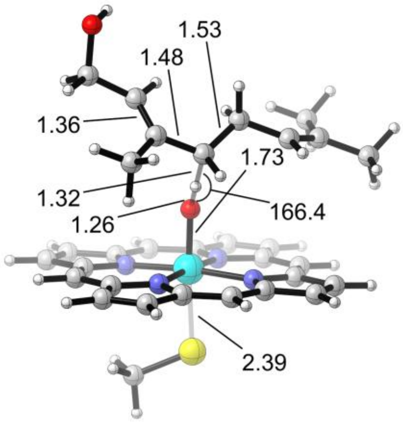

Cpd I + Geraniol
TS C4-HAT (q)
ΔΔG‡ = 2.7 (ΔΔE‡ = 4.0)

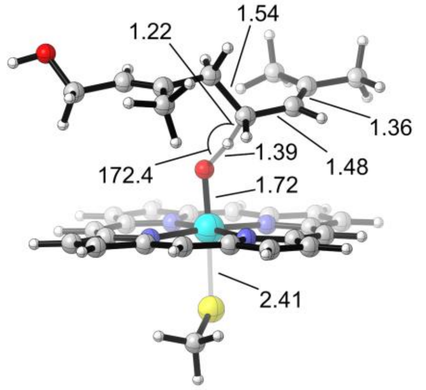

Cpd I + Geraniol
TS C5-HAT (d)
ΔΔG‡ = 2.6 (ΔΔE‡ = 3.0)

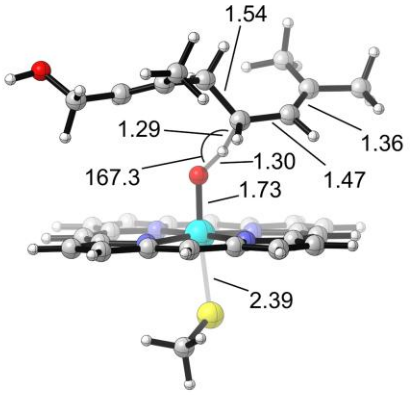

Cpd I + Geraniol
TS C5-HAT (q)
ΔΔG‡ = 3.6 (ΔΔE‡ = 4.6)

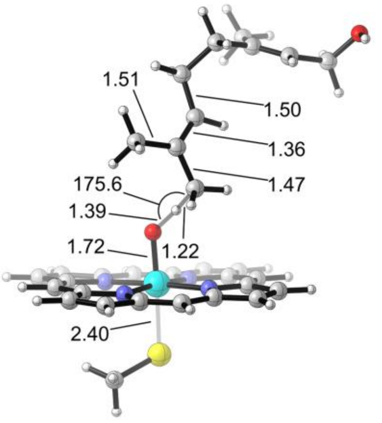

Cpd I + Geraniol
TS C8-HAT (d)
ΔΔG‡ = 5.2 (ΔΔE‡ = 6.4)

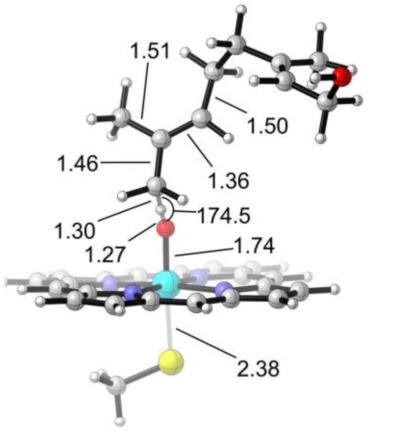

Cpd I + Geraniol
TS C8-HAT (q)
ΔΔG‡ = 4.9 (ΔΔE‡ = 6.3)

Truncated DFT models for geraniol oxidation by Cpd I indicates that allylic hydroxylation at C1 is the most energy accessible oxidation (**TS C1-HAT**), followed by epoxidation at the double C2-C3 bond (**TS C2-O formation**). The hydroxylations at positions 4, 5, and 8 are more energetically demanding and are not expected to occur in *Mth*UPO active site.

Fig. S7 DFT model calculations exploring the intrinsic oxidation pattern of nerol.

A) Schematic structure and atom numbering of nerol (**2**).

B) DFT computed relative energies using a computational truncated model based on *Mth*UPO active site (iron-oxo species coordinated to the porphyrin pyrrole core and methane thiolate as the axial ligand) for all rate-limiting transition states (TS) leading to different oxidation products of **2**, including TS C2-O formation involved in geraniol epoxidation and Hydrogen Atom Transfers (TS HAT) involved in geraniol hydroxylation. Energy values were obtained at the (U)B3LYP/Def2TZVP/PCM(dichloromethane)// (U)B3LYP/6-31G(d)+SDD(Fe)/PCM(dichloromethane) level (see details on the Computational Methods section). The reported relative energies were computed in doublet (d) and quartet (q) electronic states in terms of electronic energy (ΔE), enthalpy (ΔH), and quasi-harmonic corrected Gibbs energy (ΔG). All energies are referred considering the quartet separated reactants **[Cpd I + nerol (q)]** as zero.

C) Optimized geometries for the stationary points reported in B). Energies are referred considering as zero the lowest in energy TS **[TS C1-HAT (q)]**.

All energy is given in kcal·mol^{-1}. Distances, and angles are given in Angstrom (Å) or degrees (º), respectively.

A)

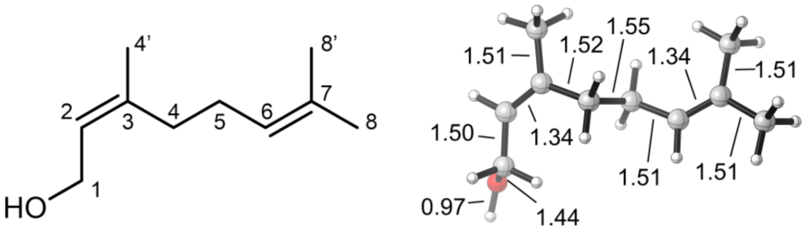

B)

Cpd I + Nerol	Electronic state	ΔE	ΔH	ΔG
**TS C2-O formation**	doublet (d)	1.5	1.8	19.5
(conformer 1)	quartet (q)	2.6	2.9	21.3
**TS C2-O formation**	doublet (d)	-2.6	-2.2	17.0
(conformer 2)	quartet (q)	-2.3	-2.0	17.4
**TS C1-HAT**	doublet (d)	0.3	-2.5	16.4
	quartet (q)	-0.1	-3.1	16.0
**TS C4-HAT**	doublet (d)	4.8	1.6	20.1
	quartet (q)	5.6	2.3	21.1
**TS C5-HAT**	doublet (d)	4.6	1.7	19.9
	quartet (q)	4.7	1.5	19.8
**TS C8-HAT**	doublet (d)	11.5	8.6	26.2
	quartet (q)	8.0	4.8	22.4

C)

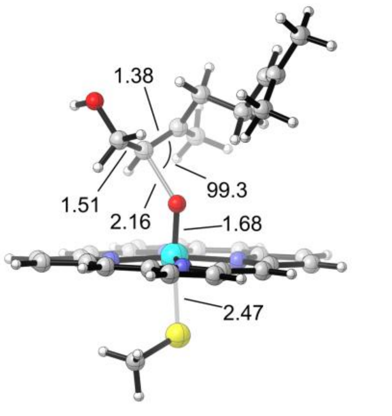

Cpd I + Nerol
TS C2-O formation
(conformer 1, d)
ΔΔG‡ = 3.5 (ΔΔE‡ = 1.6)

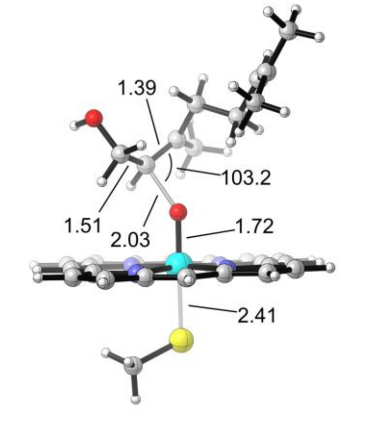

Cpd I + Nerol
TS C2-O formation
(conformer 1, q)
ΔΔG‡ = 5.3 (ΔΔE‡ = 2.7)

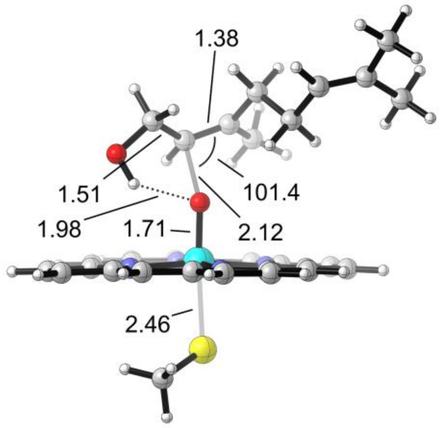

Cpd I + Nerol
TS C2-O formation
(conformer 2, d)
ΔΔG‡ = 1.0 (ΔΔE‡ = -2.5)

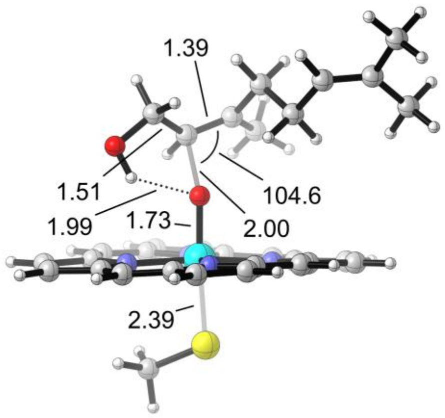

Cpd I + Nerol
TS C2-O formation
(conformer 2, q)
ΔΔG‡ = 1.4 (ΔΔE‡ = -2.2)

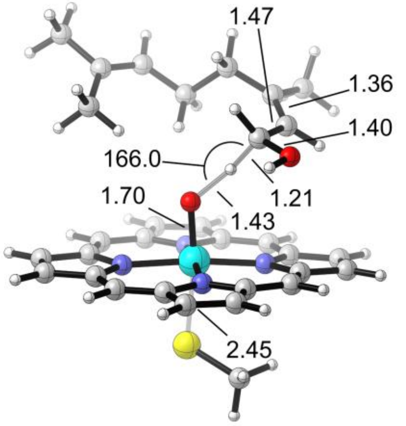

Cpd I + Nerol
TS C1-HAT (d)
ΔΔG‡ = 0.4 (ΔΔE‡ = 0.4)

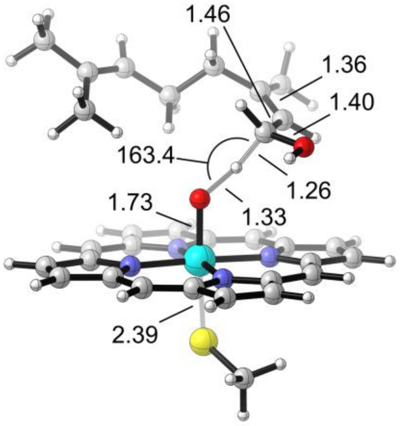

Cpd I + Nerol
TS C1-HAT (q)
ΔΔG‡ = 0.0 (ΔΔE‡ = 0.0)

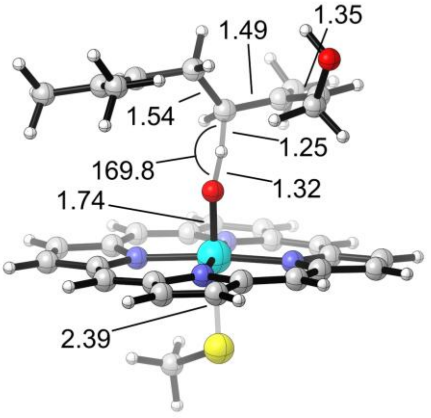

Cpd I + Nerol
TS C4-HAT (d)
ΔΔG‡ = 4.1 (ΔΔE‡ = 4.9)

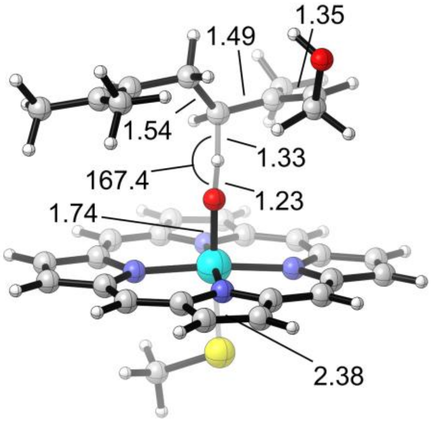

Cpd I + Nerol
TS C4-HAT (q)
ΔΔG‡ = 5.1 (ΔΔE‡ = 5.7)

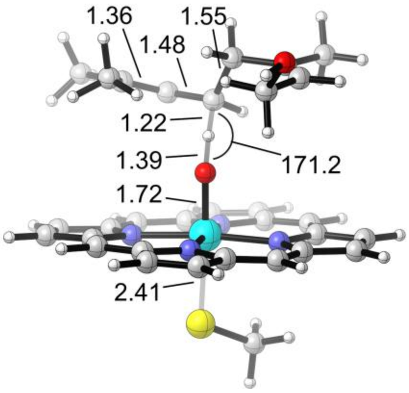

Cpd I + Nerol
TS C5-HAT (d)
ΔΔG‡ = 3.9 (ΔΔE‡ = 4.7)

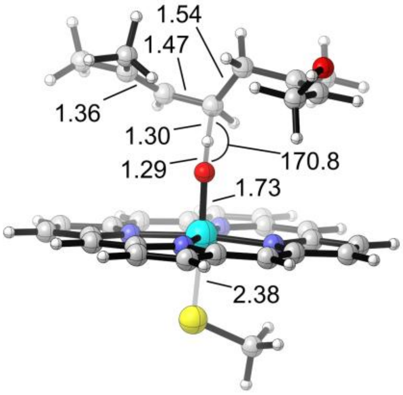

Cpd I + Nerol
TS C5-HAT (q)
ΔΔG‡ = 3.9 (ΔΔE‡ = 4.8)

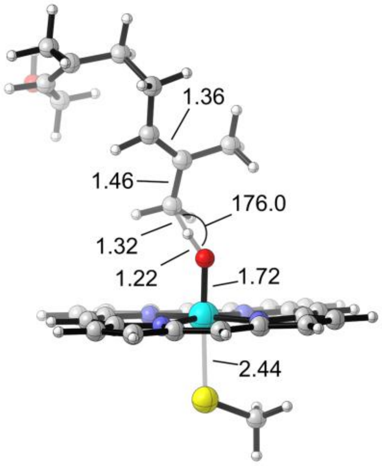

Cpd I + Nerol
TS C8-HAT (d)
ΔΔG‡ = 10.2 (ΔΔE‡ = 11.6)

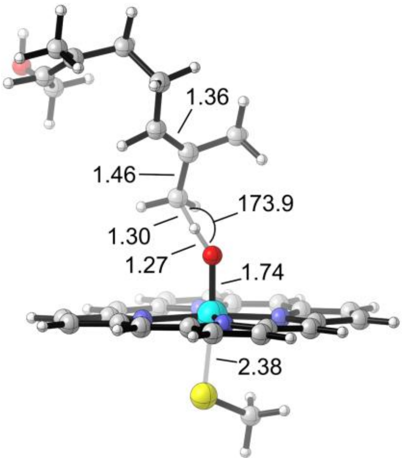

Cpd I + Nerol
TS C8-HAT (q)
ΔΔG‡ = 6.4 (ΔΔE‡ = 8.1)

Truncated DFT models for nerol oxidation by Cpd I indicates that allylic hydroxylation at C1 is the most energy accessible oxidation (**TS C1-HAT**), followed by epoxidation at the double C2-C3 bond (**TS C2-O formation**).

MD simulations on Design 28 with nerol bound in the active site (**Fig. S11**) indicate that the substrate can perform a hydrogen bond with the iron-oxo group of the Cpd I species. This alternative conformation is also tested using truncated DFT models (**TS C2-O formation**, *conformer 2*), but even if lower in energy than *conformer 1*, it is found to be slightly higher in energy than HAT at C1.

The hydroxylations at positions 4, 5, and 8 are more energetically demanding and are not predicted to occur in *Mth*UPO active site.

Fig. S8 Analysis of geraniol (**1**) binding mode in Design 4 (F63I/F154L/G157A/ A161F) through substrate-bound MD simulations.

The following analysis allows to characterize which is the preferred binding pose of the substrate in the active site and to elucidate which position of the substrate is more accessible for effective near-attack conformations (NACs) that resemble the geometric requirements of the DFT optimized TS during the MD simulations. Only substrate positions that exhibit a low energetic cost for oxidation as determined by truncated DFT calculations in **Fig. S6** are analyzed in the MD simulations (C2-C3 epoxidation or C1 hydroxylation).

The following geometric parameters are used to characterize the near-attack conformations in the heat map plots: the O–C2 distance and O–C2=C3 angle are used to characterize NACs for effective C2-C3 epoxidation while O–H1 distance and O–H1–C1 angle are used to characterize NACs for effective C1 hydroxylation.

The resulting heat maps were obtained from 3 independent MD simulations of 500 ns each (a total of 1500 ns) without any restraint on the substrate. Ideal geometric parameters obtained from DFT Transition State (TS) optimizations (**Fig. S6**) are shown as black dots. A schematic representation of the different geometric parameters used for the heat maps are also provided in each subfigure.

A) NACs for C1-HAT leading to geraniol hydroxylation.

B) NACs for C2-O formation leading to geraniol epoxidation.

C) NACs for pro-*R* and pro-*S* C1-HAT leading to enantioselective geraniol hydroxylation.

D) Polar interaction between the terminal alcohol group of geraniol and the sidechain of E158.

E) Most representative structure of the binding mode of geraniol in Design 4 active site as determined by clustering analysis.

Distances and angles are given in angstroms (Å) and degrees (º), respectively.

A)

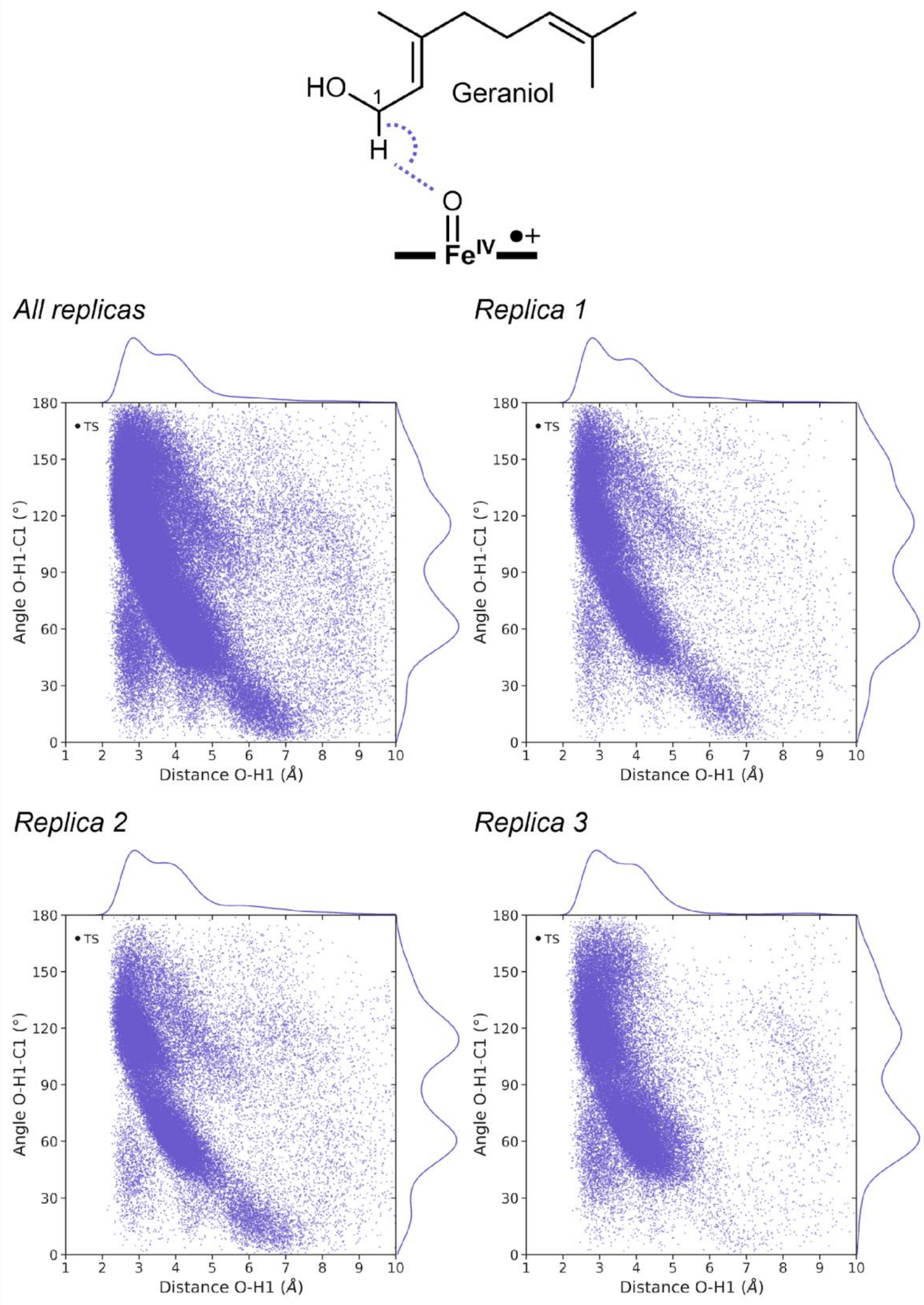

HO
1
Geraniol
H
O
Fe^{IV} •+
All replicas
Replica 1
Replica 2
Replica 3
• TS
Angle O-H1-C1 (°)
Distance O-H1 (Å)

B)

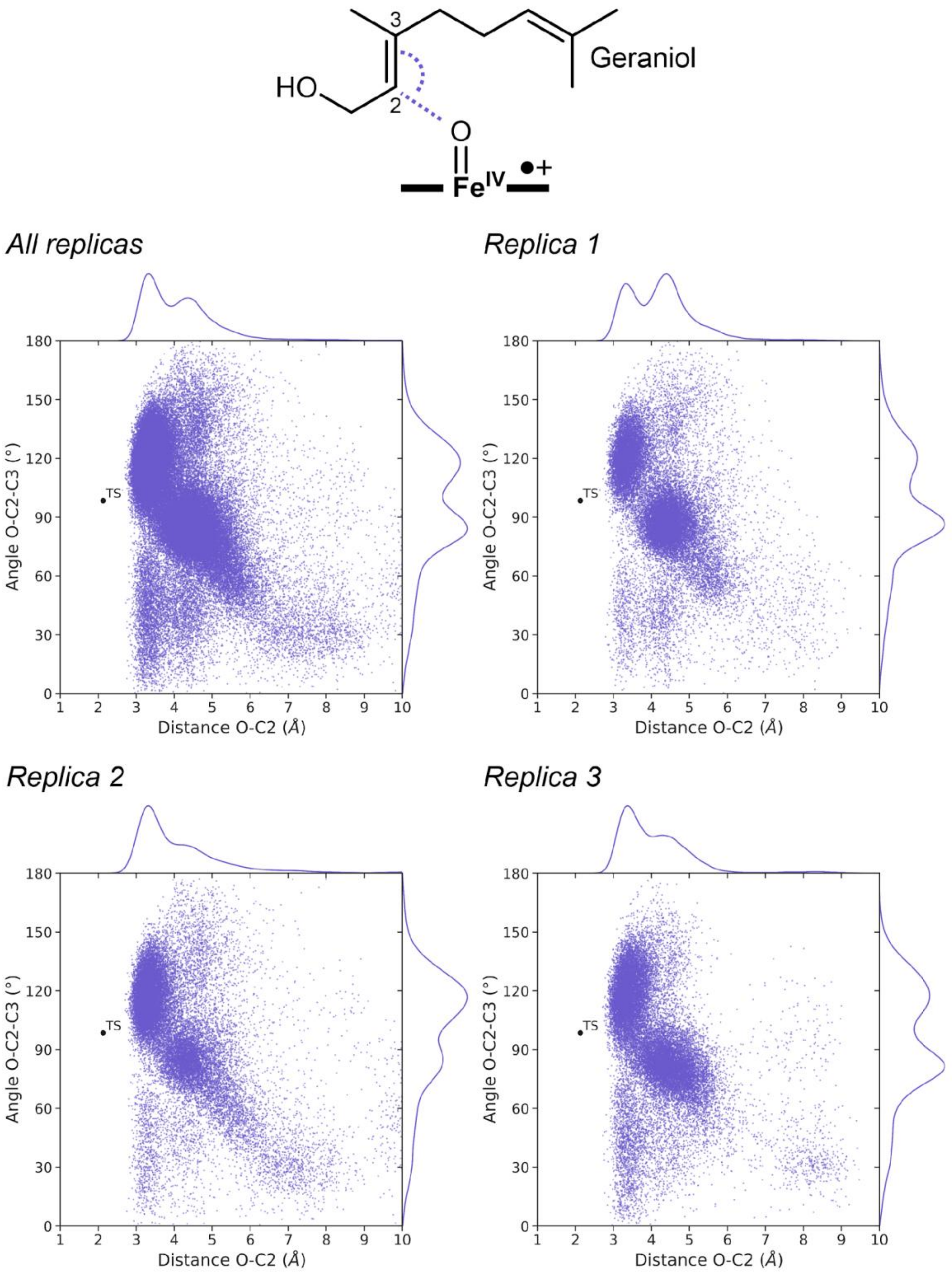

3
2
HO
Geraniol
O
Fe^{IV} •+
All replicas
Replica 1
Replica 2
Replica 3
Angle O-C2-C3 (°)
Distance O-C2 (Å)
TS

C)

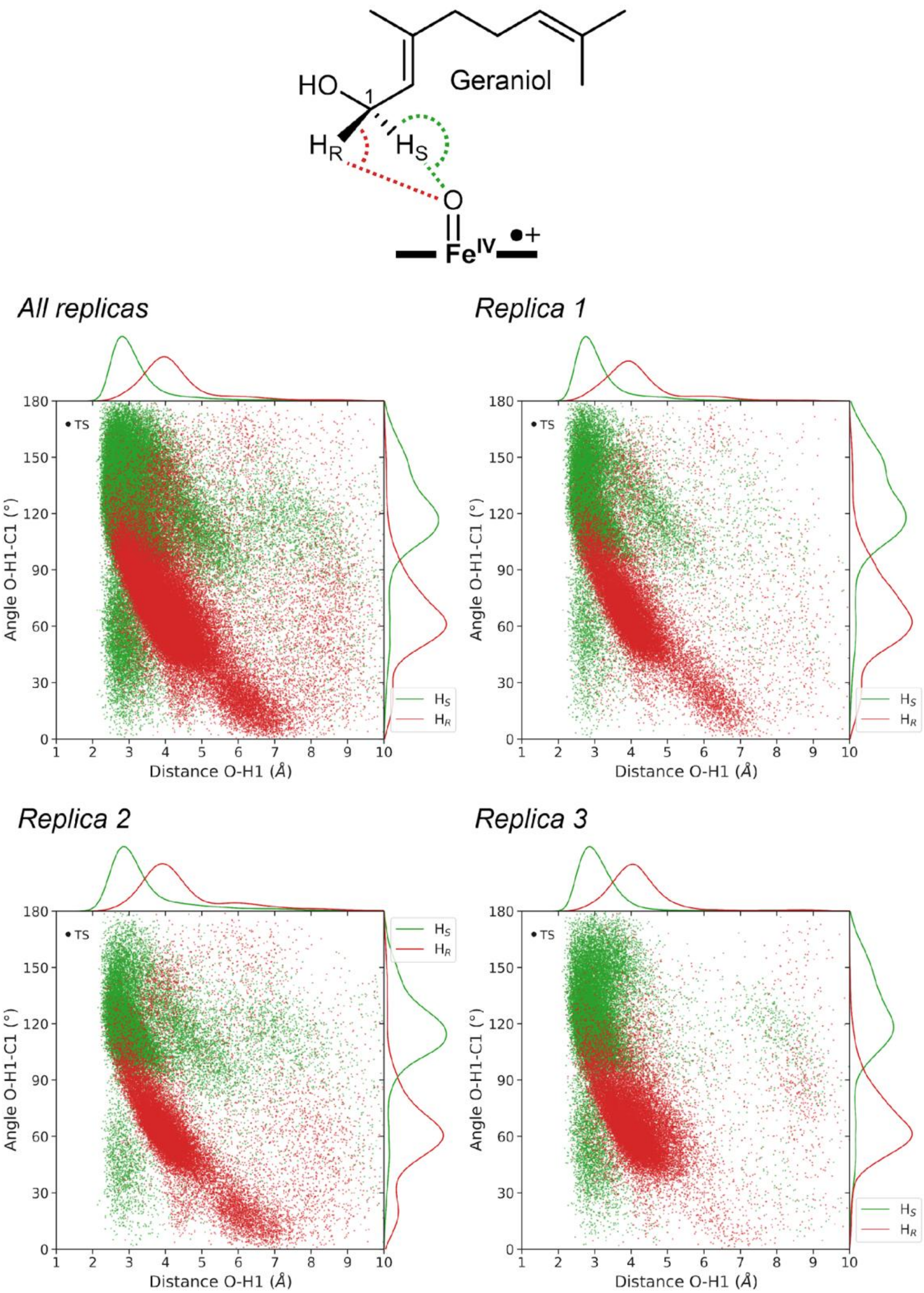

Geraniol
HO
1
HR
HS
O
FeIV •+
All replicas
Replica 1
Replica 2
Replica 3
• TS
HS
HR
Angle O-H1-C1 (°)
Distance O-H1 (Å)

D)

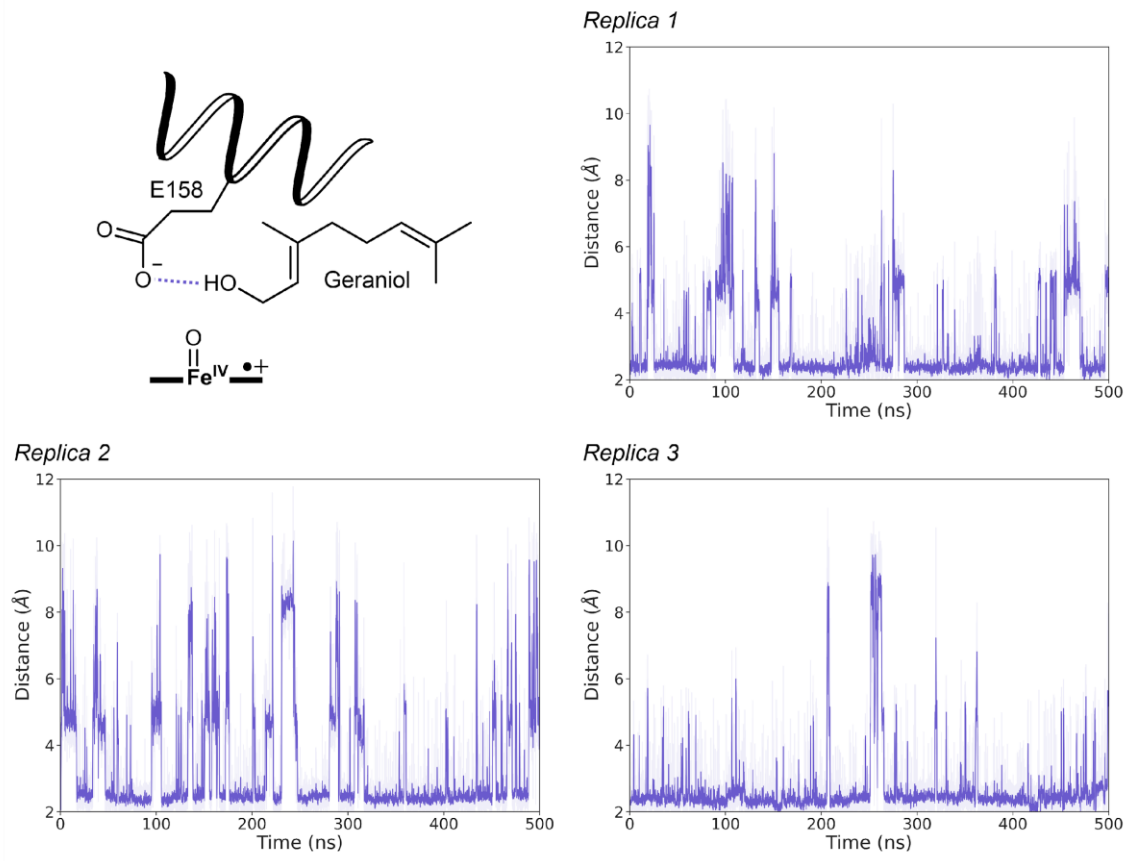

E158
O
O
HO
Geraniol
O
Fe^IV •+
Replica 1
Replica 2
Replica 3
Distance (Å)
Time (ns)

E)

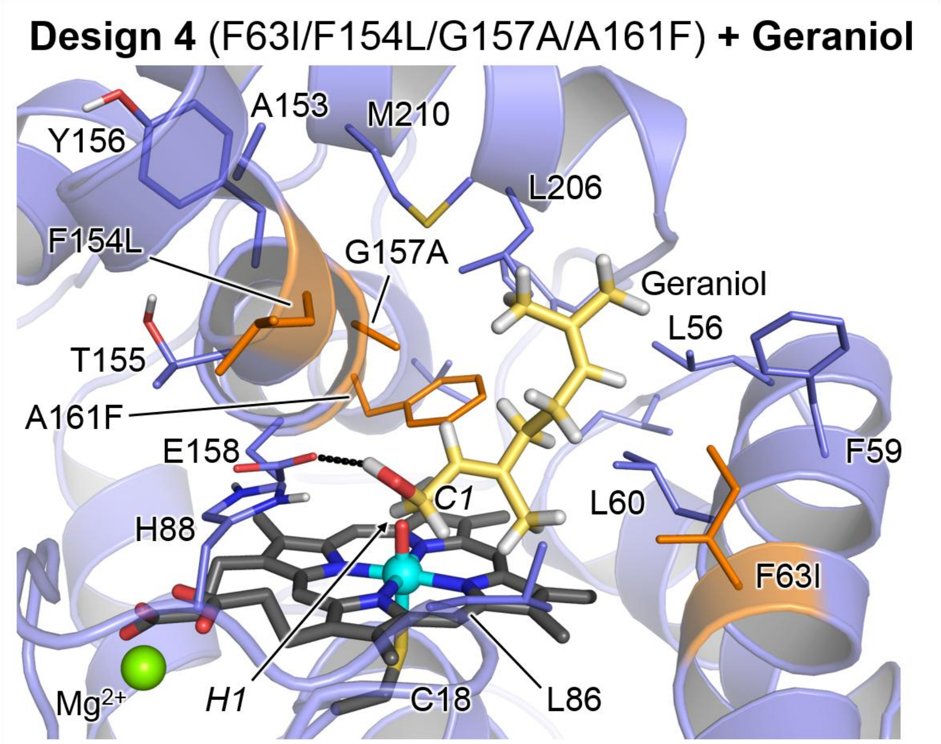

Design 4 (F63I/F154L/G157A/A161F) + Geraniol
Y156
A153
M210
L206
F154L
G157A
Geraniol
L56
T155
A161F
F59
E158
C1
L60
H88
F63I
Mg2+
H1
C18
L86

MD simulations with geraniol bound on Design 4 active site indicate that due to active site reshaping induced by the sidechain substitutions (specially by A161A, see **Fig. S8 E**), the C2-C3 double bond of geraniol is located slightly far from the catalytic iron-oxo species compared to C1, where HAT is more geometrically accessible (**Fig. S8-A** and **B**). This is further supported by truncated DFT models with geraniol (**Fig. S6**) where allylic hydroxylation at C1 is intrinsically less energetically demanding than C2-C3 epoxidation.

These simulations also suggest a preference for enantioselective pro-*S* HAT at C1 (**Fig. S8-C**).

Geraniol binds to the active site of Design 4 by anchoring its terminal alcohol group with the catalytic E158 residue (**Figs S8-D** and **E**).

Fig. S9 Analysis of geraniol (**1**) binding mode in Design 2 (F63L/A153I/F154I/G157A) through substrate-bound MD simulations.

The following analysis allows to characterize which is the preferred binding pose of the substrate in the active site and to elucidate which position of the substrate is more accessible for effective near-attack conformations (NACs) that resemble the geometric requirements of the DFT optimized TS during the MD simulations. Only substrate positions that exhibit a low energetic cost for oxidation as determined by truncated DFT calculations in **Fig. S6** are analyzed in the MD simulations (C2-C3 epoxidation or C1 hydroxylation).

The following geometric parameters are used to characterize the near-attack conformations in the heat map plots: the O–C2 distance and O–C2=C3 angle are used to characterize NACs for effective C2-C3 epoxidation while O–H1 distance and O–H1–C1 angle are used to characterize NACs for effective C1 hydroxylation. The O-C2=C3-C4 dihedral angle is used to determine which enantioface of the C2-C3 bond of the substrate is exposed to the catalytic iron-oxo moiety of Cpd I. Positive dihedral values indicate that the pro-*S* face is exposed while negative values indicate that the pro-*R* face is exposed instead.

The resulting heat maps were obtained from 3 independent MD simulations of 500 ns each (a total of 1500 ns) without any restrain on the substrate. Ideal geometric parameters obtained from DFT Transition State (TS) optimizations (**Fig. S6**) are shown as black dots. A schematic representation of the different geometric parameters used for the heat maps are also provided in each subfigure.

A) NACs for C1-HAT leading to geraniol hydroxylation.

B) NACs for C2-O formation leading to geraniol epoxidation.

C) NACs for pro-*R* and pro-*S* C2-O formation leading to enantioselective geraniol epoxidation.

D) Polar interaction between the terminal alcohol group of geraniol and the sidechain of E158.

E) Most representative structure of the binding mode of geraniol in Design 2 active site as determined by clustering analysis.

Distances and angles are given in angstroms (Å) and degrees (º), respectively.

A)

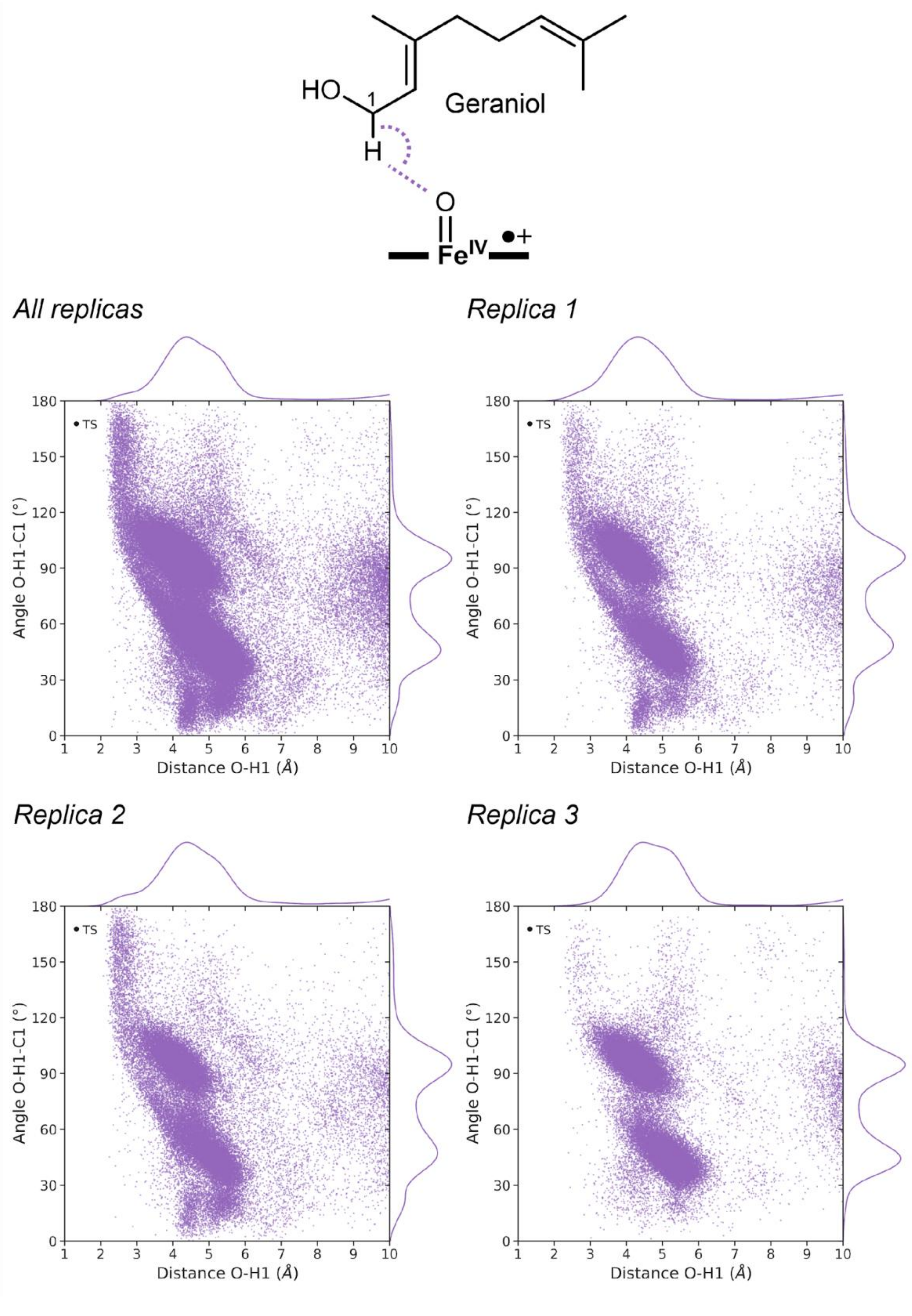

HO
1
H
Geraniol
O
Fe^IV
•+
All replicas
Replica 1
Replica 2
Replica 3
• TS
Angle O-H1-C1 (°)
Distance O-H1 (Å)
0
30
60
90
120
150
180
1
2
3
4
5
6
7
8
9
10

B)

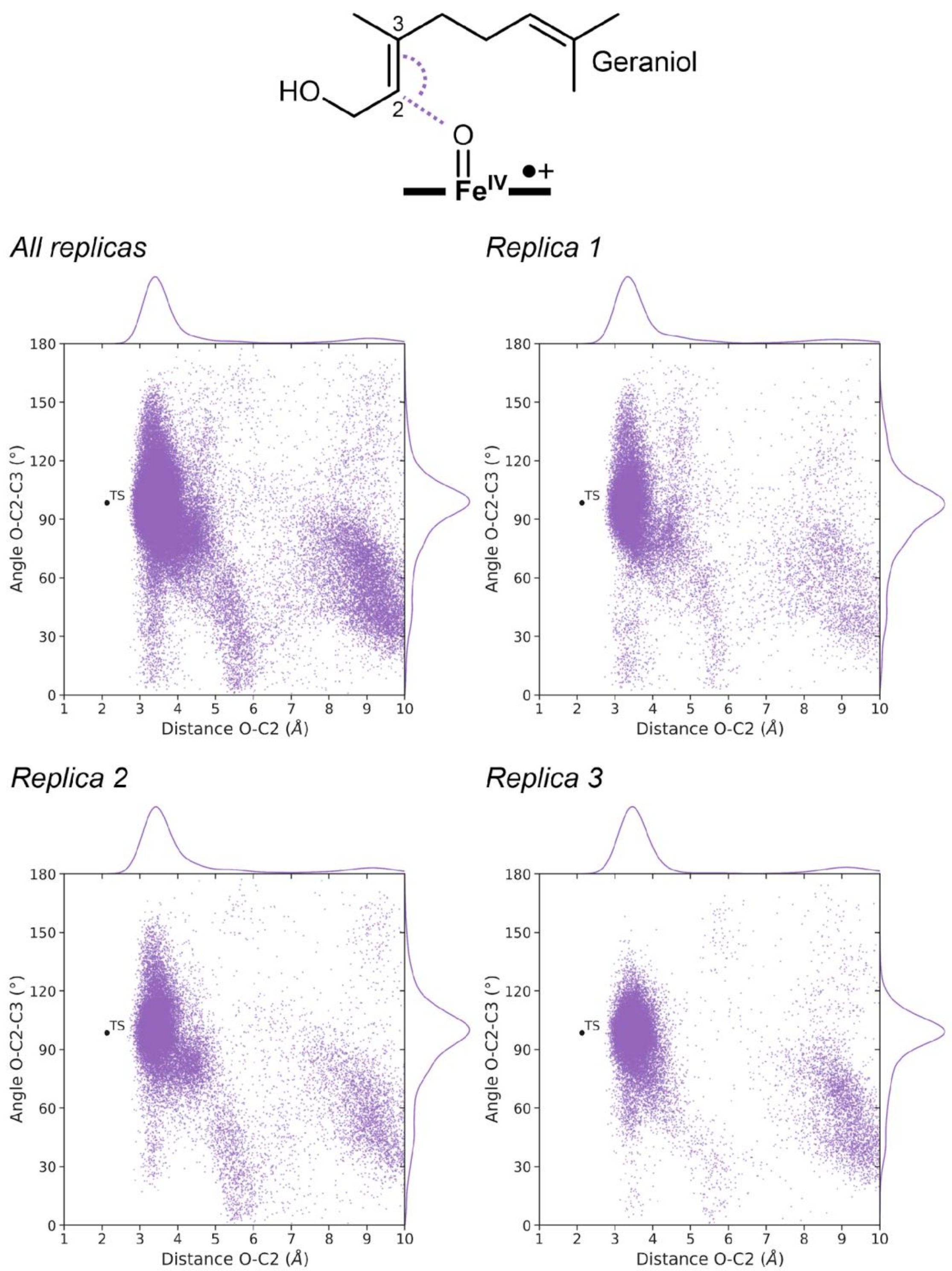

3
Geraniol
HO
2
O
Fe^{IV} •+
All replicas
Replica 1
Replica 2
Replica 3
Angle O-C2-C3 (°)
Distance O-C2 (Å)
TS

C)

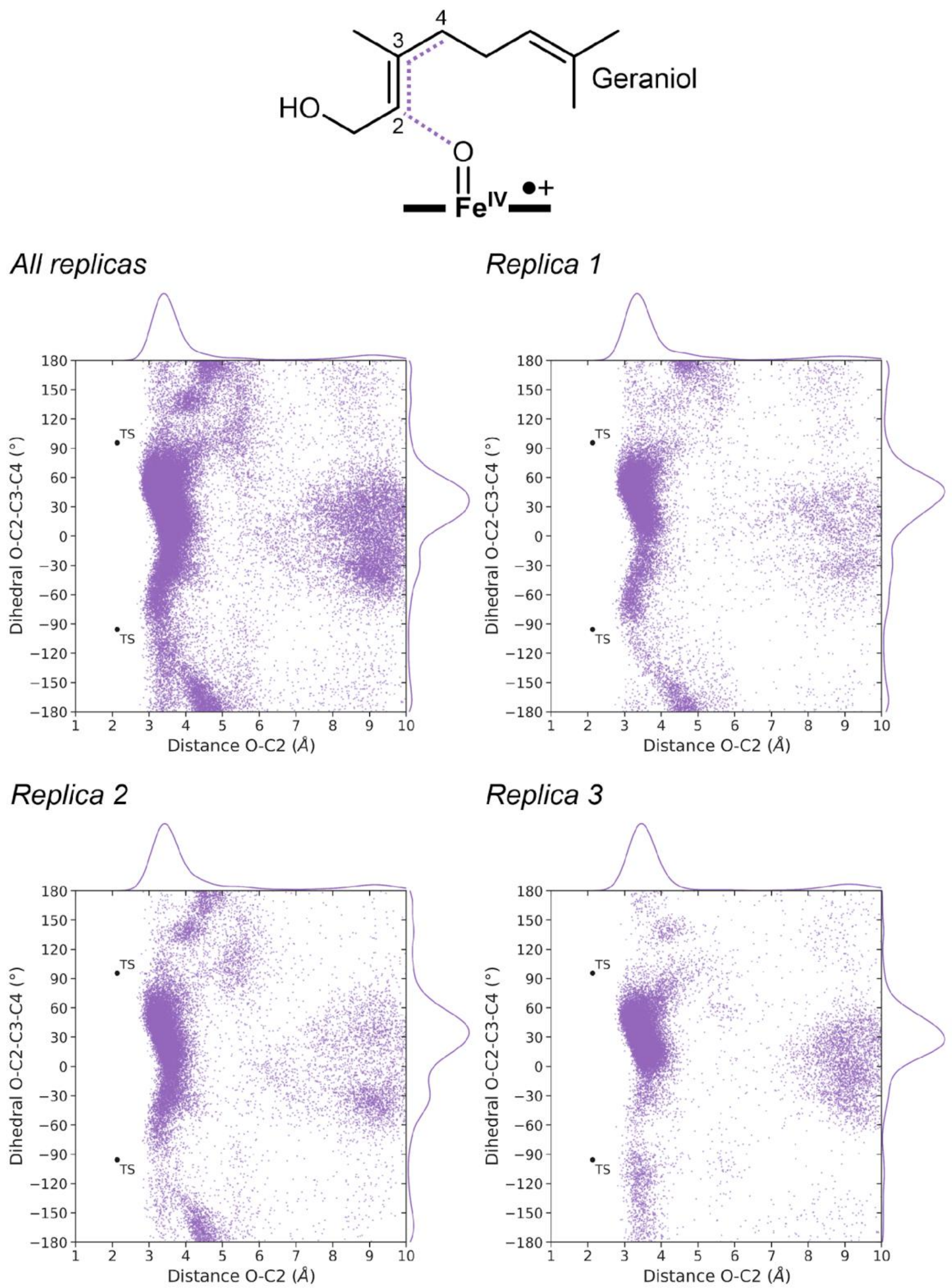

Geraniol
HO
2
3
4
O
Fe^IV
•+
All replicas
Replica 1
Replica 2
Replica 3
TS
Dihedral O-C2-C3-C4 (°)
Distance O-C2 (Å)

D)

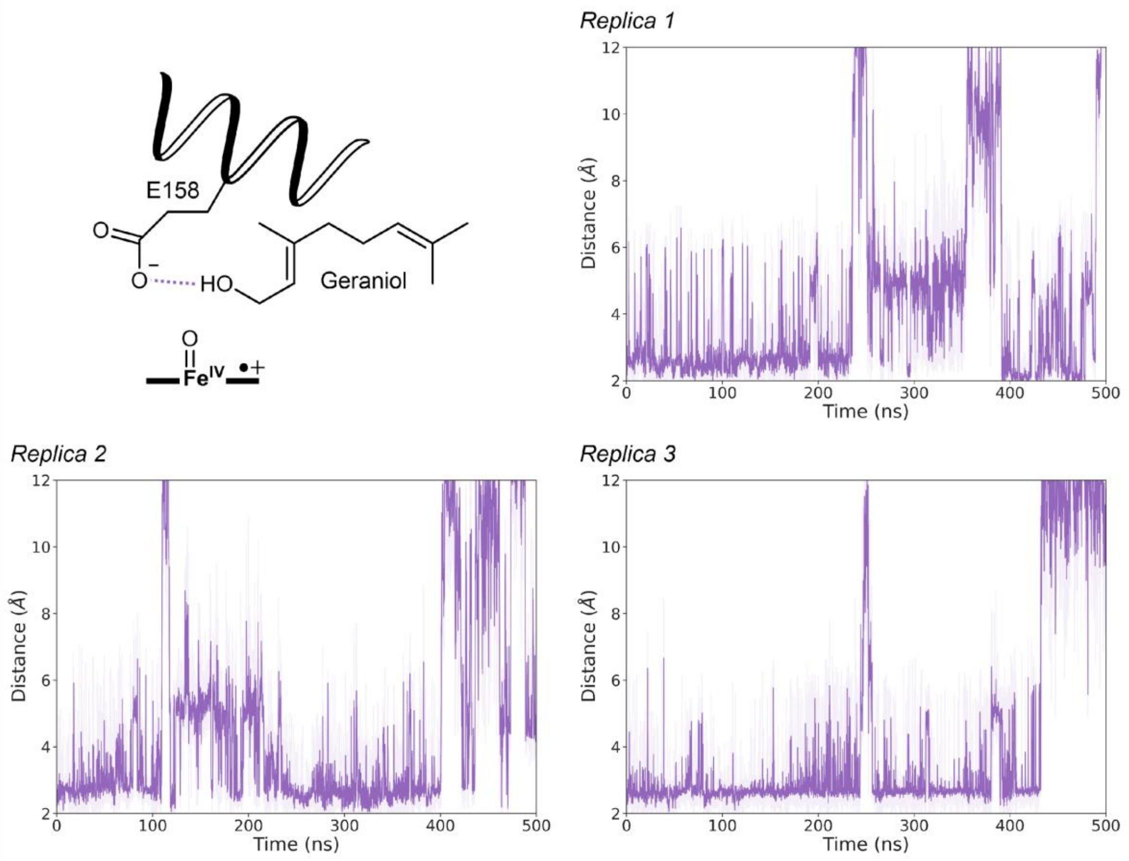
E158
Geraniol
HO
Fe^IV
Replica 1
Replica 2
Replica 3
Distance (Å)
Time (ns)
0
100
200
300
400
500
2
4
6
8
10
12

E)

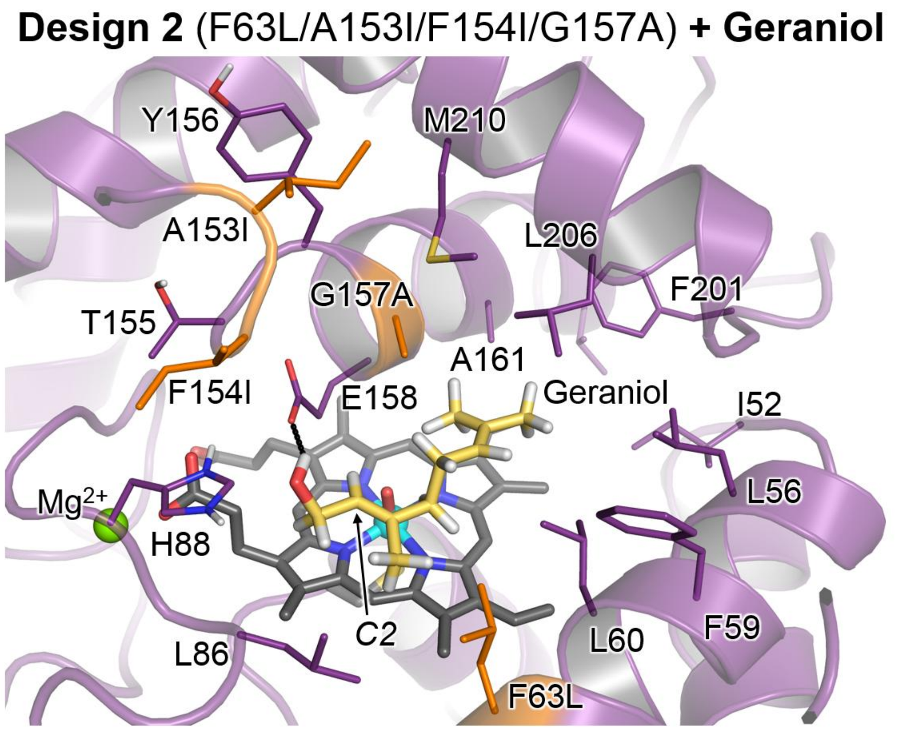
Design 2 (F63L/A153I/F154I/G157A) + Geraniol
Y156
M210
A153I
L206
T155
G157A
F201
A161
F154I
E158
Geraniol
I52
L56
Mg2+
H88
C2
L86
L60
F59
F63L

As in Design 4 (see **Fig. S8**), geraniol in Design 2 anchors its hydroxyl group on the catalytic E158 residue (**Figs S9 D** and **E**). However, Design 2 has a less sterically congested active site than Design 4 and geraniol can lay closer to the active Cpd I species, positioning the double C2-C3 bond next to the catalytic iron-oxo group (**Fig. S9 B** and **E**). As a consequence, the C-H bonds at C1 are not geometrically preorganized for effective HAT since they are not pointing to the catalyst (**Fig. S9 A**).

Therefore, even if the truncated DFT models on geraniol (**Fig. S6**) suggest that C1 hydroxylation is energetically preferred over C2-C3 epoxidation, MD simulations indicate that the required near-attack conformations for geraniol hydroxylation at C1 are not significantly sampled on Design 2 active site, thus hindering this intrinsically preferred reactivity.

Analysis of the substrate orientation along the MD simulations suggest that the substrate exposes the C2-C3 double bond to the Cpd I species leading to pro-*S* epoxidation (**Fig. S9-C**).

Fig. S10 Analysis of nerol (**2**) binding mode in Design 26 (F63I/F154I/G157A/A161L) through substrate-bound MD simulations.

The following analysis allows to characterize which is the preferred binding pose of the substrate in the active site and to elucidate which position of the substrate is more accessible for effective near-attack conformations (NACs) that resemble the geometric requirements of the DFT optimized TS during the MD simulations. Only substrate positions that exhibit a low energetic cost for oxidation as determined by truncated DFT calculations in **Fig. S7** are analyzed in the MD simulations (C2-C3 epoxidation or C1 hydroxylation).

The following geometric parameters are used to characterize the near-attack conformations in the heat map plots: the O–C2 distance and O–C2=C3 angle are used to characterize NACs for effective C2-C3 epoxidation while O–H1 distance and O–H1–C1 angle are used to characterize NACs for effective C1 hydroxylation.

The resulting heat maps were obtained from 3 independent MD simulations of 500 ns each (a total of 1500 ns) without any restraint on the substrate. Ideal geometric parameters obtained from DFT Transition State (TS) optimizations (**Fig. S7**) are shown as black dots. A schematic representation of the different geometric parameters used for the heat maps are also provided in each subfigure.

A) NACs for C1-HAT leading to nerol hydroxylation.

B) NACs for C2-O formation leading to nerol epoxidation.

C) Polar interaction between the terminal alcohol group of nerol and the sidechain of E158.

D) Most representative structure of the binding mode of nerol in Design 26 active site as determined by clustering analysis.

Distances and angles are given in angstroms (Å) and degrees (º), respectively.

A)

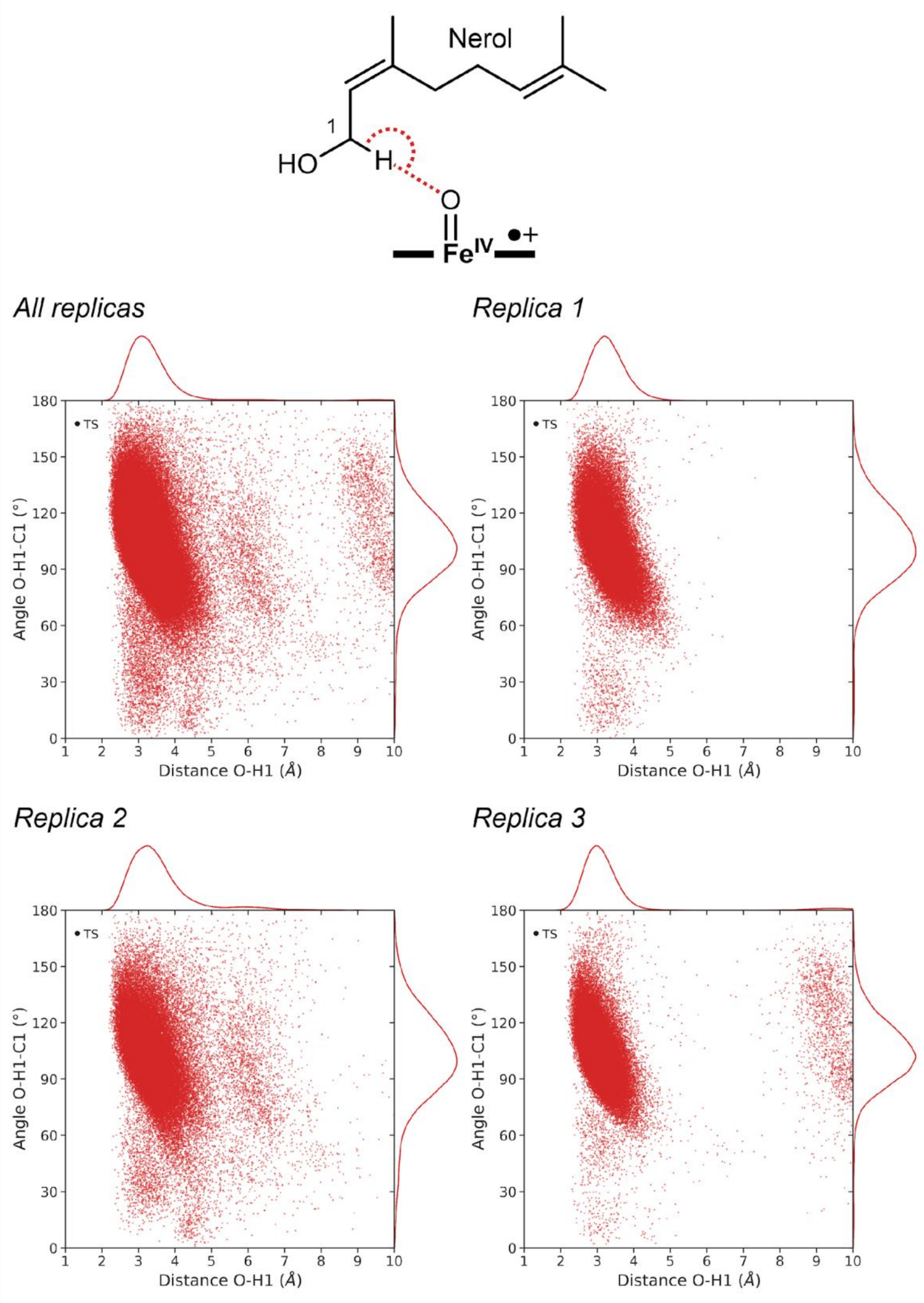

Nerol
1
HO
H
O
Fe^IV
All replicas
Replica 1
Replica 2
Replica 3
TS
Angle O-H1-C1 (°)
Distance O-H1 (Å)

B)

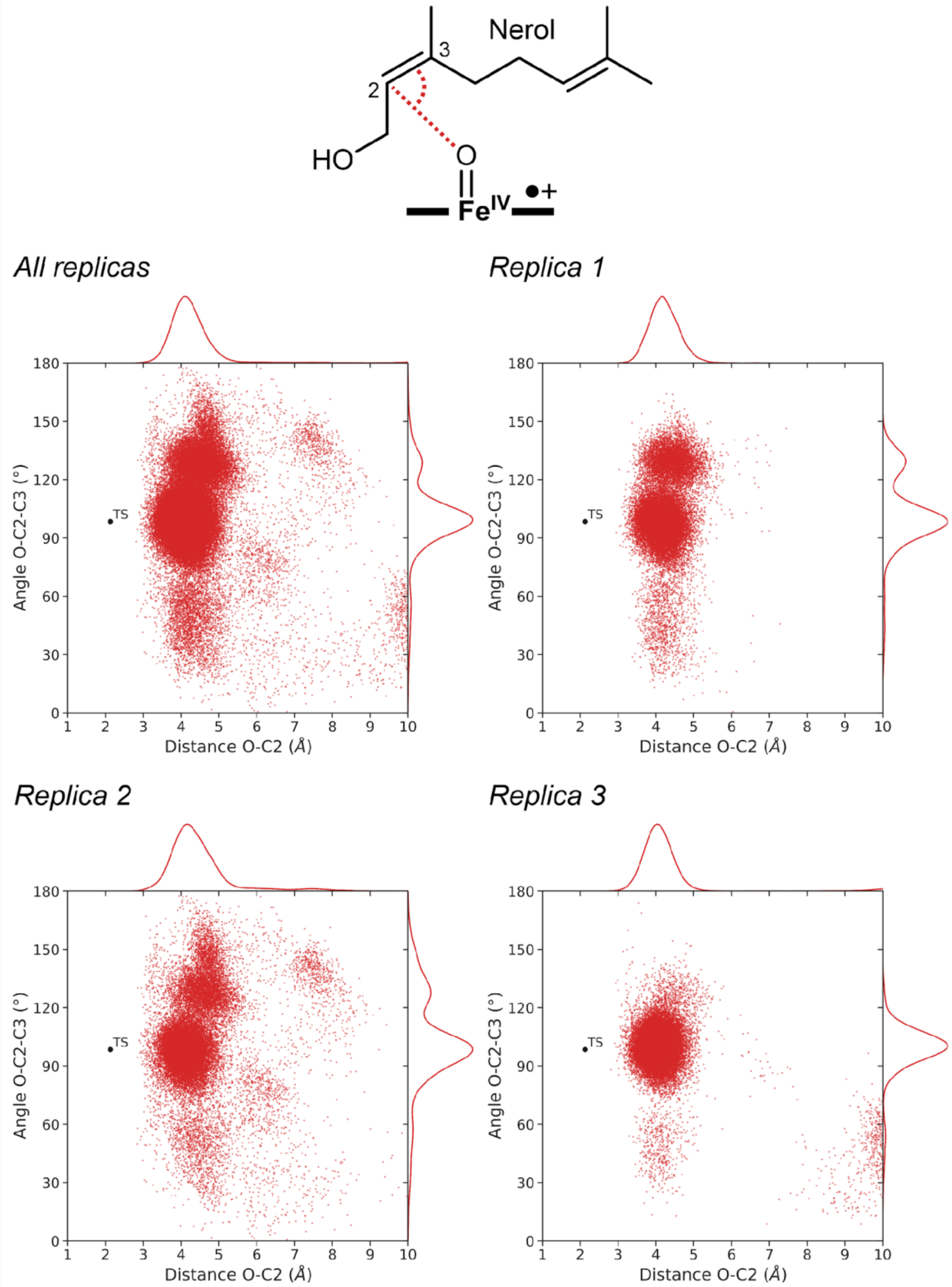

Nerol
3
2
HO
O
Fe^{IV} •+
All replicas
Replica 1
Replica 2
Replica 3
TS
Angle O-C2-C3 (°)
Distance O-C2 (Å)

C)

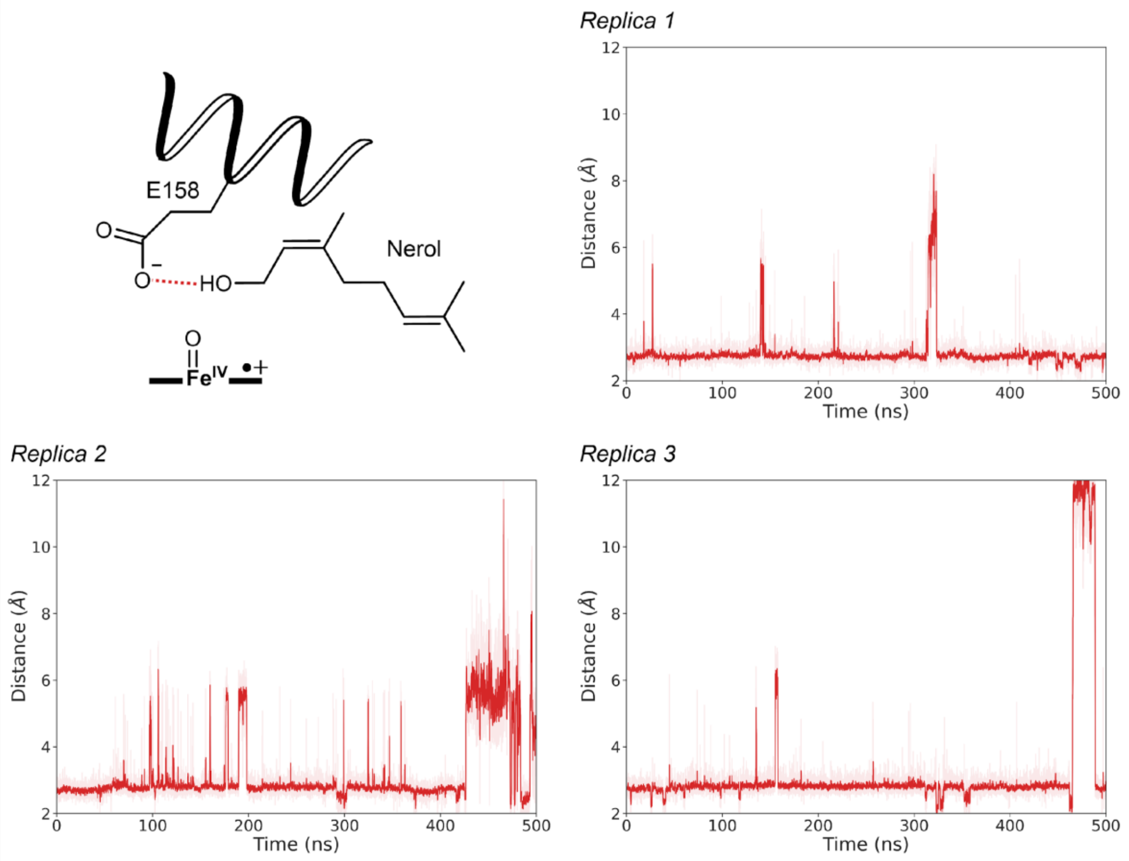

E158
Nerol
HO
Fe^{IV}
Replica 1
Replica 2
Replica 3
Distance (Å)
Time (ns)

D)

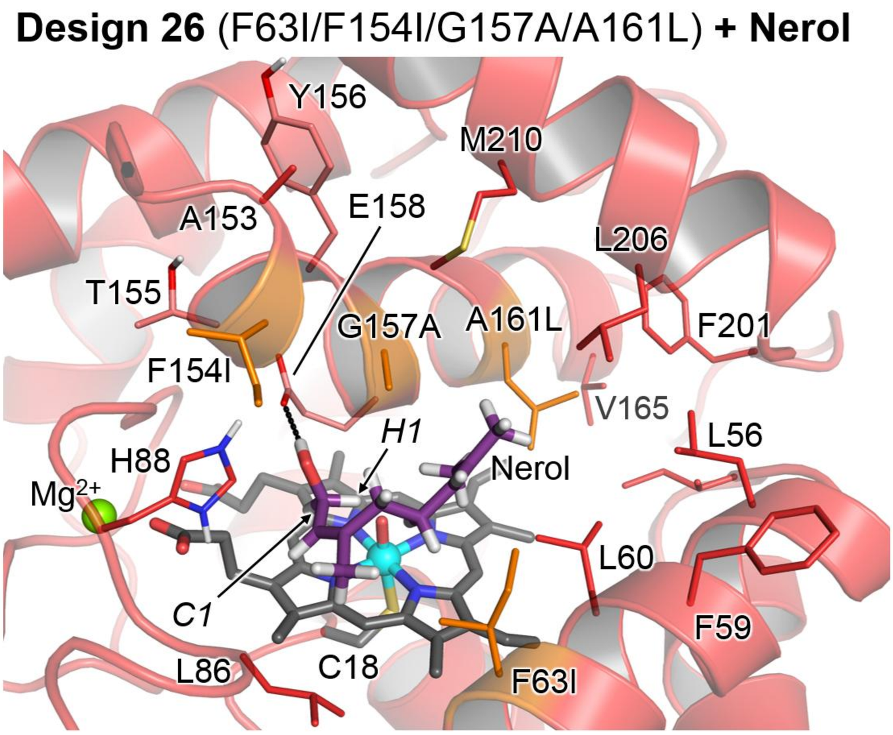

Design 26 (F63I/F154I/G157A/A161L) + Nerol
Y156
M210
A153
E158
L206
T155
G157A
A161L
F201
F154I
V165
L56
H88
H1
Nerol
Mg2+
L60
C1
F59
L86
C18
F63I

The binding mode of nerol in Design 26 is similar to the binding mode of geraniol in Design 4 E158 (**Fig. S10 D** and **Fig. S8 D**). In both cases, the terminal alcohol group of the substrate interact with E158 (**Fig. S10 C** and **Fig. S8 C**).

In addition, position A161 is also mutated by a residue with a bulky sidechain (leucine in Design 26, phenyalanine in Design 4) and the substrate exposes both the C1-H bonds and the C2-C3 double bond (**Figs S10 A** and **B**). As in the truncated DFT model calculation with geraniol, allylic C1 hydroxylation on nerol is energetically preferred, rationalizing the formation of neral as the major experimental product.

No enantioselective preference for pro-*R* or pro-*S* C1 HAT is observed on the MD simulations with nerol bound in the active site of Design 26.

Fig. S11 Analysis of nerol (**2**) binding mode in Design 28 (F63L/F154L/Y156L/A161F) through substrate-bound MD simulations.

The following analysis allows to characterize which is the preferred binding pose of the substrate in the active site and to elucidate which position of the substrate is more accessible for effective near-attack conformations (NACs) that resemble the geometric requirements of the DFT optimized TS during the MD simulations. Only substrate positions that exhibit a low energetic cost for oxidation as determined by truncated DFT calculations in **Fig. S7** are analyzed in the MD simulations (C2-C3 epoxidation or C1 hydroxylation).

The following geometric parameters are used to characterize the near-attack conformations in the heat map plots: the O–C2 distance and O–C2=C3 angle are used to characterize NACs for effective C2-C3 epoxidation while O–H1 distance and O–H1–C1 angle are used to characterize NACs for effective C1 hydroxylation. The O-C2=C3-C4 dihedral angle is used to determine which enantioface of the C2-C3 bond of the substrate is exposed to the catalytic iron-oxo moiety of Cpd I. Positive dihedral values indicate that the pro-*S* face is exposed while negative values indicate that the pro-*R* face is exposed instead.

The resulting heat maps were obtained from 3 independent MD simulations of 500 ns each (a total of 1500 ns) without any restraint on the substrate. Ideal geometric parameters obtained from DFT Transition State (TS) optimizations (**Fig. S7**) are shown as black dots. A schematic representation of the different geometric parameters used for the heat maps are also provided in each subfigure.

A) NACs for C1-HAT leading to nerol hydroxylation.

B) NACs for C2-O formation leading to nerol epoxidation.

C) NACs for pro-*R* and pro-*S* C2-O formation leading to enantioselective nerol epoxidation.

D) Polar interaction between the terminal alcohol group of nerol and iron-oxo group of Cpd I

E) Most representative structure of the binding mode of nerol in Design 28 active site as determined by clustering analysis.

F) Probability to find specific interactions between the backbone atoms of E158 and Y162, the hydroxyl group of the substrate, and solvent molecules in the nerol-bound MD simulations with Design 28. The average value between all the replicas is also provided for each interaction. Additionally, the same analysis is performed for the designs in **Figs. S8** to **S10** to facilitate comparison.

Distances and angles are given in angstroms (Å) and degrees (º), respectively.

A)

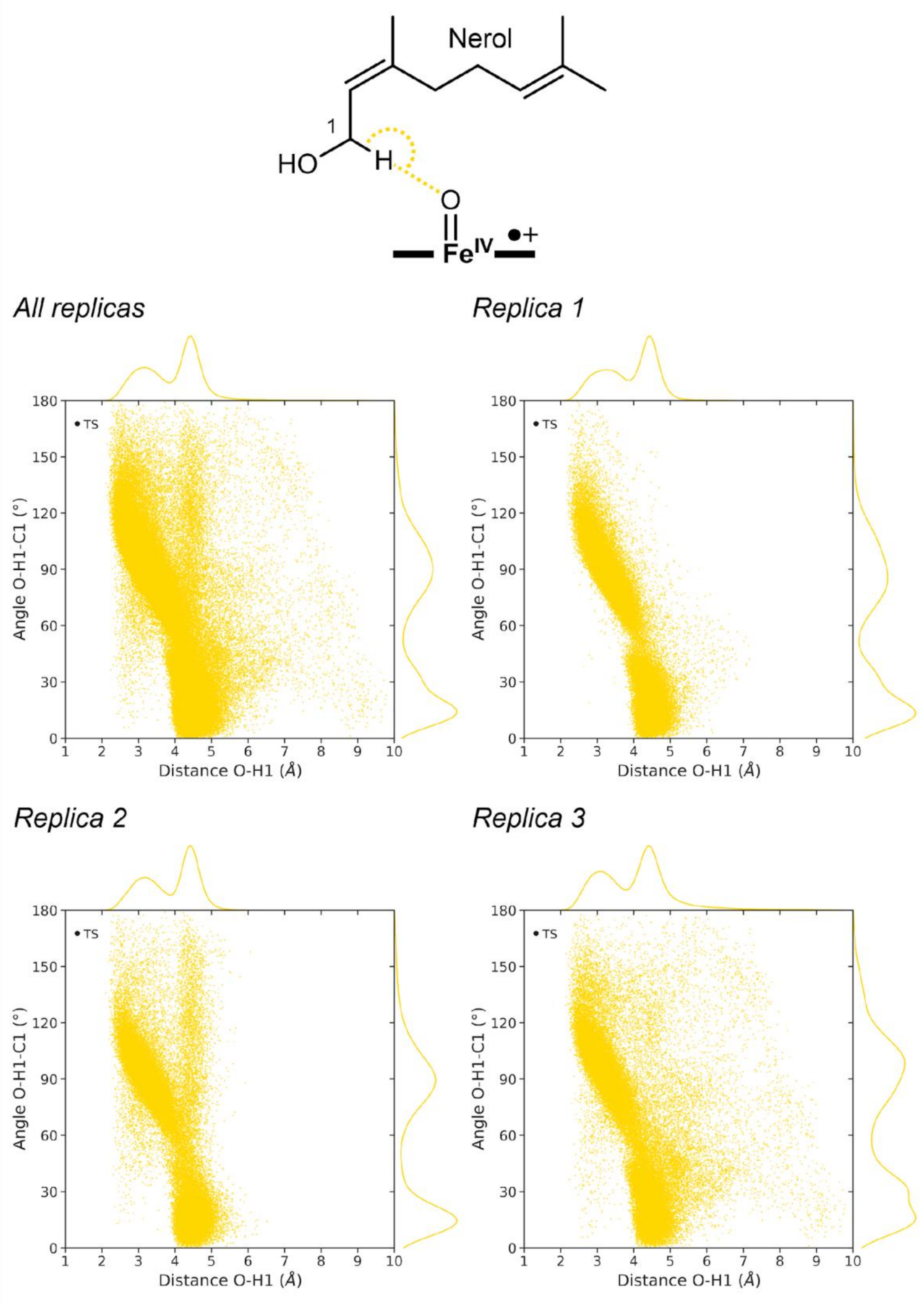
Nerol
1
HO
H
O
Fe
IV
All replicas
Replica 1
Replica 2
Replica 3
TS
Angle O-H1-C1 (°)
Distance O-H1 (Å)
0
30
60
90
120
150
180
1
2
3
4
5
6
7
8
9
10

B)

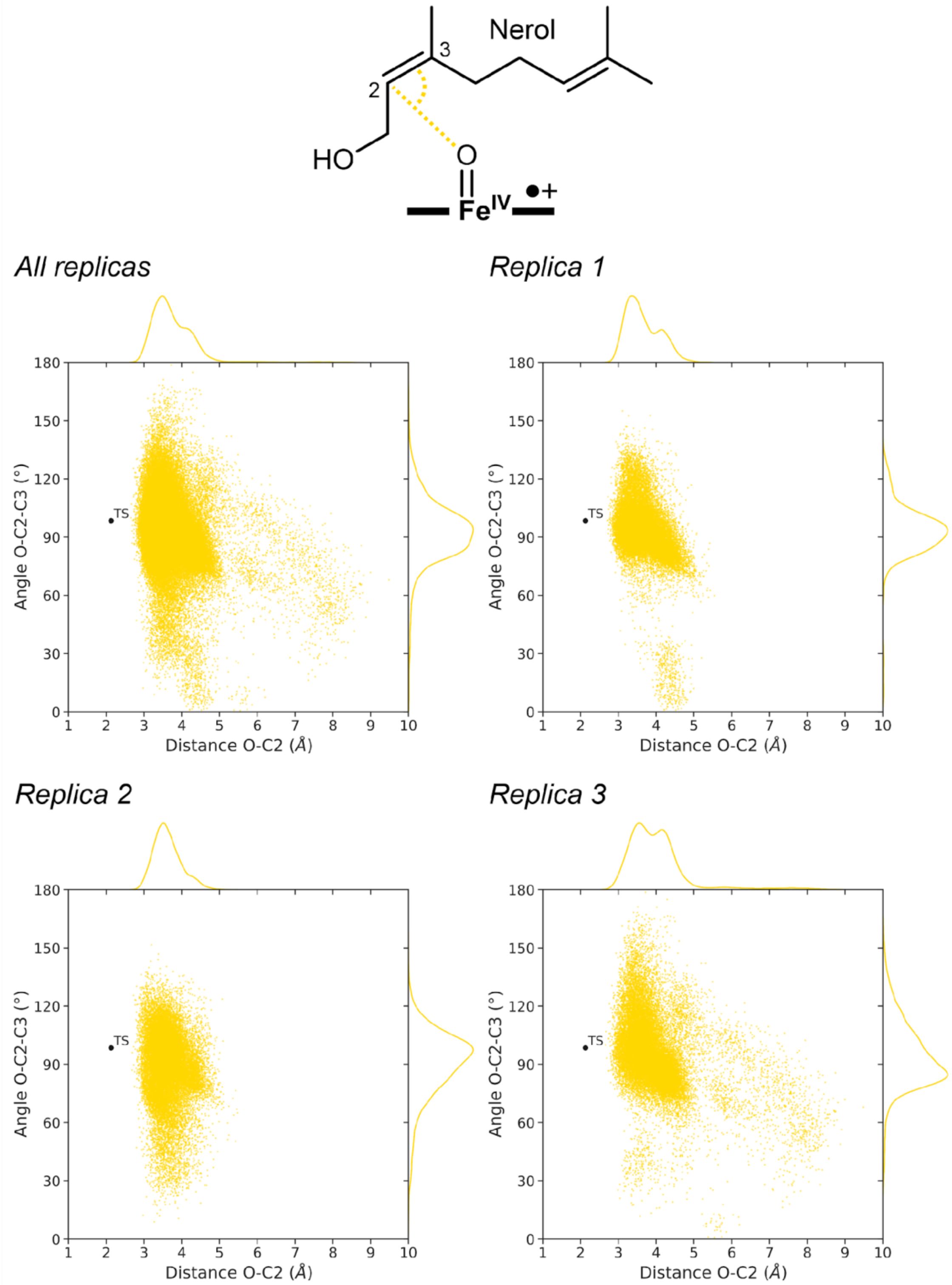
Nerol
3
2
HO
O
Fe^IV
•+
All replicas
Replica 1
Replica 2
Replica 3
TS
Angle O-C2-C3 (°)
Distance O-C2 (Å)
180
150
120
90
60
30
0
1
2
3
4
5
6
7
8
9
10

C)

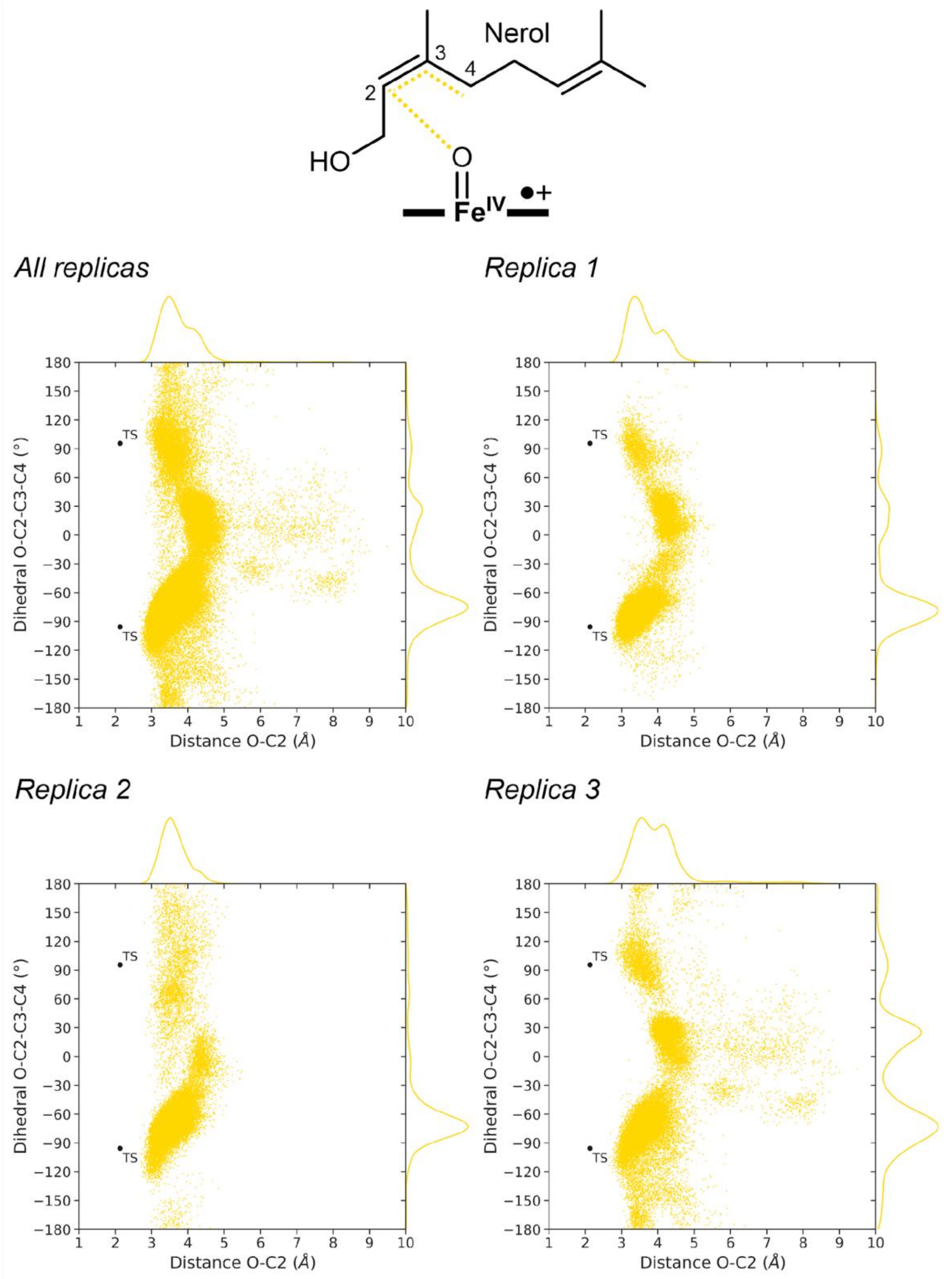
Nerol
3
4
2
HO
O
Fe^IV
•+
All replicas
Replica 1
Replica 2
Replica 3
TS
Dihedral O-C2-C3-C4 (°)
Distance O-C2 (Å)

D)

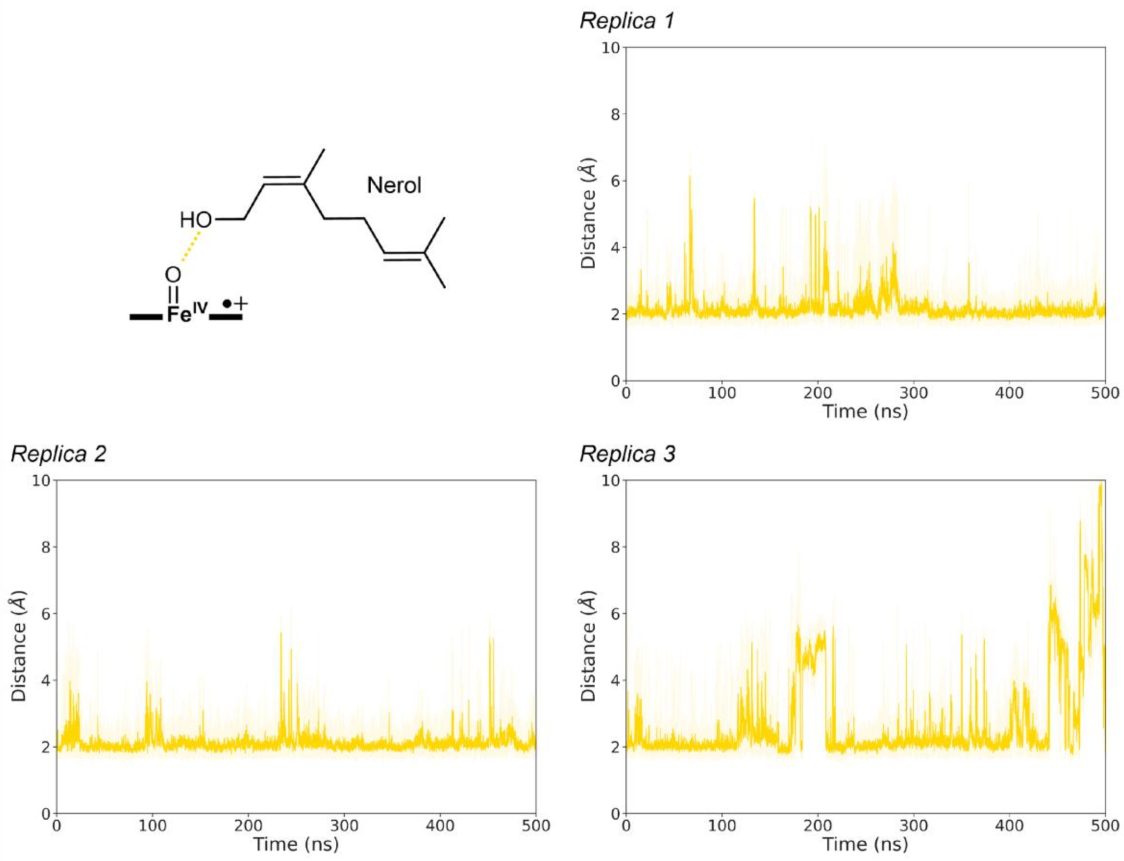

Nerol
HO
O
Fe^{IV} •+
Replica 1
Replica 2
Replica 3
Distance (Å)
Time (ns)
0
2
4
6
8
10
0
100
200
300
400
500

E)

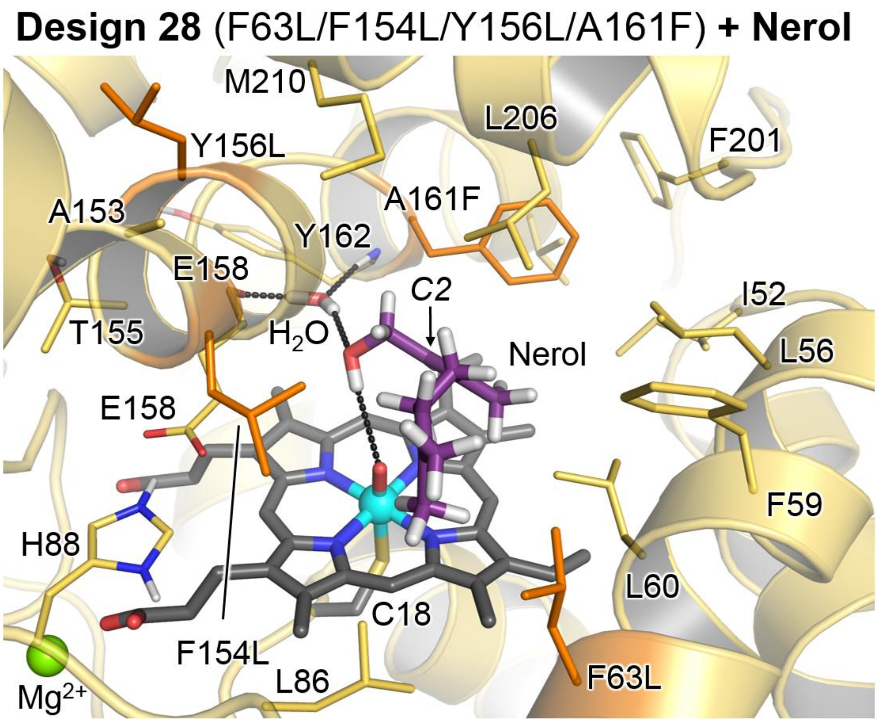

Design 28 (F63L/F154L/Y156L/A161F) + Nerol
M210
L206
F201
Y156L
A153
A161F
Y162
E158
C2
I52
T155
H$_2$O
Nerol
L56
E158
F59
H88
L60
C18
F154L
L86
F63L
Mg^{2+}

F)

Design 28 + Nerol	Replica 1	Replica 2	Replica 3	Average
Direct interaction between E158 - Y162	0	0	0	0.00
Bridging water between E158 - Y162	0.25	0.19	0.20	0.21
Bridging water between E158 - Nerol	0.38	0.30	0.18	0.28
Direct interaction between E158 - H_2O	0.66	0.56	0.61	0.61
Direct interaction between Nerol - H_2O	0.63	0.54	0.61	0.59

Other designs:

Design 26 + Nerol	Replica 1	Replica 2	Replica 3	Average
Direct interaction between E158 - Y162	0.24	0.42	0.61	0.42
Bridging water between E158 - Y162	0	0	0	0.00
Bridging water between E158 - Nerol	0	0	0	0.00
Direct interaction between E158 - H_2O	0	0	0	0.00
Direct interaction between Nerol - H_2O	0.32	0.37	0.46	0.39

Design 4 + Geraniol	Replica 1	Replica 2	Replica 3	Average
Direct interaction between E158 - Y162	0.01	0.01	0.01	0.01
Bridging water between E158 - Y162	0	0	0.04	0.01
Bridging water between E158 - Geraniol	0	0	0	0.00
Direct interaction between E158 - H_2O	0	0	0.09	0.03
Direct interaction between Geraniol-H_2O	0.58	0.69	0.56	0.61

Design 2 + Geraniol	Replica 1	Replica 2	Replica 3	Average
Direct interaction between E158 - Y162	0.15	0.18	0.27	0.20
Bridging water between E158 - Y162	0	0	0	0.00
Bridging water between E158 - 1010	0	0	0	0.00
Direct interaction between E158 - H_2O	0	0	0	0.00
Direct interaction between Geraniol-H_2O	0.46	0.68	0.65	0.60

Nerol binds to Design 28 active site in a novel binding mode not previously detected in any of the previous designs explored by MD simulations in **Figs. S8** to **S10**. This new binding mode is stabilized by an unprecedented direct interaction between the iron-oxo group of Cpd I and the terminal hydroxyl group of the substrate (**Fig. S11 D**) and by the presence of a persistent water molecule bridging the backbone atoms of E158 and Y162 residues, which interacts simultaneously with the alcohol group of nerol (**Figs. S11 E** and **F**). Additional analysis on the previous designs indicates that the presence of this water molecule in Design 28 is due to the mutations introduced in this specific design (mainly by Y156L) rather than an intrinsic feature of all *Mth*UPO designs (**Fig. S11 F**).

As a consequence of this binding pose, the C-H bonds in C1 are not properly oriented to the catalytic moiety (**Fig. S11 A**), positioning C2 in an effective near-attack conformation for C2-C3 epoxidation (**Fig. S11 B**). Analysis of the substrate orientation along the MD simulations suggest that the substrate exposes the C2-C3 double bond to the Cpd I species leading to pro-*R* epoxidation (**Fig. S11 C**).

Fig. S12 First round of FuncLib evolution toward enantiodivergent *S*- and *R*-4-hydroxy-β-ionone formation starting from *Mth*UPO F63I parent enzyme. See Material and Methods section in the SI for further information about the parameters used for FuncLib. See results discussion in the main text.

A) FuncLib evolution round using the F63I *Mth*UPO variant as the parent enzyme. The colour gradient var indicates the Rosetta score difference between the 509 new FuncLib designs and the parent enzyme highlighted in grey (green (red) colour indicates FuncLib designs that have better (worse) Rosetta scores than the parent enzyme). The hits found in our previous study[1] (Münch et al. 2024, Table 1) are also highlighted with black lines.

B) Summary table of the hits detected in our previous study[1] (Münch et al. 2024, Table 1), the mutations that were introduced, the ordering in the FuncLib run, the FuncLib Rosetta score and the enantiomeric ratio (e.r.) are provided. Entries in bold exhibit the highest enantiomeric ratio improvements.

A)

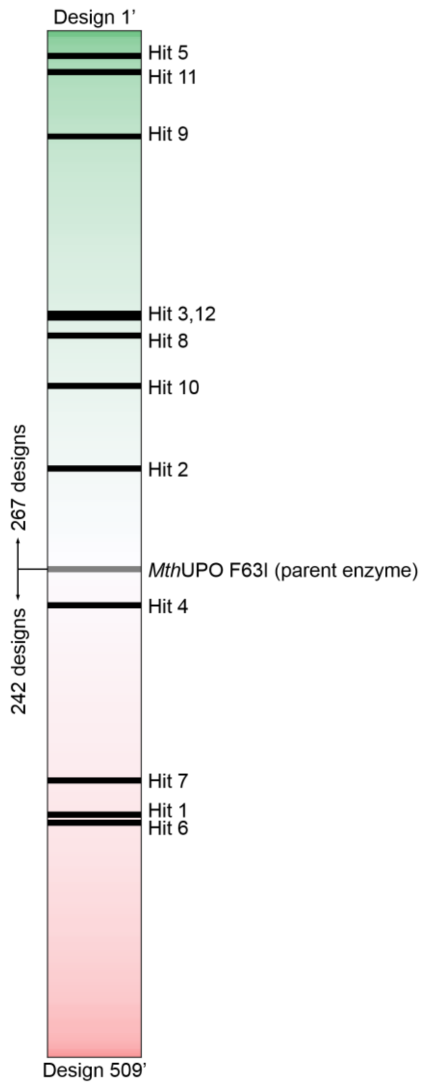

B)

Hits from previous study	Mutations	FuncLib Design	Rosetta score	e.r.
5	I63L, L206F	13'	-11.7	81.3:18.8
**11**	**I63V, F154L**	**21'**	**-10.6**	**7.3:92.7**
9	I63V, F154I	53'	-8.0	12.0:88.0
3	L56I, M210F	142'	-3.7	85.2:14.8
**12**	**L60F, A161V**	**143'**	**-3.7**	**3.3:96.7**
8	L56V, A161I	152'	-3.5	29.4:70.6
10	L60F, A161I	177'	-2.6	11.5:88.5
**2**	**I63L, L206A**	**218'**	**-1.4**	**95.2:4.8**
MthUPO F63I	-	-	0.0	70.7:29.3
4	L206F	285'	0.5	82.0:18.0
7	L56I, M210	372'	2.3	79.5:20.5
**1**	**L206A**	**389'**	**2.8**	**96.6:3.4**
6	L206V	392'	2.8	80.5:19.5

Fig. S13 Second round of FuncLib evolution toward improvement of the *S*-4-hydroxy-β-ionone formation activity starting from *Mth*UPO L60F, F63I, A161V parent enzyme. See Material and Methods section in the SI for further information about the parameters used for FuncLib. See results discussion in the main text.

A) FuncLib evolution round using the L60F, F63I, A161V *Mth*UPO variant as the parent enzyme. The color gradient var indicates the Rosetta score difference between the 197 new FuncLib designs, and the parent enzyme highlighted in gray (green (red) color indicates FuncLib designs that have better (worse) Rosetta scores than the parent enzyme). The hits found in our previous study [1] (Münch et al. 2024, Table 2) are also highlighted with black lines.

B) Summary table of the hits detected in our previous study [1] (Münch et al. 2024, Table 1), the mutations that were introduced, the ordering in the FuncLib run, the FuncLib Rosetta score and the enantiomeric ratio (e.r.) are provided. Entry in bold exhibits the highest enantiometic ratio improvements.

A)

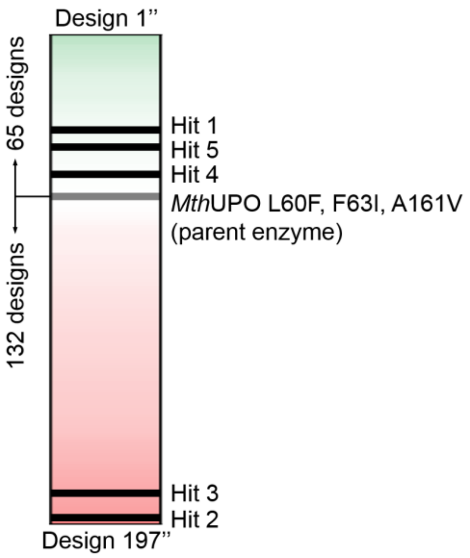

B)

Hit from previous study	Mutations	FuncLib Design	Rosetta score	e.r.
**1**	**F154L**	**39"**	**-1.7**	**0.3:99.7**
5	F154L, M210L	46"	-1.4	0.4:99.6
4	F154I, M210L	57"	-0.6	0.5:99.5
MthUPO L60F, F63I, A161V	-	-	0.0	3.3:96.7
3	F154V	185"	11.4	0.4:99.6
2	F154I	196"	13.9	0.5:99.5

Fig. S14 Design 18 enzyme helical core modelling with Chai-1 docked with several substrates compared to FuncLib design 18.

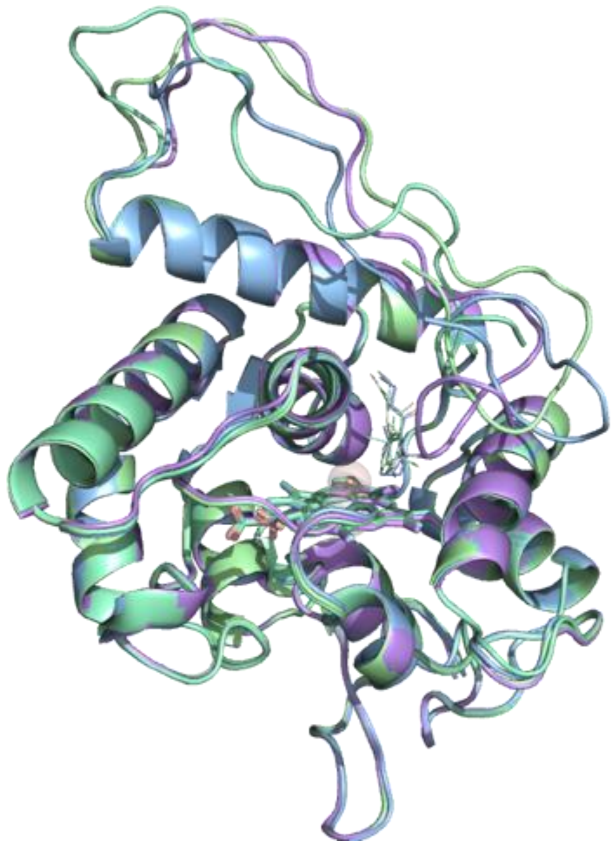

^{1}H-NMR

Isopiperitenol

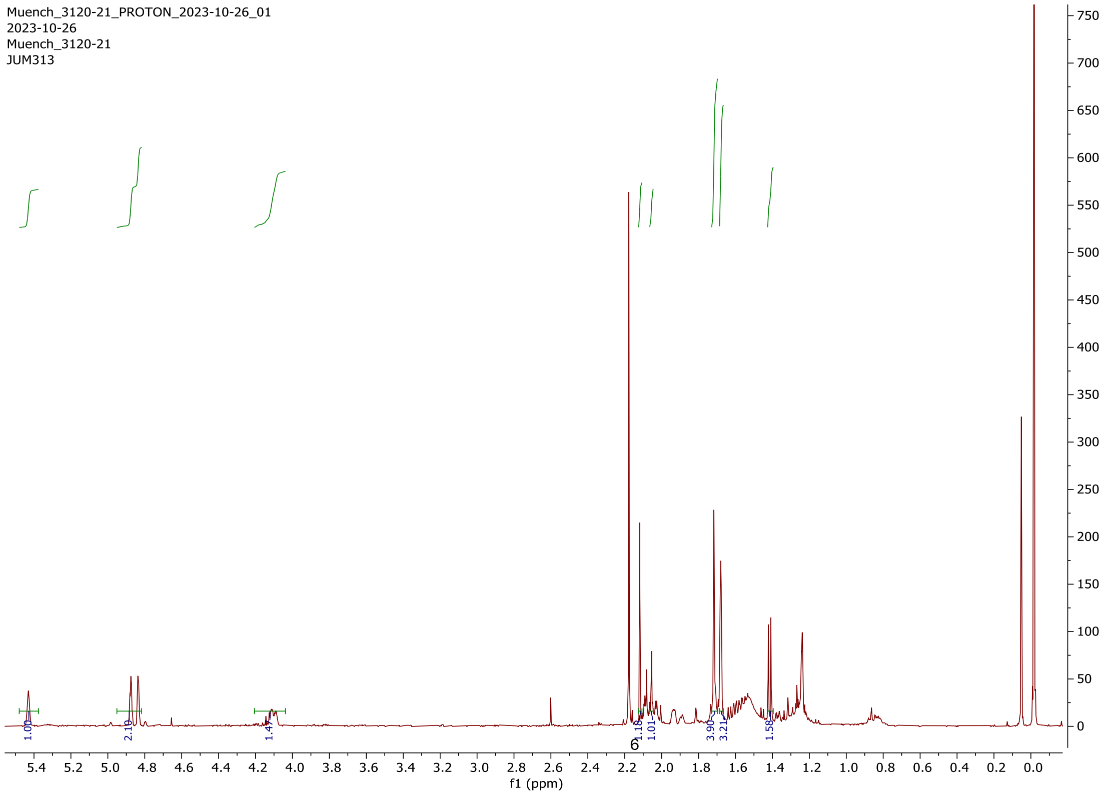

2,3-epoxy nerol

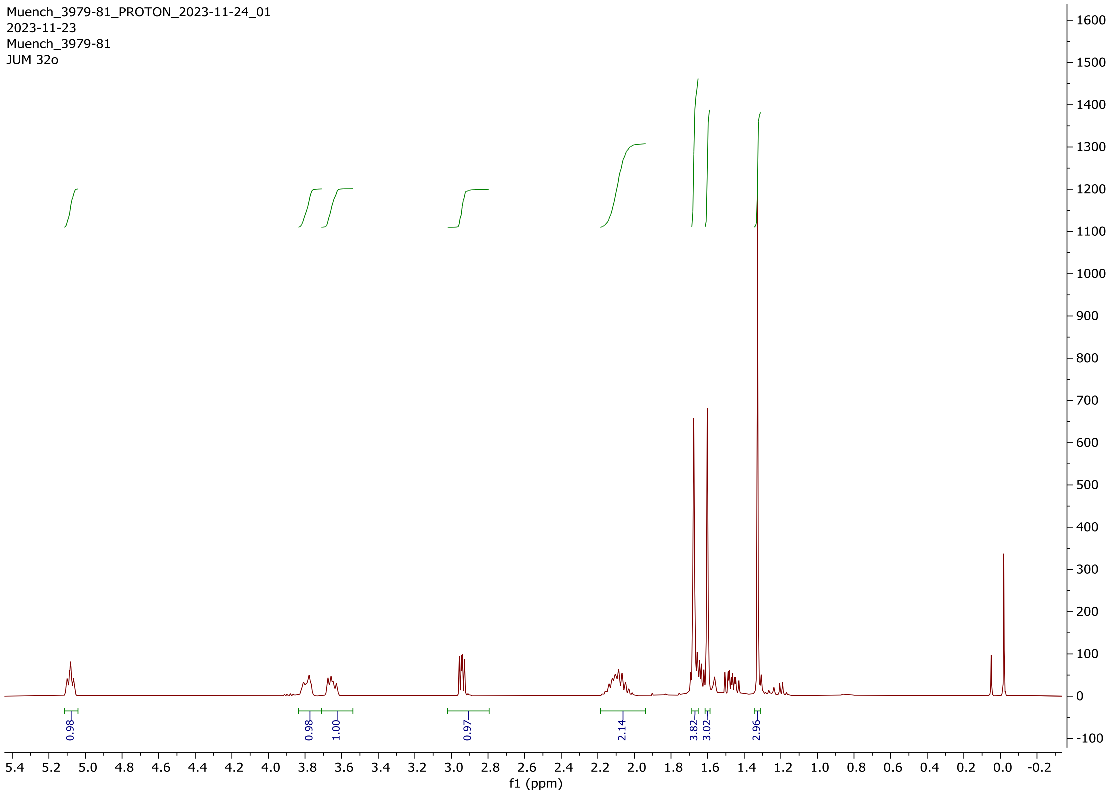

References

(1) Münch, J.; Soler, J.; Hünecke, N.; Homann, D.; Garcia-Borràs, M.; Weissenborn, M. J. Computational-Aided Engineering of a Selective Unspecific Peroxygenase toward Enantiodivergent β-Ionone Hydroxylation. *ACS Catal.* **2023**, *13*, 8963-8972.

(2) Babot, E. D.; Aranda, C.; del Río, J. C.; Ullrich, R.; Kiebist, J.; Scheibner, K.; Hofrichter, M.; Martínez, A. T.; Gutiérrez, A. Selective oxygenation of ionones and damascones by fungal peroxygenases. *JAFC* **2020**, *68* (19), 5375-5383.

(3) Litzenburger, M.; Bernhardt, R. Selective oxidation of carotenoid-derived aroma compounds by CYP260B1 and CYP267B1 from Sorangium cellulosum So ce56. *Appl. Microbiol. Biotechnol.* **2016**, *100*, 4447-4457.

(4) Martins, R. R.; Neves, M. G.; Silvestre, A. J.; Simões, M. M.; Silva, A. M.; Tomé, A. C.; Cavaleiro, J. A.; Tagliatesta, P.; Crestini, C. Oxidation of unsaturated monoterpenes with hydrogen peroxide catalysed by manganese (III) porphyrin complexes. *J. Mol. Catal. Chem.* **2001**, *172* (1-2), 33-42.

(5) Guillon, J.; Rioult, J. P.; Robba, M. New synthesis of isopiperitenol, previously isolated from species of Cymbopogon. *Flavour Frag. J.* **2000**, *15* (4), 223-224.
